# Supplementary figures and images for: Sibling rank and sibling number in relation to cardiovascular disease and mortality risk: a nationwide cohort study
Source: BMJ Open. 2021 May 25;11(6):e042881. doi: 10.1136/bmjopen-2020-042881 (PMC8162087; doi:10.1136/bmjopen-2020-042881)

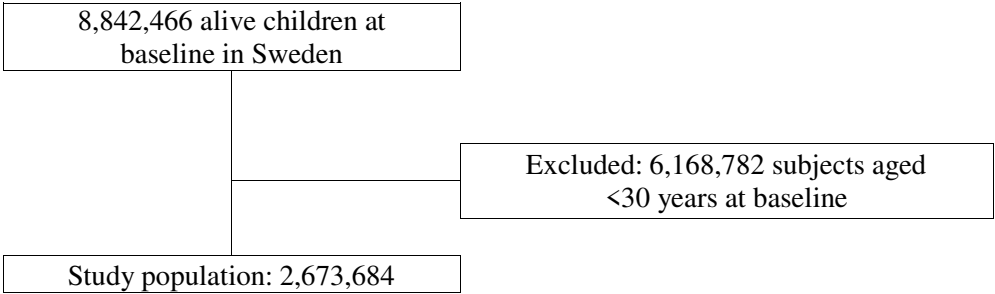

Supplement: Supplementary data [file bmjopen-2020-042881supp002.pdf]
