# Supplementary material for: Sibling rank and sibling number in relation to cardiovascular disease and mortality risk: a nationwide cohort study
Source: BMJ Open. 2021 May 25;11(6):e042881. doi: 10.1136/bmjopen-2020-042881 (PMC8162087; doi:10.1136/bmjopen-2020-042881)
Supplement: Supplementary data [file bmjopen-2020-042881supp001.pdf]

Supplementary Table 1a. Distribution of population, number of CVD, CHD, and mortality events in *men*.

	Population		CVD events		CHD events		Mortality events	
	No.	(%)	No.	%	No.	%	No.	%
Total population (%)	1358647		592863		131533		240371	
Age (years)								
30-39	531559	39.1	152615	25.7	25327	19.3	39094	16.3
40-49	554137	40.8	265251	44.7	59088	44.9	96549	40.2
50-58	272951	20.1	174997	29.5	47118	35.8	104728	43.6
Family income								
Low income	334083	24.6	123575	20.8	26649	20.3	47582	19.8
Middle–low income	320284	23.6	135148	22.8	29613	22.5	51243	21.3
Middle–high income	321653	23.7	152589	25.7	35041	26.6	66400	27.6
High income	382627	28.2	181551	30.6	40230	30.6	75146	31.3
Marital status								
Married/cohabiting	809314	59.6	370633	62.5	85212	64.8	124039	51.6
Never married, Widowed, or divorced	549333	40.4	222230	37.5	46321	35.2	116332	48.4
Educational attainment								
≤ 9 years	281207	20.7	146179	24.7	38890	29.6	81428	33.9
10–11 years	173208	12.7	67512	11.4	13955	10.6	26218	10.9
≥ 12 years	904232	66.6	379172	64.0	78688	59.8	132725	55.2
Immigrant status								
Sweden	1322658	97.4	580707	97.9	128732	97.9	235464	98.0
Other countries	35989	2.6	12156	2.1	2801	2.1	4907	2.0
Socioeconomic status								
Farmers/self-employed/others	308729	22.7	129408	21.8	29064	22.1	71960	29.9
Blue collar workers	494090	36.4	217215	36.6	50626	38.5	89989	37.4
White collar workers	351621	25.9	157170	26.5	34594	26.3	52582	21.9
Professionals	204207	15.0	89070	15.0	17249	13.1	25840	10.8
Urban/rural status								
Large cities	495681	36.5	207593	35.0	41153	31.3	85765	35.7
Southern Sweden	618686	45.5	274100	46.2	61581	46.8	108663	45.2
Northern Sweden	244280	18.0	111170	18.8	28799	21.9	45943	19.1
Hospitalization of alcoholism and related liver disease								
No	1275305	93.9	548796	92.6	124051	94.3	202724	84.3
Yes	83342	6.1	44067	7.4	7482	5.7	37647	15.7
Hospitalization of diabetes								
No	1226642	90.3	494000	83.3	104716	79.6	200226	83.3
Yes	132005	9.7	988863	16.7	26817	20.4	40145	16.7

Hospitalization of hypertension								
No	1059935	78.0	294151	49.6	76609	58.2	183958	76.5
Yes	298712	22.0	298712	50.4	54924	41.8	56413	23.5
Hospitalization of obesity								
No	1338892	98.5	577543	97.4	128595	97.8	236644	98.4
Yes	19755	1.5	15320	2.6	2938	2.2	3727	1.6
Hospitalization of chronic lower respiratory disease								
No	1288108	94.8	546393	92.2	120575	91.7	216522	90.1
Yes	70539	5.2	46470	7.8	10958	8.3	23849	9.9
Cancer								
No	1109571	81.7	452446	76.3	103202	78.5	139486	58.0
Yes	249076	18.3	140417	23.7	28331	21.5	100885	42.0
Number of sibling								
Non sibling	214700	15.8	105516	17.8	23671	18.0	50709	21.1
One sibling	443877	32.7	189839	32.0	39729	30.2	73140	30.4
Two siblings	338812	24.9	140361	23.7	30184	22.9	52790	22.0
Three siblings	183067	13.5	77378	13.1	17663	13.4	30266	12.6
Four or more siblings	178191	13.1	79769	13.5	20286	15.4	33466	13.9
Birth order								
First	684765	50.4	318341	53.7	70238	53.4	140857	58.6
Second	402879	29.7	166757	28.1	36654	27.9	62267	25.9
Third	164540	12.1	65853	11.1	14736	11.2	23081	9.6
Fourth	62765	4.6	24729	4.2	5737	4.4	8425	3.5
Fifth+	43698	3.2	17183	2.9	4168	3.2	5741	2.4

Supplementary Table 1b. Distribution of population, number of CVD, CHD, and mortality events in *women.*

	Population		CVD events		CHD events		Mortality events	
	No.	(%)	No.	%	No.	%	No.	%
Total population (%)	1315037		486147		55933		160269	
Age (years)								
30-39	507872	38.6	125326	25.8	9681	17.3	24571	15.3
40-49	537035	40.8	210113	43.2	23738	42.4	64055	40.0
50+	270130	20.5	150708	31.0	22514	40.3	71643	44.7
Family income								
Low income	343296	26.1	100542	20.7	10467	18.7	29723	18.5
Middle–low income	340837	25.9	121716	25.0	13432	24.0	37773	23.6
Middle–high income	347955	26.5	142924	29.4	17579	31.4	50537	31.5
High income	282949	21.5	120965	24.9	14455	25.8	42236	26.4
Marital status								
Married/cohabiting	887258	67.5	331886	68.3	38106	68.1	96533	60.2
Never married, Widowed, or divorced	427779	32.5	154261	31.7	17827	31.9	63736	39.8
Educational attainment								
≤ 9 years	232824	17.7	102037	21.0	15415	27.6	48455	30.2
10–11 years	165367	12.6	60209	12.4	6867	12.3	20489	12.8
≥ 12 years	916846	69.7	323901	66.6	33651	60.2	91325	57.0
Immigrant status								
Sweden	1281472	97.4	476075	97.9	54756	97.9	157515	98.3
Other countries	33565	2.6	10072	2.1	1177	2.1	2754	1.7
Socioeconomic status								
Farmers/self-employed/others	272189	20.7	96279	19.8	12450	22.3	45553	28.4
Blue collar workers	437324	33.3	174549	35.9	21821	39.0	55960	34.9
White collar workers	496782	37.8	178249	36.7	18324	32.8	49169	30.7
Professionals	108742	8.3	37070	7.6	3338	6.0	9587	6.0
Urban/rural status								
Large cities	495097	37.6	173872	35.8	17499	31.3	57794	36.1
Southern Sweden	591636	45.0	223375	45.9	25965	46.4	72872	45.5
Northern Sweden	228304	17.4	88900	18.3	12469	22.3	29603	18.5
Hospitalization of alcoholism and related liver disease								
No	1283561	97.6	471088	96.9	54416	97.3	149212	93.1
Yes	31476	2.4	15059	3.1	1517	2.7	11057	6.9
Hospitalization of diabetes								
No	1234361	93.9	429522	88.4	45537	81.4	139644	87.1

Yes	80676	6.1	56625	11.6	10396	18.6	20625	12.9
Hospitalization of hypertension								
No	1067928	81.2	239038	49.2	31364	56.1	124898	77.9
Yes	247109	18.8	247109	50.8	24569	43.9	35371	22.1
Hospitalization of obesity								
No	1286838	97.9	468228	96.3	54003	96.5	156771	97.8
Yes	28199	2.1	17919	3.7	1930	3.5	3498	2.2
Hospitalization of chronic lower respiratory disease								
No	1226846	93.3	433906	89.3	48084	86.0	137028	85.5
Yes	88191	6.7	52241	10.7	7849	14.0	23241	14.5
Cancer								
No	1051617	80.0	364226	74.9	43617	78.0	70300	43.9
Yes	263420	20.0	121921	25.1	12316	22.0	89969	56.1
Number of sibling								
Non sibling	210121	16.0	87261	17.9	10289	18.4	34521	21.5
One sibling	430315	32.7	154154	31.7	16280	29.1	49132	30.7
Two siblings	324379	24.7	113739	23.4	12500	22.3	34843	21.7
Three siblings	176631	13.4	63871	13.1	7512	13.4	19766	12.3
Four or more siblings	173591	13.2	67122	13.8	9352	16.7	22007	13.7
Birth order								
First	664459	50.5	262015	53.9	30342	54.2	94779	59.1
Second	388391	29.5	136263	28.0	15191	27.2	40984	25.6
Third	159311	12.1	53711	11.0	6231	11.1	15205	9.5
Fourth	60676	4.6	20264	4.2	2379	4.3	5558	3.5
Fifth+	42200	3.2	13894	2.9	1790	3.2	3743	2.3

Supplementary Table 2a. Hazard ratio (HR) and 95% confidence interval of CVD in *men*, using multivariable competing risk survival analysis

	Model 1			Model 2			Model 3		
	HR*	95% CI		HR*	95% CI		HR*	95% CI	
Number of siblings (ref. No sibling)									
One sibling	0.97	0.97	0.98	0.98	0.97	0.98	0.98	0.97	0.99
Two siblings	0.97	0.96	0.98	0.97	0.96	0.97	0.97	0.97	0.98
Three siblings	0.99	0.98	1.00	0.98	0.97	0.99	0.98	0.97	0.99
Four or more children	1.04	1.03	1.05	1.00	0.99	1.01	1.00	0.99	1.01
Age (years)	1.07	1.07	1.07	1.08	1.07	1.08	1.06	1.06	1.06
Family income (ref. High)									
Low income				0.92	0.92	0.93	0.99	0.98	1.00
Middle–low income				1.02	1.01	1.03	1.05	1.04	1.06
Middle–high income				1.04	1.03	1.05	1.05	1.04	1.06
Educational level (ref. > 12 years)									
10–11 years				1.11	1.10	1.12	1.06	1.05	1.07
≥ 12 years				1.00	1.00	1.01	1.00	0.99	1.00
Socioeconomic status (ref. professionals)									
Farmers/self-employed/others				1.22	1.21	1.23	1.17	1.16	1.18
Blue collar workers				1.15	1.14	1.16	1.09	1.08	1.10
White collar workers				1.08	1.07	1.08	1.04	1.04	1.05
Region of residence (ref. Large cities)									
Southern Sweden				1.00	1.00	1.01	1.03	1.02	1.03
Northern Sweden				1.04	1.03	1.05	1.05	1.04	1.06
Immigrant status (ref. Born in Sweden)				0.95	0.93	0.97	0.96	0.95	0.98
Marital status (ref. Not married)				1.18	1.17	1.19	1.17	1.16	1.18
Birth order				1.01	1.01	1.01	1.01	1.01	1.02
Hospitalization of chronic lower respiratory disease (ref. Non)							1.26	1.25	1.28
Hospitalization of alcoholisms and related liver disease (ref. Non)							1.74	1.73	1.76
Hospitalization of diabetes (ref. Non)							1.39	1.38	1.40
Hospitalization of obesity (ref. Non)							1.37	1.35	1.40
Hospitalization of hypertension (ref. Non)							3.72	3.70	3.74
Cancer (ref. Non)							1.36	1.35	1.36

Model 1. Adjusted for age at start; Model 2. Adjusted for age at start and individual characteristics; Model 3. Model 2 + comorbidities.
*: Multivariable competing risk survival analysis

Supplementary Table 2b. Hazard ratio (HR) and 95% confidence interval of CVD in *men*, using multivariable competing risk survival analysis

	Model 1			Model 2			Model 3		
	HR*	95% CI		HR*	95% CI		HR*	95% CI	
Birth order (ref. First birth)									
Second	0.99	0.99	1.00	0.99	0.98	1.00	1.00	1.00	1.01
Third	1.02	1.01	1.03	1.01	1.00	1.02	1.02	1.02	1.03
Fourth	1.05	1.03	1.06	1.03	1.01	1.04	1.04	1.02	1.05
Fifth+	1.10	1.08	1.12	1.07	1.05	1.08	1.07	1.05	1.09
Age (years)	1.07	1.07	1.07	1.08	1.07	1.08	1.06	1.06	1.06
Family income (ref. High)									
Low income				0.92	0.92	0.93	0.99	0.98	1.00
Middle–low income				1.02	1.01	1.03	1.05	1.04	1.06
Middle–high income				1.04	1.03	1.05	1.05	1.04	1.06
Educational level (ref. > 12 years)									
10–11 years				1.11	1.10	1.12	1.06	1.05	1.07
≥ 12 years				1.00	1.00	1.01	1.00	0.99	1.00
Socioeconomic status (ref. professionals)									
Farmers/self-employed/others				1.22	1.21	1.23	1.17	1.16	1.18
Blue collar workers				1.15	1.14	1.16	1.09	1.08	1.10
White collar workers				1.08	1.07	1.08	1.04	1.04	1.05
Region of residence (ref. Large cities)									
Southern Sweden				1.00	1.00	1.01	1.03	1.02	1.03
Northern Sweden				1.04	1.03	1.05	1.05	1.04	1.06
Immigrant status (ref. Born in Sweden)				0.95	0.93	0.97	0.97	0.95	0.98
Marital status (ref. Not married)				1.18	1.17	1.19	1.17	1.16	1.18
Number of siblings				1.00	1.00	1.00	1.00	1.00	1.00
Hospitalization of chronic lower respiratory disease (ref. Non)							1.26	1.25	1.28
Hospitalization of alcoholisms and related liver disease (ref. Non)							1.74	1.73	1.76
Hospitalization of diabetes (ref. Non)							1.39	1.38	1.40
Hospitalization of obesity (ref. Non)							1.37	1.35	1.40
Hospitalization of hypertension (ref. Non)							3.72	3.70	3.74
Cancer (ref. Non)							1.36	1.35	1.36

Model 1. Adjusted for age at start; Model 2. Adjusted for age at start and individual characteristics; Model 3. Model 2 + comorbidities.
*: Multivariable competing risk survival analysis

Supplementary Table 2c. Hazard ratio (HR) and 95% confidence interval of CVD in *women*, using multivariable competing risk survival analysis

	Model 1			Model 2			Model 3		
	HR*	95% CI		HR*	95% CI		HR*	95% CI	
Number of siblings (ref. No sibling)									
One sibling	0.98	0.97	0.98	0.98	0.97	0.98	0.98	0.98	0.99
Two siblings	0.99	0.98	0.99	0.98	0.97	0.99	0.99	0.98	1.00
Three siblings	1.01	1.00	1.02	1.00	0.99	1.01	1.00	0.99	1.01
Four or more children	1.06	1.05	1.07	1.03	1.02	1.04	1.01	1.00	1.03
Age (years)	1.06	1.06	1.07	1.06	1.06	1.06	1.04	1.04	1.04
Family income (ref. High)									
Low income				0.87	0.86	0.87	0.95	0.94	0.96
Middle–low income				1.01	1.00	1.02	1.04	1.03	1.05
Middle–high income				1.02	1.01	1.03	1.03	1.02	1.03
Educational level (ref. > 12 years)									
10–11 years				1.13	1.12	1.14	1.08	1.06	1.09
≥ 12 years				1.04	1.03	1.05	1.03	1.02	1.04
Socioeconomic status (ref. professionals)									
Farmers/self-employed/others				1.30	1.28	1.31	1.20	1.19	1.22
Blue collar workers				1.25	1.24	1.27	1.12	1.11	1.13
White collar workers				1.08	1.07	1.10	1.03	1.02	1.04
Region of residence (ref. Large cities)									
Southern Sweden				1.03	1.03	1.04	1.03	1.03	1.04
Northern Sweden				1.06	1.06	1.07	1.04	1.03	1.05
Immigrant status (ref. Born in Sweden)				0.97	0.96	0.99	0.98	0.96	1.00
Marital status (ref. Not married)				1.13	1.12	1.14	1.10	1.09	1.11
Birth order				1.00	1.00	1.01	1.01	1.01	1.01
Hospitalization of chronic lower respiratory disease (ref. Non)							1.38	1.36	1.39
Hospitalization of alcoholisms and related liver disease (ref. Non)							1.67	1.65	1.69
Hospitalization of diabetes (ref. Non)							1.38	1.37	1.39
Hospitalization of obesity (ref. Non)							1.32	1.30	1.34
Hospitalization of hypertension (ref. Non)							4.86	4.83	4.89
Cancer (ref. Non)							1.62	1.61	1.63

Model 1. Adjusted for age at start; Model 2. Adjusted for age at start and individual characteristics; Model 3. Model 2 + comorbidities.

*: Multivariable competing risk survival analysis

Supplementary Table 2d. Hazard ratio (HR) and 95% confidence interval of CVD in *women*, using multivariable competing risk survival analysis

	Model 1			Model 2			Model 3		
	HR*	95% CI		HR*	95% CI		HR*	95% CI	
Birth order (ref. First birth)									
Second	1.00	0.99	1.01	0.99	0.98	1.00	1.01	1.00	1.02
Third	1.03	1.02	1.04	1.00	0.99	1.01	1.02	1.01	1.03
Fourth	1.06	1.04	1.07	1.01	1.00	1.03	1.04	1.02	1.05
Fifth+	1.09	1.07	1.10	1.01	0.99	1.03	1.05	1.03	1.07
Age (years)	1.07	1.07	1.07	1.06	1.06	1.06	1.04	1.04	1.04
Family income (ref. High)									
Low income				0.87	0.86	0.87	0.95	0.94	0.96
Middle–low income				1.01	1.00	1.02	1.04	1.03	1.05
Middle–high income				1.02	1.01	1.03	1.03	1.02	1.03
Educational level (ref. > 12 years)									
10–11 years				1.13	1.12	1.14	1.08	1.06	1.09
≥ 12 years				1.04	1.03	1.05	1.03	1.02	1.04
Socioeconomic status (ref. professionals)									
Farmers/self-employed/others				1.30	1.28	1.31	1.21	1.19	1.22
Blue collar workers				1.26	1.24	1.27	1.12	1.11	1.13
White collar workers				1.09	1.07	1.10	1.03	1.02	1.05
Region of residence (ref. Large cities)									
Southern Sweden				1.03	1.03	1.04	1.03	1.03	1.04
Northern Sweden				1.06	1.06	1.07	1.04	1.03	1.05
Immigrant status (ref. Born in Sweden)				0.97	0.96	0.99	0.98	0.96	1.00
Marital status (ref. Not married)				1.13	1.13	1.14	1.10	1.09	1.11
Number of siblings				1.01	1.01	1.01	1.00	1.00	1.01
Hospitalization of chronic lower respiratory disease (ref. Non)							1.38	1.36	1.39
Hospitalization of alcoholisms and related liver disease (ref. Non)							1.67	1.65	1.69
Hospitalization of diabetes (ref. Non)							1.38	1.37	1.39
Hospitalization of obesity (ref. Non)							1.32	1.30	1.34
Hospitalization of hypertension (ref. Non)							4.86	4.83	4.89
Cancer (ref. Non)							1.62	1.61	1.63

Model 1. Adjusted for age at start; Model 2. Adjusted for age at start and individual characteristics; Model 3. Model 2 + comorbidities.
*: Multivariable competing risk survival analysis

Supplementary Table 3a. Hazard ratio (HR) and 95% confidence interval of CHD in *men*

	Model 1			Model 2			Model 3		
	HR	95% CI		HR	95% CI		HR	95% CI	
Number of siblings (ref. No sibling)									
One sibling	1.00	0.98	1.02	0.99	0.97	1.01	0.99	0.97	1.01
Two siblings	1.04	1.03	1.06	1.00	0.98	1.02	1.01	0.99	1.03
Three siblings	1.12	1.10	1.14	1.04	1.02	1.06	1.04	1.02	1.07
Four or more children	1.27	1.25	1.30	1.10	1.08	1.12	1.10	1.07	1.12
Age (years)	1.08	1.08	1.08	1.08	1.08	1.08	1.07	1.07	1.07
Family income (ref. High)									
Low income				0.93	0.91	0.94	0.96	0.95	0.98
Middle–low income				1.00	0.98	1.01	1.02	1.00	1.03
Middle–high income				1.01	1.00	1.03	1.02	1.00	1.03
Educational level (ref. > 12 years)									
10–11 years				1.05	1.03	1.07	1.04	1.02	1.07
≥ 12 years				0.94	0.93	0.95	0.95	0.94	0.96
Socioeconomic status (ref. professionals)									
Farmers/self-employed/others				1.23	1.20	1.25	1.18	1.16	1.21
Blue collar workers				1.26	1.24	1.28	1.21	1.19	1.23
White collar workers				1.18	1.16	1.20	1.15	1.13	1.17
Region of residence (ref. Large cities)									
Southern Sweden				1.11	1.10	1.12	1.12	1.11	1.14
Northern Sweden				1.32	1.30	1.34	1.31	1.29	1.33
Immigrant status (ref. Born in Sweden)				1.09	1.05	1.13	1.10	1.06	1.14
Marital status (ref. Not married)				1.00	0.99	1.01	0.99	0.98	1.01
Birth order				1.05	1.04	1.05	1.05	1.05	1.06
Hospitalization of chronic lower respiratory disease (ref. Non)							1.29	1.26	1.31
Hospitalization of alcoholisms and related liver disease (ref. Non)							1.01	0.99	1.04
Hospitalization of diabetes (ref. Non)							1.75	1.73	1.78
Hospitalization of obesity (ref. Non)							1.13	1.09	1.17
Hospitalization of hypertension (ref. Non)							1.86	1.84	1.88
Cancer (ref. Non)							0.91	0.89	0.92

Model 1. Adjusted for age at start; Model 2. Adjusted for age at start and individual characteristics; Model 3. Model 2 + comorbidities.

Supplementary Table 3b. Hazard ratio (HR) and 95% confidence interval of CHD in *men*

	Model 1			Model 2			Model 3		
	HR	95% CI		HR	95% CI		HR	95% CI	
Birth order (ref. First birth)									
Second	1.09	1.08	1.10	1.06	1.05	1.07	1.08	1.06	1.09
Third	1.19	1.17	1.21	1.11	1.09	1.13	1.13	1.11	1.15
Fourth	1.28	1.25	1.32	1.15	1.12	1.19	1.17	1.14	1.21
Fifth+	1.44	1.39	1.48	1.22	1.17	1.26	1.23	1.19	1.28
Age (years)	1.08	1.08	1.09	1.08	1.08	1.08	1.07	1.07	1.07
Family income (ref. High)									
Low income				0.93	0.91	0.94	0.96	0.95	0.98
Middle–low income				1.00	0.98	1.01	1.02	1.00	1.03
Middle–high income				1.01	1.00	1.02	1.02	1.00	1.03
Educational level (ref. > 12 years)									
10–11 years				1.05	1.03	1.08	1.05	1.03	1.07
≥ 12 years				0.94	0.93	0.95	0.95	0.94	0.97
Socioeconomic status (ref. professionals)									
Farmers/self-employed/others				1.23	1.20	1.25	1.18	1.16	1.21
Blue collar workers				1.26	1.24	1.28	1.21	1.19	1.23
White collar workers				1.18	1.16	1.20	1.15	1.13	1.17
Region of residence (ref. Large cities)									
Southern Sweden				1.11	1.10	1.13	1.12	1.11	1.14
Northern Sweden				1.32	1.30	1.34	1.31	1.29	1.33
Immigrant status (ref. Born in Sweden)				1.09	1.05	1.13	1.10	1.06	1.14
Marital status (ref. Not married)				1.00	0.99	1.01	0.99	0.98	1.01
Number of siblings				1.02	1.02	1.02	1.02	1.02	1.02
Hospitalization of chronic lower respiratory disease (ref. Non)							1.29	1.26	1.31
Hospitalization of alcoholisms and related liver disease (ref. Non)							1.01	0.99	1.04
Hospitalization of diabetes (ref. Non)							1.75	1.73	1.78
Hospitalization of obesity (ref. Non)							1.13	1.09	1.17
Hospitalization of hypertension (ref. Non)							1.86	1.84	1.88
Cancer (ref. Non)							0.91	0.89	0.92

Model 1. Adjusted for age at start; Model 2. Adjusted for age at start and individual characteristics; Model 3. Model 2 + comorbidities.

Supplementary Table 3c. Hazard ratio (HR) and 95% confidence interval of CHD in *women*

	Model 1			Model 2			Model 3		
	HR	95% CI		HR	95% CI		HR	95% CI	
Number of siblings (ref. No sibling)									
One sibling	0.99	0.97	1.02	0.98	0.96	1.01	0.99	0.97	1.02
Two siblings	1.07	1.04	1.10	1.03	1.00	1.05	1.03	1.00	1.06
Three siblings	1.17	1.13	1.20	1.08	1.04	1.11	1.07	1.04	1.11
Four or more children	1.40	1.36	1.44	1.20	1.16	1.24	1.17	1.13	1.21
Age (years)	1.09	1.09	1.09	1.09	1.08	1.09	1.07	1.07	1.07
Family income (ref. High)									
Low income				0.85	0.83	0.88	0.91	0.88	0.93
Middle–low income				1.00	0.98	1.03	1.00	0.98	1.03
Middle–high income				1.02	1.00	1.05	1.02	0.99	1.04
Educational level (ref. > 12 years)									
10–11 years				1.07	1.04	1.10	1.06	1.03	1.09
≥ 12 years				0.92	0.90	0.94	0.94	0.92	0.96
Socioeconomic status (ref. professionals)									
Farmers/self-employed/others				1.52	1.46	1.59	1.37	1.31	1.42
Blue collar workers				1.47	1.42	1.53	1.33	1.28	1.38
White collar workers				1.19	1.14	1.23	1.14	1.10	1.18
Region of residence (ref. Large cities)									
Southern Sweden				1.14	1.12	1.16	1.15	1.13	1.17
Northern Sweden				1.40	1.37	1.44	1.38	1.34	1.41
Immigrant status (ref. Born in Sweden)				1.13	1.07	1.20	1.13	1.07	1.20
Marital status (ref. Not married)				1.12	1.10	1.14	1.08	1.06	1.10
Birth order				1.04	1.03	1.05	1.05	1.04	1.06
Hospitalization of chronic lower respiratory disease (ref. Non)							1.69	1.65	1.73
Hospitalization of alcoholisms and related liver disease (ref. Non)							1.17	1.11	1.23
Hospitalization of diabetes (ref. Non)							2.20	2.15	2.25
Hospitalization of obesity (ref. Non)							1.11	1.06	1.17
Hospitalization of hypertension (ref. Non)							2.22	2.18	2.26
Cancer (ref. Non)							0.94	0.93	0.96

Model 1. Adjusted for age at start; Model 2. Adjusted for age at start and individual characteristics; Model 3. Model 2 + comorbidities.

Supplementary Table 3d. Hazard ratio (HR) and 95% confidence interval of CHD in *women*

	Model 1			Model 2			Model 3		
	HR	95% CI		HR	95% CI		HR	95% CI	
Birth order (ref. First birth)									
Second	1.10	1.07	1.12	1.05	1.02	1.07	1.07	1.05	1.09
Third	1.24	1.21	1.28	1.11	1.08	1.14	1.14	1.11	1.18
Fourth	1.33	1.27	1.38	1.11	1.06	1.16	1.14	1.09	1.20
Fifth+	1.56	1.48	1.63	1.17	1.11	1.24	1.22	1.15	1.29
Age (years)	1.09	1.09	1.09	1.09	1.08	1.09	1.07	1.07	1.07
Family income (ref. High)									
Low income				0.85	0.83	0.88	0.91	0.88	0.93
Middle–low income				1.00	0.98	1.03	1.01	0.98	1.03
Middle–high income				1.02	1.00	1.05	1.02	0.99	1.04
Educational level (ref. > 12 years)									
10–11 years				1.07	1.04	1.10	1.06	1.03	1.09
≥ 12 years				0.92	0.90	0.94	0.94	0.92	0.96
Socioeconomic status (ref. professionals)									
Farmers/self-employed/others				1.52	1.46	1.59	1.37	1.31	1.42
Blue collar workers				1.47	1.42	1.53	1.33	1.28	1.38
White collar workers				1.19	1.14	1.23	1.14	1.10	1.18
Region of residence (ref. Large cities)									
Southern Sweden				1.14	1.12	1.17	1.15	1.13	1.17
Northern Sweden				1.40	1.37	1.44	1.38	1.34	1.41
Immigrant status (ref. Born in Sweden)				1.13	1.07	1.20	1.13	1.07	1.20
Marital status (ref. Not married)				1.12	1.10	1.14	1.08	1.06	1.10
Number of siblings				1.04	1.03	1.04	1.03	1.03	1.04
Hospitalization of chronic lower respiratory disease (ref. Non)							1.69	1.65	1.73
Hospitalization of alcoholisms and related liver disease (ref. Non)							1.17	1.11	1.23
Hospitalization of diabetes (ref. Non)							2.20	2.15	2.25
Hospitalization of obesity (ref. Non)							1.11	1.06	1.17
Hospitalization of hypertension (ref. Non)							2.22	2.18	2.26
Cancer (ref. Non)							0.94	0.93	0.96

Model 1. Adjusted for age at start; Model 2. Adjusted for age at start and individual characteristics; Model 3. Model 2 + comorbidities.

Supplementary Table 4a. Hazard ratio (HR) and 95% confidence interval of mortality in *men*

	Model 1			Model 2			Model 3		
	HR	95% CI		HR	95% CI		HR	95% CI	
Number of siblings (ref. No sibling)									
One sibling	0.91	0.90	0.92	0.92	0.91	0.93	0.93	0.92	0.94
Two siblings	0.92	0.91	0.93	0.91	0.90	0.92	0.91	0.90	0.92
Three siblings	0.96	0.94	0.97	0.93	0.92	0.94	0.93	0.92	0.94
Four or more children	1.03	1.01	1.04	0.96	0.94	0.97	0.96	0.94	0.97
Age (years)	1.10	1.10	1.10	1.11	1.11	1.11	1.10	1.10	1.10
Family income (ref. High)									
Low income				1.05	1.04	1.07	1.08	1.07	1.10
Middle–low income				1.17	1.15	1.18	1.14	1.13	1.15
Middle–high income				1.18	1.17	1.19	1.15	1.14	1.16
Educational level (ref. > 12 years)									
10–11 years				1.12	1.10	1.13	1.08	1.06	1.09
≥ 12 years				0.91	0.90	0.91	0.89	0.88	0.90
Socioeconomic status (ref. professionals)									
Farmers/self-employed/others				1.73	1.71	1.76	1.63	1.60	1.65
Blue collar workers				1.38	1.36	1.40	1.34	1.32	1.36
White collar workers				1.15	1.13	1.17	1.14	1.12	1.16
Region of residence (ref. Large cities)									
Southern Sweden				0.94	0.93	0.95	0.99	0.98	1.00
Northern Sweden				0.98	0.97	0.99	1.08	1.07	1.09
Immigrant status (ref. Born in Sweden)				1.04	1.01	1.07	1.06	1.03	1.09
Marital status (ref. Not married)				1.88	1.87	1.90	1.71	1.70	1.73
Birth order				1.00	1.00	1.01	0.99	0.99	1.00
Hospitalization of chronic lower respiratory disease (ref. Non)							1.28	1.26	1.30
Hospitalization of alcoholisms and related liver disease (ref. Non)							3.02	2.98	3.05
Hospitalization of diabetes (ref. Non)							1.43	1.41	1.45
Hospitalization of obesity (ref. Non)							1.09	1.06	1.13
Hospitalization of hypertension (ref. Non)							0.66	0.65	0.67
Cancer (ref. Non)							2.62	2.60	2.65

Model 1. Adjusted for age at start; Model 2. Adjusted for age at start and individual characteristics; Model 3. Model 2 + comorbidities.

Supplementary Table 4b. Hazard ratio (HR) and 95% confidence interval of mortality in *men*

	Model 1			Model 2			Model 3		
	HR	95% CI		HR	95% CI		HR	95% CI	
Birth order (ref. First birth)									
Second	0.98	0.97	0.99	0.97	0.96	0.98	0.96	0.95	0.97
Third	1.01	0.99	1.02	0.99	0.98	1.01	0.98	0.96	0.99
Fourth	1.04	1.01	1.06	1.00	0.98	1.03	0.98	0.95	1.00
Fifth+	1.11	1.08	1.14	1.06	1.02	1.09	1.01	0.98	1.05
Age (years)	1.10	1.10	1.10	1.11	1.11	1.11	1.10	1.10	1.10
Family income (ref. High)									
Low income				1.06	1.04	1.07	1.08	1.07	1.10
Middle–low income				1.17	1.15	1.18	1.14	1.13	1.16
Middle–high income				1.18	1.17	1.19	1.15	1.14	1.16
Educational level (ref. > 12 years)									
10–11 years				1.12	1.10	1.13	1.07	1.06	1.09
≥ 12 years				0.90	0.90	0.91	0.89	0.88	0.90
Socioeconomic status (ref. professionals)									
Farmers/self-employed/others				1.73	1.71	1.76	1.63	1.60	1.66
Blue collar workers				1.38	1.36	1.40	1.34	1.32	1.36
White collar workers				1.15	1.13	1.17	1.14	1.13	1.16
Region of residence (ref. Large cities)									
Southern Sweden				0.94	0.93	0.95	0.99	0.98	1.00
Northern Sweden				0.98	0.97	0.99	1.08	1.07	1.09
Immigrant status (ref. Born in Sweden)				1.05	1.02	1.08	1.06	1.03	1.09
Marital status (ref. Not married)				1.88	1.87	1.90	1.71	1.70	1.73
Number of siblings				1.00	0.99	1.00	1.00	0.99	1.00
Hospitalization of chronic lower respiratory disease (ref. Non)							1.28	1.26	1.30
Hospitalization of alcoholisms and related liver disease (ref. Non)							3.02	2.98	3.05
Hospitalization of diabetes (ref. Non)							1.43	1.41	1.45
Hospitalization of obesity (ref. Non)							1.09	1.06	1.13
Hospitalization of hypertension (ref. Non)							0.66	0.65	0.67
Cancer (ref. Non)							2.62	2.60	2.64

Model 1. Adjusted for age at start; Model 2. Adjusted for age at start and individual characteristics; Model 3. Model 2 + comorbidities.

Supplementary Table 4c. Hazard ratio (HR) and 95% confidence interval of mortality in *women*

	Model 1			Model 2			Model 3		
	HR	95% CI		HR	95% CI		HR	95% CI	
Number of siblings (ref. No sibling)									
One sibling	0.92	0.91	0.94	0.93	0.92	0.95	0.94	0.93	0.95
Two siblings	0.92	0.91	0.94	0.91	0.90	0.93	0.92	0.91	0.94
Three siblings	0.95	0.93	0.96	0.92	0.90	0.93	0.93	0.91	0.95
Four or more children	1.01	0.99	1.03	0.94	0.92	0.95	0.95	0.93	0.96
Age (years)	1.10	1.10	1.10	1.10	1.10	1.10	1.09	1.08	1.09
Family income (ref. High)									
Low income				0.84	0.83	0.86	0.94	0.93	0.96
Middle–low income				1.03	1.01	1.05	1.06	1.04	1.07
Middle–high income				1.05	1.04	1.06	1.05	1.04	1.07
Educational level (ref. > 12 years)									
10–11 years				1.08	1.06	1.10	1.01	1.00	1.03
≥ 12 years				0.85	0.84	0.87	0.82	0.81	0.83
Socioeconomic status (ref. professionals)									
Farmers/self-employed/others				1.97	1.93	2.02	1.88	1.84	1.93
Blue collar workers				1.36	1.33	1.39	1.35	1.32	1.38
White collar workers				1.12	1.10	1.15	1.11	1.09	1.14
Region of residence (ref. Large cities)									
Southern Sweden				1.00	0.99	1.01	1.03	1.02	1.04
Northern Sweden				1.03	1.02	1.05	1.11	1.09	1.12
Immigrant status (ref. Born in Sweden)				0.89	0.86	0.92	0.95	0.92	0.99
Marital status (ref. Not married)				1.63	1.62	1.65	1.48	1.47	1.50
Birth order				1.01	1.00	1.01	1.00	0.99	1.01
Hospitalization of chronic lower respiratory disease (ref. Non)							1.61	1.58	1.63
Hospitalization of alcoholisms and related liver disease (ref. Non)							3.26	3.20	3.32
Hospitalization of diabetes (ref. Non)							1.58	1.56	1.61
Hospitalization of obesity (ref. Non)							0.96	0.93	0.99
Hospitalization of hypertension (ref. Non)							0.66	0.65	0.67
Cancer (ref. Non)							4.86	4.81	4.91

Model 1. Adjusted for age at start; Model 2. Adjusted for age at start and individual characteristics; Model 3. Model 2 + comorbidities.

Supplementary Table 4d. Hazard ratio (HR) and 95% confidence interval of mortality in *women*

	Model 1			Model 2			Model 3		
	HR	95% CI		HR	95% CI		HR	95% CI	
Birth order (ref. First birth)									
Second	0.98	0.96	0.99	0.97	0.96	0.98	0.96	0.95	0.98
Third	1.01	1.00	1.03	1.00	0.98	1.02	0.98	0.96	1.00
Fourth	1.05	1.02	1.08	1.02	0.99	1.05	1.00	0.98	1.03
Fifth+	1.11	1.08	1.15	1.08	1.04	1.12	1.03	0.99	1.07
Age (years)	1.10	1.10	1.10	1.10	1.10	1.10	1.09	1.08	1.09
Family income (ref. High)									
Low income				0.84	0.83	0.86	0.94	0.93	0.96
Middle–low income				1.03	1.02	1.05	1.06	1.04	1.07
Middle–high income				1.05	1.04	1.07	1.05	1.04	1.07
Educational level (ref. > 12 years)									
10–11 years				1.08	1.06	1.10	1.01	0.99	1.03
≥ 12 years				0.85	0.84	0.86	0.82	0.81	0.83
Socioeconomic status (ref. professionals)									
Farmers/self-employed/others				1.98	1.93	2.02	1.88	1.84	1.93
Blue collar workers				1.36	1.33	1.39	1.35	1.32	1.38
White collar workers				1.12	1.10	1.15	1.11	1.09	1.14
Region of residence (ref. Large cities)									
Southern Sweden				1.00	0.99	1.01	1.03	1.02	1.04
Northern Sweden				1.03	1.02	1.05	1.11	1.09	1.12
Immigrant status (ref. Born in Sweden)				0.89	0.86	0.92	0.95	0.92	0.99
Marital status (ref. Not married)				1.63	1.62	1.65	1.48	1.47	1.50
Number of siblings				0.99	0.99	0.99	0.99	0.99	1.00
Hospitalization of chronic lower respiratory disease (ref. Non)							1.61	1.58	1.63
Hospitalization of alcoholisms and related liver disease (ref. Non)							3.26	3.20	3.33
Hospitalization of diabetes (ref. Non)							1.58	1.56	1.61
Hospitalization of obesity (ref. Non)							0.96	0.93	0.99
Hospitalization of hypertension (ref. Non)							0.66	0.65	0.67
Cancer (ref. Non)							4.86	4.81	4.91

Model 1. Adjusted for age at start; Model 2. Adjusted for age at start and individual characteristics; Model 3. Model 2 + comorbidities.
