# Supplementary material for: Sibling rank and sibling number in relation to cardiovascular disease and mortality risk: a nationwide cohort study
Source: BMJ Open. 2021 May 25;11(6):e042881. doi: 10.1136/bmjopen-2020-042881 (PMC8162087; doi:10.1136/bmjopen-2020-042881)
Supplement: Author's manuscript [file bmjopen-2020-042881.draft_revisions.pdf]

BMJ Open

BMJ Open is committed to open peer review. As part of this commitment we make the peer review history of every article we publish publicly available.

When an article is published we post the peer reviewers' comments and the authors' responses online. We also post the versions of the paper that were used during peer review. These are the versions that the peer review comments apply to.

The versions of the paper that follow are the versions that were submitted during the peer review process. They are not the versions of record or the final published versions. They should not be cited or distributed as the published version of this manuscript.

BMJ Open is an open access journal and the full, final, typeset and author-corrected version of record of the manuscript is available on our site with no access controls, subscription charges or pay-per-view fees (http://bmjopen.bmj.com).

If you have any questions on BMJ Open's open peer review process please email info.bmjopen@bmj.com

BMJ Open

Sibling rank and sibling number in relation to cardiovascular disease and mortality risk: a nationwide cohort study

Journal:	*BMJ Open*
Manuscript ID	bmjopen-2020-042881
Article Type:	Original research
Date Submitted by the Author:	28-Jul-2020
Complete List of Authors:	Nilsson, Peter; Lunds Universitet, Clinical Sciences<br>Sundquist, Jan; Lund University, Center for Primary Health Care Research;<br>Sundquist, Kristina; Lund University, Center for Primary Health Care Research<br>Li, Xinjun; Lund University, Center for Primary Health Care Research
Keywords:	EPIDEMIOLOGY, REPRODUCTIVE MEDICINE, SOCIAL MEDICINE

SCHOLARONE™
Manuscripts

I, the Submitting Author has the right to grant and does grant on behalf of all authors of the Work (as defined in the below author licence), an exclusive licence and/or a non-exclusive licence for contributions from authors who are: i) UK Crown employees; ii) where BMJ has agreed a CC-BY licence shall apply, and/or iii) in accordance with the terms applicable for US Federal Government officers or employees acting as part of their official duties; on a worldwide, perpetual, irrevocable, royalty-free basis to BMJ Publishing Group Ltd ("BMJ") its licensees and where the relevant Journal is co-owned by BMJ to the co-owners of the Journal, to publish the Work in this journal and any other BMJ products and to exploit all rights, as set out in our licence.

The Submitting Author accepts and understands that any supply made under these terms is made by BMJ to the Submitting Author unless you are acting as an employee on behalf of your employer or a postgraduate student of an affiliated institution which is paying any applicable article publishing charge ("APC") for Open Access articles. Where the Submitting Author wishes to make the Work available on an Open Access basis (and intends to pay the relevant APC), the terms of reuse of such Open Access shall be governed by a Creative Commons licence – details of these licences and which Creative Commons licence will apply to this Work are set out in our licence referred to above.

Other than as permitted in any relevant BMJ Author's Self Archiving Policies, I confirm this Work has not been accepted for publication elsewhere, is not being considered for publication elsewhere and does not duplicate material already published. I confirm all authors consent to publication of this Work and authorise the granting of this licence.

For peer review only

Original manuscript for the BMJ Open 2020 07 28/PN

Sibling rank and sibling number in relation to cardiovascular disease and mortality risk: a nationwide cohort study

Peter M Nilsson [1], Jan Sundquist [1,2], Kristina Sundquist [1,2] and Xinjun Li [1,2]

[1] Department of Clinical Sciences, Lund University, Skåne University Hospital, Malmö, Sweden

[2] Center for Primary Care Research, Lund University, Skåne University Hospital, Malmö, Sweden

For peer review only

**Short title:**	Sibling rank, CVD and mortality risk
**Number of words:**	250 in Abstract, 1879 in full text
**Number of references:**	22
**Number of tables:**	3 + 3 supplemental
**Number of figures:**	3 + 1 supplemental
**Conflict of interest:**	None

Corresponding author:

Peter M Nilsson, MD, PhD

Lund University, Department of Clinical Sciences,

Skåne University Hospital,

Jan Waldenströms gata 15, 5th floor

S-205 02 Malmö, Sweden

Phone: +46-40-33 24 15

e-mail: Peter.Nilsson@med.lu.se

For peer review only - http://bmjopen.bmj.com/site/about/guidelines.xhtml

Abstract

Objective: The number and rank-order of siblings could be of importance for cardiovascular disease (CVD) and mortality. Previous studies have used only fatal events as outcomes. We therefore aimed to use also non-fatal coronary and cardiovascular events.

Methods: From the Multi-Generation Register in Sweden data were used from 1.36 million men and 1.32 million women (born 1932 to 1960), aged 30-58 years at baseline and with follow-up from 1990 to 2015. Mean age at follow-up was 67 years (range 55-83 years). Fatal and non-fatal events were retrieved from national registers.

Results: For men, a higher number of siblings (with no siblings as reference) was associated with an increased risk for CVD (if ≥4 siblings); an increased risk for coronary events (if >3 siblings); but lower total mortality risk (if >1 sibling), following full adjustment. For women, a higher number of siblings was associated with an increased risk CVD (if ≥3 siblings), coronary events (if ≥2 siblings), but lower total mortality (if >1 sibling). First-born men had a lower risk of both cardiovascular and coronary events than their later-born siblings, but higher total mortality than second- and third-born siblings. For first-born women the risks of cardiovascular and coronary events were also lower than in later-born siblings. The mortality risk was higher than for second-born siblings, but equal to higher-numbered siblings.

Conclusion: Being first-born is associated with a favourable effect on non-fatal cardiovascular and coronary events for both men and women. The underlying biological and social mechanisms for this should be studied.

Key words: Cardiovascular, Coronary, Mortality, Register, Risk, Sibling

For peer review only

Strengths and limitations of this study

- This was a national register study with extensive data from several generations on sibling rank linked to high-quality registers on both morbidity and mortality.
- Outcomes were adjusted for confounding factors including individual characteristics (family income, marital status, immigrant background and educational level, region of residence, socioeconomic status) and co-morbidities.
- One limitation was that we had no data on life-style related factors such as body mass index, smoking, and diet, because it would be unrealistic to gather such data for an entire national population.
- Another limitation is that we have no access to psychological or personality data that may be of importance to analyse the impact on health and behaviour of sibling number and rank order.

For peer review only

For peer review only - http://bmjopen.bmj.com/site/about/guidelines.xhtml

Introduction

A positive family history of cardiovascular disease (CVD) is a well-established variable to be used in risk algorithms for CVD, but family history may be difficult to assess based on subjective recall only [1,2]. A better alternative is to use register-based data for a more objective appraisal of the family burden of CVD [3]. One special feature of family structure is the *number of siblings* and *sibling rank* that can also be mapped by use of national registers such as the Multi-Generation Register (MGR) of Sweden [4]. So far this register has been mostly used to describe the risk of some selected CVD manifestations of individuals in relation to the risk of their siblings, for example for thromboembolic disease [5] or other diseases [6-9].

In a previous report based on the MGR it was shown that an increased number of siblings reflecting family size was not associated with increased total and cause-specific mortality risk in ages 40-74 years, but no analyses were made for risk of non-fatal CVD or coronary disease [10]. However, another corresponding study based on MGR data found that total and cause-specific mortality in ages 30-69 years was increased with increasing sibling rank [11].

The influence of sibling rank has been less well studied in relation to *non-fatal* cardiovascular and coronary risk. Previous studies have indicated a worse cardiovascular risk factor burden in first-borns, for example higher body mass index (BMI), higher systolic blood pressure, and lower insulin sensitivity, than later born siblings [12-16]. On the other hand, first-borns seem to have a better physical fitness at military conscript testing [17], less caries [18] and run a lower risk of leukemia in adolescence [19] as well as lower suicide risk in a Finnish population [20], influencing total mortality risk.

Against this background we aimed to analyse the potential influence of sibling number and rank on risk of non-fatal and fatal cardiovascular and coronary events, as well as total mortality, using the MGR of Sweden and after extensive adjustment for potential confounders, such as demographic factors and family social status.

Subjects and methods

Setting and participants

The dataset used in this study was constructed by linking several national Swedish registers. The Swedish government-owned statistics bureau (Statistics Sweden), provided the Multi-Generation Register (MGR), in which persons born in Sweden in or after 1932 (the present study population) are linked to their parents and siblings [4]. Linkages were also made to National Census data in order to ascertain individual-level socioeconomic status. The final link was made by adding data from the Swedish Cause of Death Register (1961-2015) and the Swedish Hospital Discharge Register, with

For peer review only

For peer review only - http://bmjopen.bmj.com/site/about/guidelines.xhtml

recorded dates of hospitalization and hospital diagnoses since 1964, but on a national level since 1987, and in this dataset until the end of 2015.

Ethical approval was obtained from the Ethical Review Board at Lund University.

For analysing risk of CVD, CHD and mortality in relation to number of siblings and sibling rank, we collected data from 1.36 million men and 1.32 million women (born 1932 to 1960), aged 30-58 years at baseline and with follow-up from 1990 to 2015. For the definition of study subjects based on the MGR, see supplemental Figure S1.

Follow-up of CVD events and total mortality

We used the following ICD-codes: fatal or non-fatal CVD (ICD-9, 390-459, ICD-10, I00-I99); and fatal or non-fatal CHD (ICD-9, 410-414, ICD-10, I20-I25). Non-fatal events were followed in the national Hospital Discharge Register, and fatal events and total mortality until 31st December 2015 in the Cause of Death Register.

Definitions

Family income was calculated as annual family income (low, middle-low, middle-high, high) divided by the number of people in the family, taking into consideration the ages of the family members, whereby small children were given lower weights than adolescents and adults.

Immigration status: born in or outside Sweden.

Marital status was classified as married/cohabitating or never married, widowed, or divorced.

Socioeconomic status was divided into four categories: blue collar workers, white collar workers, professionals, or self-employed/farmers/all others,

Education was classified into three categories: ≤ 9 years, 10–11 years, and ≥ 12 years of education.

Geographic region was divided into large Swedish cities (cities with a population of more than 200,000 inhabitants), Southern Sweden, and Northern Sweden.

Co-morbidity was defined as the first hospitalization during the follow-up period of: chronic obstructive pulmonary disease, COPD (ICD-9 490–496 and ICD-10 J40-J49), obesity (ICD-9 278A and ICD-10 E65-E68), alcoholism related liver disease (ICD-9 291, 303, 571 and ICD-10 F10 and K70), hypertension (ICD-9 401-405 and ICD-10 I10–I15), and diabetes (ICD-9 250 and ICD-10 E10-E14), and cancer (ICD-9 140-239, and ICD-10 C00-D48). For COPD and cancer, the Cause of Death Register was also used.

Patient and Public Involvement

No patients were involved with data from medical records, and no public organisations were involved.

For peer review only - http://bmjopen.bmj.com/site/about/guidelines.xhtml

Statistical methods

Person-years at risk were calculated from the start of follow-up on 1 January 1990 until hospitalization or death from CVD, CHD, death from other causes, emigration, or the end of the follow-up, 31st December 2015. Age-adjusted incidence rates for first hospitalization and mortality were calculated for the entire follow-up period. We used the Cox's proportional hazard model to calculate the hazard ratio (HR) with 95 percent confidence intervals (CI) for total (fatal and non-fatal) CVD and CHD events, and for total mortality in both men and women in relation to number of siblings and sibling rank. This was done after adjustment for age at baseline, individual characteristics (family income, marital status, immigrant background and educational level, region of residence, socioeconomic status), and finally for co-morbidities. Individuals without siblings were used as the reference.

A further adjustment was made for total number of siblings in relation to birth order when the risk for different outcomes was calculated, using the category "first born" as reference. A p-value < 0.05 was considered significant.

Results

With an average of 20 years (IQR 16-25 years) of follow-up, in 1,358,647 men, we used data on 592,863 CVD events, 131,533 coronary events, and 240,371 total deaths. For 1,315,037 women, the corresponding numbers were 486,147 CVD events, 55,933 coronary events and 160,269 deaths. The mean age of the study population at the end of the follow-up was 67 years (range 55-83 years). The number of siblings and birth order of men and women are depicted in **Table 1**.

Risk associated with number of siblings in men and women

For men, a higher number of siblings (with no siblings as reference) was associated with a lower risk of CVD (if 1-2 siblings), but an increased risk of CVD (if ≥4 siblings); an increased risk for coronary events (if ≥3 siblings); as well as lower total mortality risk (if ≥1 sibling), following full adjustment. **Table 2**, **Figures 1a-3a.**

For women, a higher number of siblings (with no siblings as reference) was associated with an increased risk of CVD (if ≥3 siblings), coronary events (if ≥2 siblings), but lower total mortality risk (if ≥1 sibling), following full adjustment. **Table 3**, **Figures 1b-3b.**

For peer review only - http://bmjopen.bmj.com/site/about/guidelines.xhtml

Risk associated with sibling rank in men and women

According to sibling rank, first-born men had a lower risk of both cardiovascular and coronary events than their later-born siblings, but higher total mortality than second and third-born siblings, following full adjustment. **Table 2**.

For first-born women the risks of cardiovascular and coronary events were also lower than in their later-born siblings. The mortality risk was higher than for second-born siblings, but equal to higher numbered siblings, following full adjustment. **Table 3**.

For hazard ratios of CHD, CVD and total mortality by number of siblings and birth order in men and women, respectively, see **Figures 1-3.**

For detailed data on the distribution of the study population, number of CVD, coronary heart disease (CHD), and mortality events in men and women, see Supplemental **Table S1**. For data on the risk associated with the factors adjusted for, see Supplemental **Tables S2** for men and **Table S3** for women.

Discussion

In this large-scale follow-up study based on the national Multi-Generation Register it was found that first-born men and women are at lower risk of both cardiovascular and coronary events than their later-born siblings, but at higher total mortality risk than second and third-born siblings in men. For women the mortality risk for first-born women was higher than for second-born siblings, following full adjustment for a number of background factors.

For total mortality in relation to sibling number our data are at odds with a previous study using the same register in Sweden, showing no increased mortality associated until 74 years with a higher number of siblings [10]. However, we used higher numbers, longer follow-up and more extensive adjustments.

For the influence of sibling rank, a previous study showed a higher risk of total and cause-specific mortality with increasing sibling rank until 69 years [11]. This was similar to our study for risk of non-fatal cardiovascular and coronary events during a longer follow-up and extensive adjustments.

These findings of lower cardiovascular risk in first-borns are surprising as previous reports show a higher level of cardiovascular risk factors in such individuals followed until adolescence or young adulthood [12-16]. The burden of risk factors might have been compensated for by a better physical fitness, as noticed in first-born men coming for military conscript testing at the age of around 18 years [17]. In contrast to previous observations, our extensive data indicate a lower cardiovascular risk in first-

For peer review only - http://bmjopen.bmj.com/site/about/guidelines.xhtml

borns. Other unmeasured factors linked to being first-born, such as cognition or bodily development, could have contributed to our findings of a relative protection, even if we adjusted for a long list of potential confounders such as educational level, other socio-economic factors, marital status, and co-morbidities.

Limitations and strengths of the study

The Swedish hospital discharge register contains no information about diagnostic procedures, which is a limitation. However, specialist doctors in hospital care made the diagnosis. Another limitation is that we had no data on life-style related factors such as body mass index, smoking, and diet, because it would be unrealistic to gather such data for an entire national population. However, we did adjust for socioeconomic status, obesity, diabetes, COPD, and alcoholism and related liver disorders, which are associated with factors such as smoking and alcohol. No data reflecting psychology or personality traits were available.

Strengths of the study include complete nationwide coverage from 1990 in a country with high standards of diagnosis, and with diagnoses often being made by specialists during extended examinations in clinics. Another important strength of our study is that it was based on nationwide registers and was thus free of selection and recall bias. The Swedish MGR and the Swedish Hospital Discharge Register are validated data sources that have been proven to be reliable in studies of many diseases [4, 21]. Data in our dataset are almost 100% complete [4].

Future research should be directed to find biological or social mechanisms linking the status of being first-born to lower risk of cardiovascular disease, as indicated by our observational findings. A previous Norwegian study in military conscripts indicated that the role of being first-born is influenced by social factors, as a second-born son may achieve characteristics of a first-born brother who died young [22].

In conclusion, our data indicate a favourable effect on non-fatal cardiovascular and coronary events by being first-born, both for men and women.

For peer review only - http://bmjopen.bmj.com/site/about/guidelines.xhtml

Contributorship statement: PMN: Conceptualisation, Methodology, Writing – Original draft preparation. XL: Methodology, Formal analysis, Writing – Reviewing and Editing. KS: Funding acquisition, Writing – Reviewing and Editing. JS: Writing – Reviewing and Editing. All authors contributed to the interpretation of the analysis and critically revised the manuscript. The corresponding author attests that all listed authors meet authorship criteria and that no others meeting the criteria have been omitted.

Funding: This work was supported by the grants to KS from The Swedish Research Council (2018-02400); the Swedish Heart-Lung foundation (20180465) and ALF funding from Region Skåne. The funders had no role in the analyses, interpretation of results, or the writing of this manuscript.

For peer review only

Competing interests: All authors have completed the ICMJE uniform disclosure form at www.icmje.org/coi_disclosure.pdf and declare no financial relationships with any organisations that might have an interest in the submitted work in the previous three years; no other relationships or activities that could appear to have influenced the submitted work.

Ethical approval: The study was approved by the Regional Ethics Review Board at the Lund University, Sweden (ID number 2012/795; approved 2013-02-06).

Data sharing statement: No additional data available. The manuscript guarantors affirm that this manuscript is an honest, accurate, and transparent account of the study being reported; that no important aspects of the study have been omitted.

Patient and Public Involvement statement: No patients or public organisations were involved in the study design or interpretation.

Dissemination to participants and related patient and public communities: Dissemination to the population, in general, will be through media outreach on the publication of this study.

For peer review only - http://bmjopen.bmj.com/site/about/guidelines.xhtml

References

1. Banerjee A. A review of family history of cardiovascular disease: risk factor and research tool. *Int J Clin Pract*. 2012; 66:536-43.

2. Tada H, Melander O, Louie JZ, *et al*. Risk prediction by genetic risk scores for coronary heart disease is independent of self-reported family history. *Eur Heart J*. 2016;37:561–567.

3. Nilsson PM, Nilsson J-Å, and Berglund G. Family burden of cardiovascular mortality – risk implications for offspring in a national register linkage study based upon the Malmö Preventive Project. *J Internal Med* 2004; 255:229-35.

4. Ekbom A. The Swedish Multi-generation Register. *Methods Mol Biol*. 2011;675:215–220.

5. Zöller B, Ji J, Sundquist J, Sundquist K. Body Height and Incident Risk of Venous Thromboembolism: A Cosibling Design. *Circ Cardiovasc Genet*. 2017;10:e001651.

6. Kasiman K, Lundholm C, Sandin S, *et al*. Familial effects on ischemic stroke: the role of sibling kinship, sex, and age of onset. *Circ Cardiovasc Genet*. 2012;5:226–233.

7. Martinsson A, Li X, Zöller B, *et al*. Familial Aggregation of Aortic Valvular Stenosis: A Nationwide Study of Sibling Risk. *Circ Cardiovasc Genet*. 2017; 10(6):e001742.

8. Lindgren MP, Ji J, Smith JG, Sundquist J, Sundquist K, Zöller B. Mortality risks associated with sibling heart failure [published online ahead of print, 2019 Oct 12]. *Int J Cardiol*. 2019; S0167-5273(19)30895-2.

9. Berntsson J, Li X, Zöller B, *et al*. Risk of Stroke in Patients With Atrial Fibrillation Is Associated With Stroke in Siblings: A Nationwide Study. *J Am Heart Assoc*. 2020; 9(3):e014132.

10. Baranowska-Rataj A, Barclay K, Kolk M. The effect of number of siblings on adult mortality: Evidence from Swedish registers for cohorts born between 1938 and 1972. *Popul Stud (Camb)*. 2017; 71(1):43–63.

11. Barclay K, Kolk M. Birth order and mortality: a population-based cohort study. *Demography*. 2015; 52(2):613–639.

12. Ayyavoo A, Derraik JG, Hofman PL, Cutfield WS. Is being first-born another risk factor for metabolic and cardiovascular diseases? *Future Cardiol*. 2013; 9(4):447–450.

13. Theodore RF, Broadbent J, Nagin D, *et al*. Childhood to Early-Midlife Systolic Blood Pressure Trajectories: Early-Life Predictors, Effect Modifiers, and Adult Cardiovascular Outcomes. *Hypertension*. 2015;66(6):1108–1115.

For peer review only

14. Albert BB, de Bock M, Derraik JG, *et al.* Among overweight middle-aged men, first-borns have lower insulin sensitivity than second-borns. *Sci Rep.* 2014;4:3906.

15. Siervo M, Horta BL, Stephan BC, Victora CG, Wells JC. First-borns carry a higher metabolic risk in early adulthood: evidence from a prospective cohort study. *PLoS One*. 2010; 5(11):e13907.

16. Jelenkovic A, Silventoinen K, Tynelius P, Myrskylä M, Rasmussen F. Association of birth order with cardiovascular disease risk factors in young adulthood: a study of one million Swedish men. *PLoS One.* 2013; 8(5):e63361.

17. Barclay K, Myrskylä M. Birth order and physical fitness in early adulthood: evidence from Swedish military conscription data. *Soc Sci Med.* 2014; 123:141–148.

18. Julihn A, Soares FC, Hammarfjord U, Hjern A, Dahllöf G. Birth order is associated with caries development in young children: a register-based cohort study. *BMC Public Health.* 2020; 20(1):218.

19. Von Behren J, Spector LG, Mueller BA, *et al.* Birth order and risk of childhood cancer: a pooled analysis from five US States. *Int J Cancer.* 2011; 128(11):2709–2716.

20. Saarela J, Cederström A, Rostila M. Birth order and mortality in two ethno-linguistic groups: Register-based evidence from Finland. *Soc Sci Med.* 2016; 158:8–13.

21. Ludvigsson JF, Andersson E, Ekbom A, *et al.* External review and validation of the Swedish national inpatient register. *BMC Public Health.* 2011; 11:450.

22. Kristensen P, Bjerkedal T. Explaining the relation between birth order and intelligence. *Science* 2007; 316(5832):1717.

For peer review only

For peer review only - http://bmjopen.bmj.com/site/about/guidelines.xhtml

Figure legends

Figure 1. Hazard ratios of CHD by number of siblings and birth order in men (a,b) and women (c, d).

Figure 2. Hazard ratios of CVD by number of siblings and birth order in men (a,b) and women (c, d).

Figure 3. Hazard ratios of mortality by number of siblings and birth order in men (a,b) and women (c, d).

For peer review only

For peer review only - http://bmjopen.bmj.com/site/about/guidelines.xhtml

Table 1. Distribution of population, number of CVD, CHD, and mortality events

	Population		CVD events		CHD events		Mortality events	
	No.	(%)	No.	%	No.	%	No.	%
**Men**	1358647		592863		131533		240371	
Number of sibling								
Non sibling	214700	15.8	105516	17.8	23671	18.0	50709	21.1
One sibling	443877	32.7	189839	32.0	39729	30.2	73140	30.4
Two siblings	338812	24.9	140361	23.7	30184	22.9	52790	22.0
Three siblings	183067	13.5	77378	13.1	17663	13.4	30266	12.6
Four or more siblings	178191	13.1	79769	13.5	20286	15.4	33466	13.9
Birth order								
First	684765	50.4	318341	53.7	70238	53.4	140857	58.6
Second	402879	29.7	166757	28.1	36654	27.9	62267	25.9
Third	164540	12.1	65853	11.1	14736	11.2	23081	9.6
Fourth	62765	4.6	24729	4.2	5737	4.4	8425	3.5
Fifth+	43698	3.2	17183	2.9	4168	3.2	5741	2.4
**Women**	1315037		486147		55933		160269	
Number of sibling								
Non sibling	210121	16.0	87261	17.9	10289	18.4	34521	21.5
One sibling	430315	32.7	154154	31.7	16280	29.1	49132	30.7
Two siblings	324379	24.7	113739	23.4	12500	22.3	34843	21.7
Three siblings	176631	13.4	63871	13.1	7512	13.4	19766	12.3
Four or more siblings	173591	13.2	67122	13.8	9352	16.7	22007	13.7
Birth order								
First	664459	50.5	262015	53.9	30342	54.2	94779	59.1
Second	388391	29.5	136263	28.0	15191	27.2	40984	25.6
Third	159311	12.1	53711	11.0	6231	11.1	15205	9.5
Fourth	60676	4.6	20264	4.2	2379	4.3	5558	3.5
Fifth+	42200	3.2	13894	2.9	1790	3.2	3743	2.3

For peer review only

Table 2. Hazard ratio (HR) and 95% confidence interval of CVD, CHD, and mortality in men

	CVD			CHD			Mortality		
	HR	95% CI		HR	95% CI		HR	95% CI	
**Number of siblings (ref. No sibling)**									
One sibling	0.99	0.98	0.99	0.99	0.97	1.01	0.93	0.92	0.94
Two siblings	0.98	0.98	0.99	1.01	0.99	1.03	0.91	0.90	0.92
Three siblings	0.99	0.98	1.00	1.04	1.02	1.07	0.93	0.92	0.94
Four or more siblings	1.01	1.00	1.02	1.10	1.07	1.12	0.96	0.94	0.97
**Birth order (ref. First birth)**									
Second	1.01	1.00	1.01	1.08	1.06	1.09	0.96	0.95	0.97
Third	1.03	1.02	1.04	1.13	1.11	1.15	0.98	0.96	0.99
Fourth	1.04	1.03	1.06	1.17	1.14	1.21	0.98	0.95	1.00
Fifth+	1.07	1.05	1.09	1.23	1.19	1.28	1.02	0.99	1.05

*: Full adjusted model.

For peer review only

Table 3. Hazard ratio (HR) and 95% confidence interval of CVD, CHD, and mortality in women

	CVD			CHD			Mortality		
	HR	95% CI		HR	95% CI		HR	95% CI	
**Number of siblings (ref. No sibling)**									
One sibling	0.99	0.99	1.00	0.99	0.97	1.02	0.94	0.93	0.95
Two siblings	1.00	0.99	1.01	1.03	1.00	1.06	0.92	0.91	0.94
Three siblings	1.01	1.00	1.02	1.07	1.04	1.11	0.93	0.91	0.95
Four or more siblings	1.03	1.01	1.04	1.17	1.13	1.21	0.95	0.93	0.96
**Birth order (ref. First birth)**									
Second	1.01	1.01	1.02	1.07	1.05	1.09	0.96	0.95	0.98
Third	1.03	1.02	1.04	1.14	1.11	1.18	0.98	0.96	1.00
Fourth	1.04	1.02	1.05	1.14	1.09	1.20	1.01	0.98	1.04
Fifth+	1.04	1.02	1.06	1.22	1.15	1.29	1.03	0.99	1.07

*: Full adjusted model.

For peer review only

Figure 1. Hazard ratios of CHD by number of siblings and birth order in men (a,b) and women (c,d)

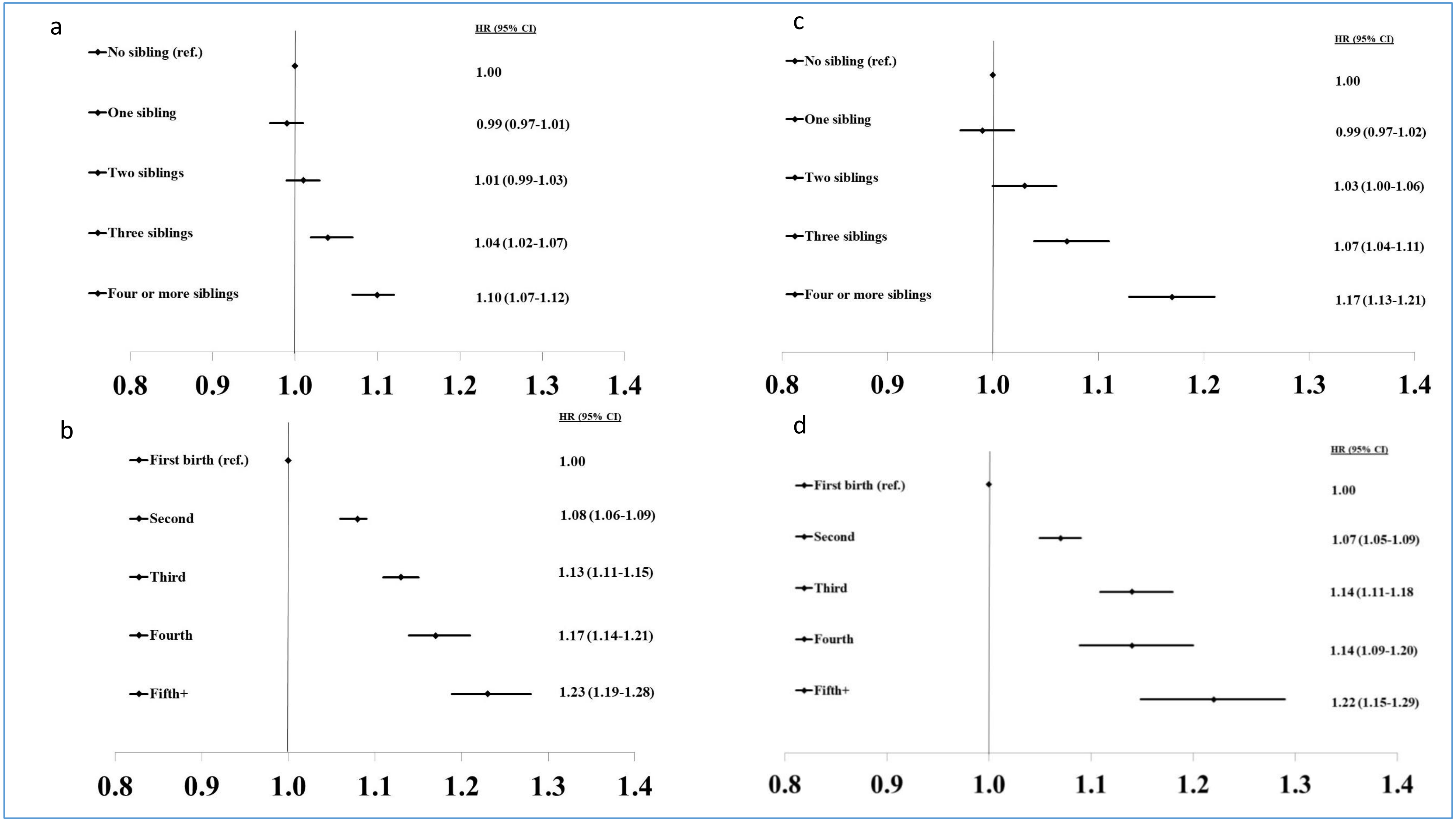

For peer review only - http://bmjopen.bmj.com/site/about/guidelines.xhtml

Figure 2. Hazard ratios of CVD by number of siblings and birth order in men (a,b) and women (c,d)

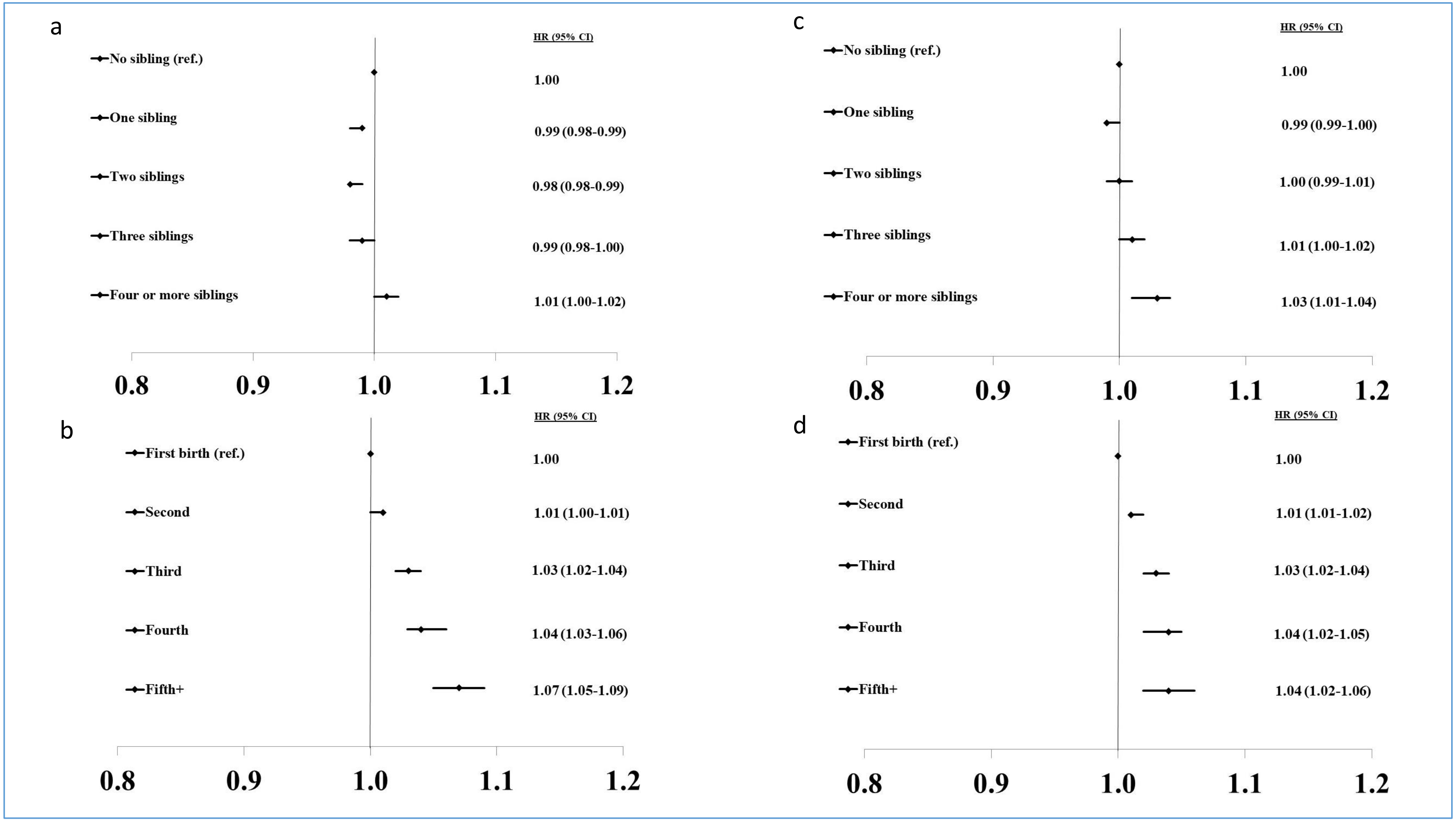

Figure 3. Hazard ratios of mortality by number of siblings and birth order in men (a,b) and women (c,d)

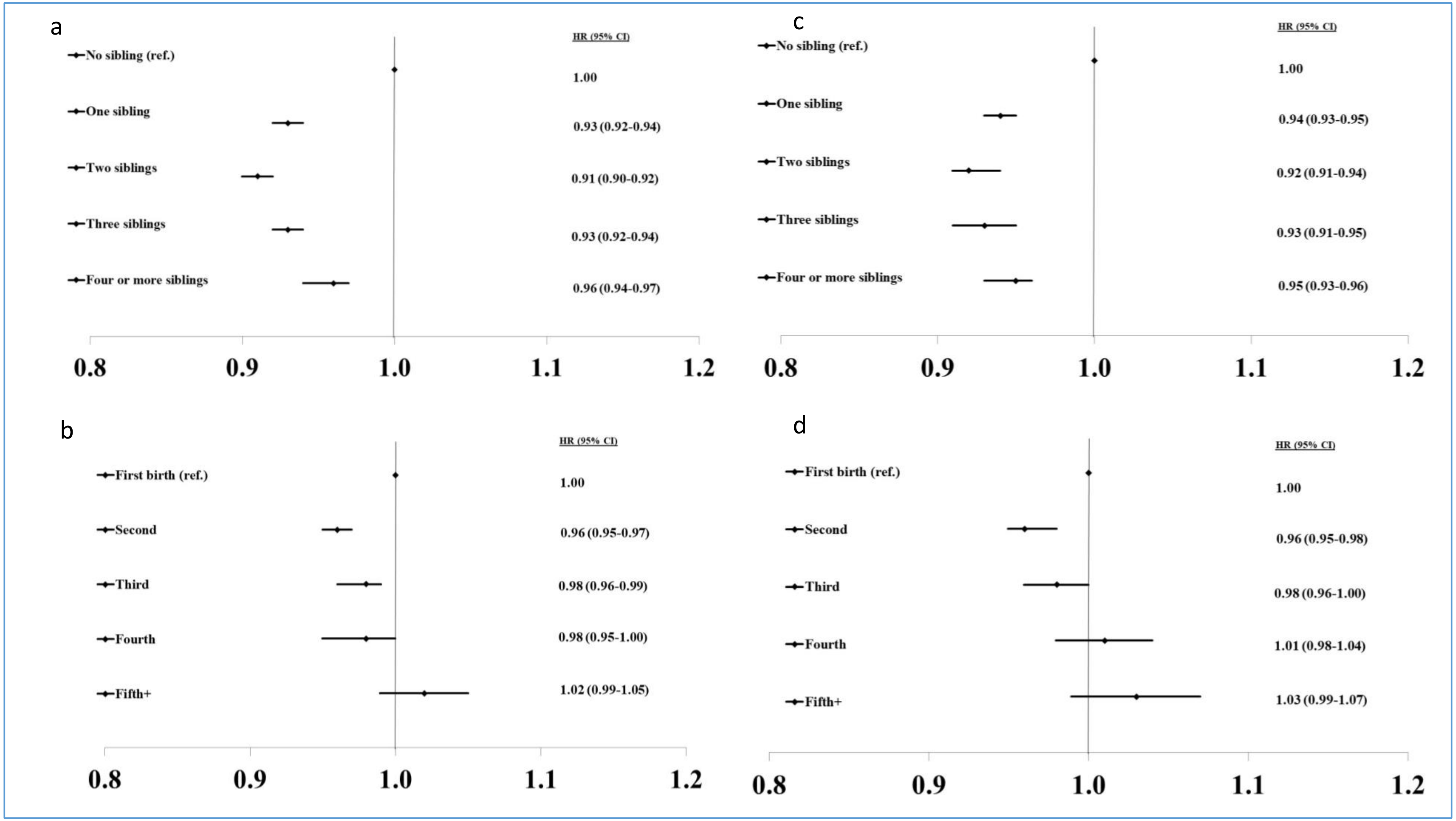

Supplementary Table 1a. Distribution of population, number of CVD, CHD, and mortality events in men.

	Population		CVD events		CHD events		Mortality events	
	No.	(%)	No.	%	No.	%	No.	%
Total population (%)	1358647		592863		131533		240371	
Age (years)								
30-39	531559	39.1	152615	25.7	25327	19.3	39094	16.3
40-49	554137	40.8	265251	44.7	59088	44.9	96549	40.2
50-58	272951	20.1	174997	29.5	47118	35.8	104728	43.6
Family income								
Low income	334083	24.6	123575	20.8	26649	20.3	47582	19.8
Middle–low income	320284	23.6	135148	22.8	29613	22.5	51243	21.3
Middle–high income	321653	23.7	152589	25.7	35041	26.6	66400	27.6
High income	382627	28.2	181551	30.6	40230	30.6	75146	31.3
Marital status								
Married/cohabiting	809314	59.6	370633	62.5	85212	64.8	124039	51.6
Never married, Widowed, or divorced	549333	40.4	222230	37.5	46321	35.2	116332	48.4
Educational attainment								
≤ 9 years	281207	20.7	146179	24.7	38890	29.6	81428	33.9
10–11 years	173208	12.7	67512	11.4	13955	10.6	26218	10.9
≥ 12 years	904232	66.6	379172	64.0	78688	59.8	132725	55.2
Immigrant status								
Sweden	1322658	97.4	580707	97.9	128732	97.9	235464	98.0
Other countries	35989	2.6	12156	2.1	2801	2.1	4907	2.0
Socioeconomic status								
Farmers/self-employed/others	308729	22.7	129408	21.8	29064	22.1	71960	29.9
Blue collar workers	494090	36.4	217215	36.6	50626	38.5	89989	37.4
White collar workers	351621	25.9	157170	26.5	34594	26.3	52582	21.9
Professionals	204207	15.0	89070	15.0	17249	13.1	25840	10.8
Urban/rural status								
Large cities	495681	36.5	207593	35.0	41153	31.3	85765	35.7
Southern Sweden	618686	45.5	274100	46.2	61581	46.8	108663	45.2
Northern Sweden	244280	18.0	111170	18.8	28799	21.9	45943	19.1
Hospitalization of alcoholism and related liver disease								
No	1277104	94.0	549201	92.6	124099	94.3	204523	85.1
Yes	81543	6.0	43662	7.4	7434	5.7	35848	14.9
Hospitalization of diabetes								
No	1227427	90.3	494421	83.4	104840	79.7	201011	83.6
Yes	131220	9.7	98442	16.6	26693	20.3	39360	16.4
Hospitalization of hypertension								
No	1059935	78.0	294151	49.6	76609	58.2	183958	76.5
Yes	298712	22.0	298712	50.4	54924	41.8	56413	23.5

For peer review only

For peer review only - http://bmjopen.bmj.com/site/about/guidelines.xhtml

Hospitalization of obesity								
No	1338892	98.5	577543	97.4	128595	97.8	236644	98.4
Yes	19755	1.5	15320	2.6	2938	2.2	3727	1.6
Hospitalization of chronic lower respiratory disease								
No	1288108	94.8	546393	92.2	120575	91.7	216522	90.1
Yes	70539	5.2	46470	7.8	10958	8.3	23849	9.9
Cancer								
No	1109571	81.7	452446	76.3	103202	78.5	139486	58.0
Yes	249076	18.3	140417	23.7	28331	21.5	100885	42.0
Number of sibling								
Non sibling	214700	15.8	105516	17.8	23671	18.0	50709	21.1
One sibling	443877	32.7	189839	32.0	39729	30.2	73140	30.4
Two siblings	338812	24.9	140361	23.7	30184	22.9	52790	22.0
Three siblings	183067	13.5	77378	13.1	17663	13.4	30266	12.6
Four or more siblings	178191	13.1	79769	13.5	20286	15.4	33466	13.9
Birth order								
First	684765	50.4	318341	53.7	70238	53.4	140857	58.6
Second	402879	29.7	166757	28.1	36654	27.9	62267	25.9
Third	164540	12.1	65853	11.1	14736	11.2	23081	9.6
Fourth	62765	4.6	24729	4.2	5737	4.4	8425	3.5
Fifth+	43698	3.2	17183	2.9	4168	3.2	5741	2.4

For peer review only

Supplementary Table 1b. Distribution of population, number of CVD, CHD, and mortality events in women.

	Population		CVD events		CHD events		Mortality events	
	No.	(%)	No.	%	No.	%	No.	%
Total population (%)	1315037		486147		55933		160269	
Age (years)								
30-39	507872	38.6	125326	25.8	9681	17.3	24571	15.3
40-49	537035	40.8	210113	43.2	23738	42.4	64055	40.0
50+	270130	20.5	150708	31.0	22514	40.3	71643	44.7
Family income								
Low income	343296	26.1	100542	20.7	10467	18.7	29723	18.5
Middle–low income	340837	25.9	121716	25.0	13432	24.0	37773	23.6
Middle–high income	347955	26.5	142924	29.4	17579	31.4	50537	31.5
High income	282949	21.5	120965	24.9	14455	25.8	42236	26.4
Marital status								
Married/cohabiting	887258	67.5	331886	68.3	38106	68.1	96533	60.2
Never married, Widowed, or divorced	427779	32.5	154261	31.7	17827	31.9	63736	39.8
Educational attainment								
≤ 9 years	232824	17.7	102037	21.0	15415	27.6	48455	30.2
10–11 years	165367	12.6	60209	12.4	6867	12.3	20489	12.8
≥ 12 years	916846	69.7	323901	66.6	33651	60.2	91325	57.0
Immigrant status								
Sweden	1281472	97.4	476075	97.9	54756	97.9	157515	98.3
Other countries	33565	2.6	10072	2.1	1177	2.1	2754	1.7
Socioeconomic status								
Farmers/self-employed/others	272189	20.7	96279	19.8	12450	22.3	45553	28.4
Blue collar workers	437324	33.3	174549	35.9	21821	39.0	55960	34.9
White collar workers	496782	37.8	178249	36.7	18324	32.8	49169	30.7
Professionals	108742	8.3	37070	7.6	3338	6.0	9587	6.0
Urban/rural status								
Large cities	495097	37.6	173872	35.8	17499	31.3	57794	36.1
Southern Sweden	591636	45.0	223375	45.9	25965	46.4	72872	45.5
Northern Sweden	228304	17.4	88900	18.3	12469	22.3	29603	18.5
Hospitalization of alcoholism and related liver disease								
No	1284048	97.6	471211	96.9	54423	97.3	149699	93.4
Yes	30989	2.4	14936	3.1	1510	2.7	10570	6.6
Hospitalization of diabetes								
No	1234668	93.9	429682	88.4	45572	81.5	139951	87.3
Yes	80369	6.1	56465	11.6	10361	18.5	20318	12.7
Hospitalization of hypertension								
No	1067928	81.2	239038	49.2	31364	56.1	124898	77.9
Yes	247109	18.8	247109	50.8	24569	43.9	35371	22.1
Hospitalization of obesity								

For peer review only

For peer review only - http://bmjopen.bmj.com/site/about/guidelines.xhtml

No	1286838	97.9	468228	96.3	54003	96.5	156771	97.8
Yes	28199	2.1	17919	3.7	1930	3.5	3498	2.2
Hospitalization of chronic lower respiratory disease								
No	1226846	93.3	433906	89.3	48084	86.0	137028	85.5
Yes	88191	6.7	52241	10.7	7849	14.0	23241	14.5
Cancer								
No	1051617	80.0	364226	74.9	43617	78.0	70300	43.9
Yes	263420	20.0	121921	25.1	12316	22.0	89969	56.1
Number of sibling								
Non sibling	210121	16.0	87261	17.9	10289	18.4	34521	21.5
One sibling	430315	32.7	154154	31.7	16280	29.1	49132	30.7
Two siblings	324379	24.7	113739	23.4	12500	22.3	34843	21.7
Three siblings	176631	13.4	63871	13.1	7512	13.4	19766	12.3
Four or more siblings	173591	13.2	67122	13.8	9352	16.7	22007	13.7
Birth order								
First	664459	50.5	262015	53.9	30342	54.2	94779	59.1
Second	388391	29.5	136263	28.0	15191	27.2	40984	25.6
Third	159311	12.1	53711	11.0	6231	11.1	15205	9.5
Fourth	60676	4.6	20264	4.2	2379	4.3	5558	3.5
Fifth+	42200	3.2	13894	2.9	1790	3.2	3743	2.3

For peer review only

Supplementary Table 2a. Hazard ratio (HR) and 95% confidence interval of CVD in men.

	Model 1			Model 2			Model 3		
	HR	95% CI		HR	95% CI		HR	95% CI	
Number of siblings (ref. No sibling)									
One sibling	0.98	0.98	0.99	0.98	0.98	0.99	0.99	0.98	0.99
Two siblings	0.98	0.97	0.99	0.97	0.96	0.98	0.98	0.98	0.99
Three siblings	1.00	0.99	1.01	0.98	0.97	0.99	0.99	0.98	1.00
Four or more siblings	1.04	1.03	1.05	1.01	1.00	1.02	1.01	1.00	1.02
Age (years)	1.07	1.07	1.07	1.07	1.07	1.07	1.05	1.05	1.05
Family income (ref. High)									
Low income				0.89	0.88	0.90	0.96	0.95	0.97
Middle–low income				0.98	0.97	0.99	1.01	1.01	1.02
Middle–high income				1.01	1.00	1.02	1.02	1.01	1.03
Educational level (ref. ≤ 9 years)									
10–11 years				1.11	1.10	1.12	1.07	1.06	1.08
≥ 12 years				1.02	1.02	1.03	1.02	1.01	1.03
Socioeconomic status (ref. professionals)									
Farmers/self-employed/others				1.14	1.13	1.15	1.09	1.08	1.10
Blue collar workers				1.12	1.11	1.13	1.06	1.05	1.07
White collar workers				1.07	1.06	1.08	1.03	1.02	1.04
Region of residence (ref. Large cities)									
Southern Sweden				1.02	1.01	1.02	1.03	1.03	1.04
Northern Sweden				1.05	1.04	1.06	1.04	1.03	1.05
Immigrant status (ref. Born in Sweden)				0.94	0.92	0.95	0.96	0.94	0.97
Marital status (ref. Not married)				1.06	1.06	1.07	1.07	1.06	1.07
Birth order				1.01	1.01	1.01	1.01	1.01	1.02
Hospitalization of chronic lower respiratory disease (ref. Non)							1.34	1.33	1.35
Hospitalization of alcoholism and related liver disease (ref. Non)							1.46	1.45	1.48
Hospitalization of diabetes (ref. Non)							1.45	1.44	1.46
Hospitalization of obesity (ref. Non)							1.40	1.38	1.42
Hospitalization of hypertension (ref. Non)							5.19	5.17	5.22
Cancer (ref. Non)							1.16	1.15	1.16

Model 1. Adjusted for age at start; Model 2. Adjusted for age at start and individual characteristics; Model 3. Model 2 + comorbidities.

Supplementary Table 2b. Hazard ratio (HR) and 95% confidence interval of CVD in men.

	Model 1		Model 2		Model 3	
	HR	95% CI	HR	95% CI	HR	95% CI
Birth order (ref. First birth)						

For peer review only

	Model 1 HR	95% CI		Model 2 HR	95% CI		Model 3 HR	95% CI	
Second	0.99	0.99	1.00	0.99	0.98	0.99	1.01	1.00	1.01
Third	1.02	1.01	1.03	1.01	1.00	1.02	1.03	1.02	1.04
Fourth	1.04	1.03	1.05	1.02	1.01	1.04	1.04	1.03	1.06
Fifth+	1.09	1.07	1.10	1.06	1.04	1.08	1.07	1.05	1.09
Age (years)	1.07	1.07	1.07	1.07	1.07	1.07	1.05	1.05	1.05
Family income (ref. High)									
Low income				0.89	0.88	0.90	0.96	0.95	0.97
Middle–low income				0.98	0.97	0.99	1.01	1.01	1.02
Middle–high income				1.01	1.00	1.02	1.02	1.01	1.03
Educational level (ref. ≤ 9 years)									
10–11 years				1.11	1.10	1.12	1.07	1.06	1.08
≥ 12 years				1.02	1.02	1.03	1.02	1.01	1.03
Socioeconomic status (ref. professionals)									
Farmers/self-employed/others				1.14	1.13	1.15	1.09	1.08	1.10
Blue collar workers				1.12	1.11	1.13	1.06	1.05	1.07
White collar workers				1.07	1.06	1.08	1.03	1.02	1.04
Region of residence (ref. Large cities)									
Southern Sweden				1.02	1.01	1.02	1.03	1.03	1.04
Northern Sweden				1.05	1.04	1.06	1.04	1.03	1.05
Immigrant status (ref. Born in Sweden)				0.94	0.92	0.96	0.96	0.94	0.97
Marital status (ref. Not married)				1.06	1.06	1.07	1.07	1.06	1.07
Number of siblings				1.00	1.00	1.00	1.00	1.00	1.00
Hospitalization of chronic lower respiratory disease (ref. Non)							1.34	1.33	1.35
Hospitalization of alcoholism and related liver disease (ref. Non)							1.46	1.45	1.48
Hospitalization of diabetes (ref. Non)							1.45	1.44	1.46
Hospitalization of obesity (ref. Non)							1.40	1.38	1.42
Hospitalization of hypertension (ref. Non)							5.19	5.17	5.22
Cancer (ref. Non)							1.16	1.15	1.16

Model 1. Adjusted for age at start; Model 2. Adjusted for age at start and individual characteristics; Model 3. Model 2 + comorbidities.

For peer review only

Supplementary Table 2c. Hazard ratio (HR) and 95% confidence interval of CHD in men.

	Model 1			Model 2			Model 3		
	HR	95% CI		HR	95% CI		HR	95% CI	
Number of siblings (ref. No sibling)									
One sibling	1.00	0.98	1.02	0.99	0.97	1.01	0.99	0.97	1.01
Two siblings	1.04	1.03	1.06	1.00	0.98	1.02	1.01	0.99	1.03
Three siblings	1.12	1.10	1.14	1.04	1.02	1.06	1.04	1.02	1.07
Four or more siblings	1.27	1.25	1.30	1.10	1.08	1.12	1.10	1.07	1.12
Age (years)	1.08	1.08	1.08	1.08	1.08	1.08	1.07	1.07	1.07

For peer review only - http://bmjopen.bmj.com/site/about/guidelines.xhtml

Family income (ref. High)						
Low income	0.93	0.91	0.94	0.96	0.95	0.98
Middle–low income	1.00	0.98	1.01	1.02	1.00	1.03
Middle–high income	1.01	1.00	1.03	1.02	1.00	1.03
Educational level (ref. ≤ 9 years)						
10–11 years	1.05	1.03	1.07	1.04	1.02	1.07
≥ 12 years	0.94	0.93	0.95	0.95	0.94	0.96
Socioeconomic status (ref. professionals)						
Farmers/self-employed/others	1.23	1.20	1.25	1.18	1.16	1.21
Blue collar workers	1.26	1.24	1.28	1.21	1.19	1.23
White collar workers	1.18	1.16	1.20	1.15	1.13	1.17
Region of residence (ref. Large cities)						
Southern Sweden	1.11	1.10	1.12	1.12	1.11	1.14
Northern Sweden	1.32	1.30	1.34	1.31	1.29	1.33
Immigrant status (ref. Born in Sweden)	1.09	1.05	1.13	1.10	1.06	1.14
Marital status (ref. Not married)	1.00	0.99	1.01	0.99	0.98	1.01
Birth order	1.05	1.04	1.05	1.05	1.05	1.06
Hospitalization of chronic lower respiratory disease (ref. Non)				1.29	1.26	1.31
Hospitalization of alcoholism and related liver disease (ref. Non)				1.02	1.00	1.05
Hospitalization of diabetes (ref. Non)				1.75	1.72	1.77
Hospitalization of obesity (ref. Non)				1.13	1.09	1.17
Hospitalization of hypertension (ref. Non)				1.86	1.84	1.88
Cancer (ref. Non)				0.91	0.89	0.92

Model 1. Adjusted for age at start; Model 2. Adjusted for age at start and individual characteristics; Model 3. Model 2 + comorbidities.

For peer review only

Supplementary Table 2d. Hazard ratio (HR) and 95% confidence interval of CHD in men.

	Model 1			Model 2			Model 3		
	HR	95% CI		HR	95% CI		HR	95% CI	
Birth order (ref. First birth)									
Second	1.09	1.08	1.10	1.06	1.05	1.07	1.08	1.06	1.09
Third	1.19	1.17	1.21	1.11	1.09	1.13	1.13	1.11	1.15
Fourth	1.28	1.25	1.32	1.15	1.12	1.19	1.17	1.14	1.21
Fifth+	1.44	1.39	1.48	1.22	1.17	1.26	1.23	1.19	1.28
Age (years)	1.08	1.08	1.09	1.08	1.08	1.08	1.07	1.07	1.07
Family income (ref. High)									
Low income				0.93	0.91	0.94	0.96	0.95	0.98
Middle–low income				1.00	0.98	1.01	1.02	1.00	1.03
Middle–high income				1.01	1.00	1.02	1.02	1.00	1.03
Educational level (ref. ≤ 9 years)									
10–11 years				1.05	1.03	1.08	1.05	1.03	1.07
≥ 12 years				0.94	0.93	0.95	0.95	0.94	0.96
Socioeconomic status (ref. professionals)									
Farmers/self-employed/others				1.23	1.20	1.25	1.18	1.16	1.21
Blue collar workers				1.26	1.24	1.28	1.21	1.19	1.23
White collar workers				1.18	1.16	1.20	1.15	1.13	1.17
Region of residence (ref. Large cities)									
Southern Sweden				1.11	1.10	1.13	1.12	1.11	1.14
Northern Sweden				1.32	1.30	1.34	1.31	1.29	1.33
Immigrant status (ref. Born in Sweden)				1.09	1.05	1.13	1.10	1.06	1.14
Marital status (ref. Not married)				1.00	0.99	1.01	0.99	0.98	1.01
Number of siblings				1.02	1.02	1.02	1.02	1.02	1.02
Hospitalization of chronic lower respiratory disease (ref. Non)							1.29	1.26	1.31
Hospitalization of alcoholism and related liver disease (ref. Non)							1.02	1.00	1.05
Hospitalization of diabetes (ref. Non)							1.75	1.72	1.77
Hospitalization of obesity (ref. Non)							1.13	1.09	1.17
Hospitalization of hypertension (ref. Non)							1.86	1.84	1.88
Cancer (ref. Non)							0.91	0.89	0.92

Model 1. Adjusted for age at start; Model 2. Adjusted for age at start and individual characteristics; Model 3. Model 2 + comorbidities.

For peer review only

Supplementary Table 2e. Hazard ratio (HR) and 95% confidence interval of mortality in men.

	Model 1			Model 2			Model 3		
	HR	95% CI		HR	95% CI		HR	95% CI	
Number of siblings (ref. No sibling)									
One sibling	0.91	0.90	0.92	0.92	0.91	0.93	0.93	0.92	0.94
Two siblings	0.92	0.91	0.93	0.91	0.90	0.92	0.91	0.90	0.92
Three siblings	0.96	0.94	0.97	0.93	0.92	0.94	0.93	0.92	0.94
Four or more siblings	1.03	1.01	1.04	0.96	0.94	0.97	0.96	0.94	0.97
Age (years)	1.10	1.10	1.10	1.11	1.11	1.11	1.10	1.10	1.10
Family income (ref. High)									
Low income				1.05	1.04	1.07	1.08	1.07	1.10
Middle–low income				1.17	1.15	1.18	1.14	1.13	1.16
Middle–high income				1.18	1.17	1.19	1.15	1.14	1.16
Educational level (ref. ≤ 9 years)									
10–11 years				1.12	1.10	1.13	1.08	1.06	1.09
≥ 12 years				0.91	0.90	0.91	0.89	0.88	0.90
Socioeconomic status (ref. professionals)									
Farmers/self-employed/others				1.73	1.71	1.76	1.64	1.61	1.66
Blue collar workers				1.38	1.36	1.40	1.35	1.33	1.37
White collar workers				1.15	1.13	1.17	1.14	1.13	1.16
Region of residence (ref. Large cities)									
Southern Sweden				0.94	0.93	0.95	0.99	0.98	1.00
Northern Sweden				0.98	0.97	0.99	1.08	1.07	1.09
Immigrant status (ref. Born in Sweden)				1.04	1.01	1.07	1.06	1.03	1.09
Marital status (ref. Not married)				1.88	1.87	1.90	1.73	1.71	1.74
Birth order				1.00	1.00	1.01	0.99	0.99	1.00
Hospitalization of chronic lower respiratory disease (ref. Non)							1.29	1.27	1.31
Hospitalization of alcoholism and related liver disease (ref. Non)							2.87	2.84	2.90
Hospitalization of diabetes (ref. Non)							1.40	1.39	1.42
Hospitalization of obesity (ref. Non)							1.10	1.06	1.14
Hospitalization of hypertension (ref. Non)							0.66	0.66	0.67
Cancer (ref. Non)							2.62	2.60	2.64

Model 1. Adjusted for age at start; Model 2. Adjusted for age at start and individual characteristics; Model 3. Model 2 + comorbidities.

Supplementary Table 2f. Hazard ratio (HR) and 95% confidence interval of mortality in men.

	Model 1		Model 2		Model 3	
	HR	95% CI	HR	95% CI	HR	95% CI
Birth order (ref. First birth)						

For peer review only

For peer review only - http://bmjopen.bmj.com/site/about/guidelines.xhtml

Second	0.98	0.97	0.99	0.97	0.96	0.98	0.96	0.95	0.97
Third	1.01	0.99	1.02	0.99	0.98	1.01	0.98	0.96	0.99
Fourth	1.04	1.01	1.06	1.00	0.98	1.03	0.98	0.95	1.00
Fifth+	1.11	1.08	1.14	1.06	1.02	1.09	1.02	0.99	1.05
Age (years)	1.10	1.10	1.10	1.11	1.11	1.11	1.10	1.10	1.10
Family income (ref. High)									
Low income				1.06	1.04	1.07	1.08	1.07	1.10
Middle–low income				1.17	1.15	1.18	1.14	1.13	1.16
Middle–high income				1.18	1.17	1.19	1.15	1.14	1.16
Educational level (ref≤ 9 years)									
10–11 years				1.12	1.10	1.13	1.08	1.06	1.09
≥ 12 years				0.90	0.90	0.91	0.89	0.88	0.90
Socioeconomic status (ref. professionals)									
Farmers/self-employed/others				1.73	1.71	1.76	1.64	1.61	1.66
Blue collar workers				1.38	1.36	1.40	1.35	1.33	1.37
White collar workers				1.15	1.13	1.17	1.14	1.13	1.16
Region of residence (ref. Large cities)									
Southern Sweden				0.94	0.93	0.95	0.99	0.98	1.00
Northern Sweden				0.98	0.97	0.99	1.08	1.07	1.09
Immigrant status (ref. Born in Sweden)				1.05	1.02	1.08	1.06	1.03	1.09
Marital status (ref. Not married)				1.88	1.87	1.90	1.73	1.71	1.74
Number of siblings				1.00	0.99	1.00	1.00	0.99	1.00
Hospitalization of chronic lower respiratory disease (ref. Non)							1.29	1.27	1.31
Hospitalization of alcoholism and related liver disease (ref. Non)							2.87	2.84	2.90
Hospitalization of diabetes (ref. Non)							1.40	1.39	1.42
Hospitalization of obesity (ref. Non)							1.10	1.06	1.14
Hospitalization of hypertension (ref. Non)							0.66	0.66	0.67
Cancer (ref. Non)							2.62	2.60	2.64

Model 1. Adjusted for age at start; Model 2. Adjusted for age at start and individual characteristics; Model 3. Model 2 + comorbidities.

Supplementary Table 3a. Hazard ratio (HR) and 95% confidence interval of CVD in women.

	Model 1			Model 2			Model 3		
	HR	95% CI		HR	95% CI		HR	95% CI	
Number of siblings (ref. No sibling)									
One sibling	0.99	0.98	0.99	0.99	0.98	0.99	0.99	0.99	1.00
Two siblings	1.00	0.99	1.00	0.99	0.98	1.00	1.00	0.99	1.01

For peer review only

	HR	95% CI		HR	95% CI		HR	95% CI	
Three siblings	1.02	1.01	1.03	1.01	1.00	1.02	1.01	1.00	1.02
Four or more siblings	1.07	1.06	1.08	1.04	1.03	1.06	1.03	1.01	1.04
Age (years)	1.06	1.06	1.06	1.06	1.06	1.06	1.03	1.03	1.04
Family income (ref. High)									
Low income				0.87	0.86	0.87	0.95	0.94	0.96
Middle–low income				1.01	1.00	1.02	1.03	1.02	1.04
Middle–high income				1.02	1.01	1.02	1.03	1.02	1.03
Educational level (ref. ≤ 9 years)									
10–11 years				1.13	1.12	1.15	1.08	1.06	1.09
≥ 12 years				1.06	1.05	1.07	1.05	1.05	1.06
Socioeconomic status (ref. professionals)									
Farmers/self-employed/others				1.22	1.21	1.24	1.12	1.11	1.14
Blue collar workers				1.25	1.24	1.27	1.09	1.08	1.10
White collar workers				1.09	1.07	1.10	1.03	1.01	1.04
Region of residence (ref. Large cities)									
Southern Sweden				1.03	1.03	1.04	1.03	1.03	1.04
Northern Sweden				1.07	1.06	1.08	1.03	1.02	1.04
Immigrant status (ref. Born in Sweden)				0.99	0.97	1.01	0.99	0.97	1.01
Marital status (ref. Not married)				1.07	1.06	1.07	1.04	1.04	1.05
Birth order				1.00	1.00	1.00	1.01	1.01	1.01
Hospitalization of chronic lower respiratory disease (ref. Non)							1.41	1.39	1.42
Hospitalization of alcoholism and related liver disease (ref. Non)							1.40	1.38	1.43
Hospitalization of diabetes (ref. Non)							1.42	1.40	1.43
Hospitalization of obesity (ref. Non)							1.34	1.32	1.37
Hospitalization of hypertension (ref. Non)							6.73	6.69	6.77
Cancer (ref. Non)							1.25	1.25	1.26

Model 1. Adjusted for age at start; Model 2. Adjusted for age at start and individual characteristics; Model 3. Model 2 + comorbidities.

Supplementary Table 3b. Hazard ratio (HR) and 95% confidence interval of CVD in women.

	Model 1			Model 2			Model 3		
	HR	95% CI		HR	95% CI		HR	95% CI	
Birth order (ref. First birth)									
Second	1.00	0.99	1.01	0.99	0.98	0.99	1.01	1.01	1.02
Third	1.02	1.02	1.03	0.99	0.98	1.00	1.03	1.02	1.04
Fourth	1.05	1.04	1.07	1.00	0.99	1.02	1.04	1.02	1.05
Fifth+	1.08	1.06	1.10	1.00	0.98	1.02	1.04	1.02	1.06
Age (years)	1.06	1.06	1.06	1.06	1.06	1.06	1.03	1.03	1.04
Family income (ref. High)									
Low income				0.87	0.86	0.87	0.95	0.94	0.96

For peer review only - http://bmjopen.bmj.com/site/about/guidelines.xhtml

Middle–low income	1.01	1.00	1.02	1.03	1.02	1.04
Middle–high income	1.02	1.01	1.02	1.03	1.02	1.03
Educational level (ref. ≤ 9 years)						
10–11 years	1.13	1.12	1.15	1.08	1.06	1.09
≥ 12 years	1.06	1.05	1.07	1.05	1.05	1.06
Socioeconomic status (ref. professionals)						
Farmers/self-employed/others	1.22	1.21	1.24	1.12	1.11	1.14
Blue collar workers	1.25	1.24	1.27	1.09	1.08	1.10
White collar workers	1.09	1.08	1.10	1.03	1.01	1.04
Region of residence (ref. Large cities)						
Southern Sweden	1.03	1.03	1.04	1.03	1.03	1.04
Northern Sweden	1.07	1.06	1.08	1.03	1.02	1.04
Immigrant status (ref. Born in Sweden)	0.99	0.97	1.01	0.99	0.97	1.01
Marital status (ref. Not married)	1.07	1.06	1.07	1.04	1.04	1.05
Number of siblings	1.01	1.01	1.01	1.01	1.00	1.01
Hospitalization of chronic lower respiratory disease (ref. Non)				1.41	1.39	1.42
Hospitalization of alcoholism and related liver disease (ref. Non)				1.40	1.38	1.43
Hospitalization of diabetes (ref. Non)				1.42	1.41	1.43
Hospitalization of obesity (ref. Non)				1.34	1.32	1.37
Hospitalization of hypertension (ref. Non)				6.73	6.69	6.77
Cancer (ref. Non)				1.25	1.25	1.26

Model 1. Adjusted for age at start; Model 2. Adjusted for age at start and individual characteristics; Model 3. Model 2 + comorbidities.

Supplementary Table 3c. Hazard ratio (HR) and 95% confidence interval of CHD in women.

	Model 1			Model 2			Model 3		
	HR	95% CI		HR	95% CI		HR	95% CI	
Number of siblings (ref. No sibling)									
One sibling	0.99	0.97	1.02	0.98	0.96	1.01	0.99	0.97	1.02
Two siblings	1.07	1.04	1.10	1.03	1.00	1.05	1.03	1.00	1.06
Three siblings	1.17	1.13	1.20	1.08	1.04	1.11	1.07	1.04	1.11
Four or more siblings	1.40	1.36	1.44	1.20	1.16	1.24	1.17	1.13	1.21
Age (years)	1.09	1.09	1.09	1.09	1.08	1.09	1.07	1.07	1.07
Family income (ref. High)									
Low income				0.85	0.83	0.88	0.91	0.88	0.93
Middle–low income				1.00	0.98	1.03	1.01	0.98	1.03
Middle–high income				1.02	1.00	1.05	1.02	0.99	1.04
Educational level (ref. ≤ 9 years)									
10–11 years				1.07	1.04	1.10	1.06	1.03	1.09
≥ 12 years				0.92	0.90	0.94	0.94	0.92	0.96

For peer review only

For peer review only - http://bmjopen.bmj.com/site/about/guidelines.xhtml

Socioeconomic status (ref. professionals)						
Farmers/self-employed/others	1.52	1.46	1.59	1.37	1.31	1.42
Blue collar workers	1.47	1.42	1.53	1.33	1.28	1.38
White collar workers	1.19	1.14	1.23	1.14	1.10	1.18
Region of residence (ref. Large cities)						
Southern Sweden	1.14	1.12	1.16	1.15	1.13	1.17
Northern Sweden	1.40	1.37	1.44	1.38	1.34	1.41
Immigrant status (ref. Born in Sweden)	1.13	1.07	1.20	1.13	1.07	1.20
Marital status (ref. Not married)	1.12	1.10	1.14	1.08	1.06	1.10
Birth order	1.04	1.03	1.05	1.05	1.04	1.06
Hospitalization of chronic lower respiratory disease (ref. Non)				1.69	1.65	1.73
Hospitalization of alcoholism and related liver disease (ref. Non)				1.17	1.11	1.23
Hospitalization of diabetes (ref. Non)				2.19	2.14	2.24
Hospitalization of obesity (ref. Non)				1.11	1.06	1.17
Hospitalization of hypertension (ref. Non)				2.22	2.18	2.26
Cancer (ref. Non)				0.94	0.93	0.96

Model 1. Adjusted for age at start; Model 2. Adjusted for age at start and individual characteristics; Model 3. Model 2 + comorbidities.

Supplementary Table 3d. Hazard ratio (HR) and 95% confidence interval of CHD in women.

	Model 1			Model 2			Model 3		
	HR	95% CI		HR	95% CI		HR	95% CI	
Birth order (ref. First birth)									
Second	1.10	1.07	1.12	1.05	1.02	1.07	1.07	1.05	1.09
Third	1.24	1.21	1.28	1.11	1.08	1.14	1.14	1.11	1.18
Fourth	1.33	1.27	1.38	1.11	1.06	1.16	1.14	1.09	1.20
Fifth+	1.56	1.48	1.63	1.17	1.11	1.24	1.22	1.15	1.29
Age (years)	1.09	1.09	1.09	1.09	1.08	1.09	1.07	1.07	1.07
Family income (ref. High)									
Low income				0.85	0.83	0.88	0.91	0.88	0.93
Middle–low income				1.00	0.98	1.03	1.01	0.98	1.03
Middle–high income				1.02	1.00	1.05	1.02	0.99	1.04
Educational level (ref. ≤ 9 years)									
10–11 years				1.07	1.04	1.10	1.06	1.03	1.09
≥ 12 years				0.92	0.90	0.94	0.94	0.92	0.96
Socioeconomic status (ref. professionals)									
Farmers/self-employed/others				1.52	1.46	1.59	1.37	1.31	1.42
Blue collar workers				1.47	1.42	1.53	1.33	1.28	1.38
White collar workers				1.19	1.14	1.23	1.14	1.10	1.18
Region of residence (ref. Large cities)									

For peer review only

Southern Sweden	1.14	1.12	1.17	1.15	1.13	1.17
Northern Sweden	1.40	1.37	1.44	1.38	1.35	1.41
Immigrant status (ref. Born in Sweden)	1.13	1.07	1.20	1.13	1.07	1.20
Marital status (ref. Not married)	1.12	1.10	1.14	1.08	1.06	1.10
Number of siblings	1.04	1.03	1.04	1.03	1.03	1.04
Hospitalization of chronic lower respiratory disease (ref. Non)				1.69	1.65	1.73
Hospitalization of alcoholism and related liver disease (ref. Non)				1.17	1.11	1.23
Hospitalization of diabetes (ref. Non)				2.19	2.15	2.24
Hospitalization of obesity (ref. Non)				1.12	1.06	1.17
Hospitalization of hypertension (ref. Non)				2.22	2.18	2.26
Cancer (ref. Non)				0.94	0.93	0.96

Model 1. Adjusted for age at start; Model 2. Adjusted for age at start and individual characteristics; Model 3. Model 2 + comorbidities.

Supplementary Table 3e. Hazard ratio (HR) and 95% confidence interval of mortality in women.

	Model 1			Model 2			Model 3		
	HR	95% CI		HR	95% CI		HR	95% CI	
Number of siblings (ref. No sibling)									
One sibling	0.92	0.91	0.94	0.93	0.92	0.95	0.94	0.93	0.95
Two siblings	0.92	0.91	0.94	0.91	0.90	0.93	0.92	0.91	0.94
Three siblings	0.95	0.93	0.96	0.92	0.90	0.93	0.93	0.91	0.95
Four or more siblings	1.01	0.99	1.03	0.94	0.92	0.95	0.95	0.93	0.96
Age (years)	1.10	1.10	1.10	1.10	1.10	1.10	1.09	1.08	1.09
Family income (ref. High)									
Low income				0.84	0.83	0.86	0.94	0.93	0.96
Middle–low income				1.03	1.01	1.05	1.06	1.04	1.07
Middle–high income				1.05	1.04	1.06	1.05	1.04	1.07
Educational level (ref. ≤ 9 years)									
10–11 years				1.08	1.06	1.10	1.01	1.00	1.03
≥ 12 years				0.85	0.84	0.87	0.82	0.81	0.83
Socioeconomic status (ref. professionals)									
Farmers/self-employed/others				1.97	1.93	2.02	1.89	1.84	1.93
Blue collar workers				1.36	1.33	1.39	1.35	1.32	1.38
White collar workers				1.12	1.10	1.15	1.11	1.09	1.14
Region of residence (ref. Large cities)									
Southern Sweden				1.00	0.99	1.01	1.03	1.01	1.04
Northern Sweden				1.03	1.02	1.05	1.11	1.09	1.12
Immigrant status (ref. Born in Sweden)				0.89	0.86	0.92	0.95	0.92	0.99
Marital status (ref. Not married)				1.63	1.62	1.65	1.49	1.47	1.50
Birth order				1.01	1.00	1.01	1.00	0.99	1.01

For peer review only

Hospitalization of chronic lower respiratory disease (ref. Non)	1.61	1.59	1.64
Hospitalization of alcoholism and related liver disease (ref. Non)	3.12	3.06	3.18
Hospitalization of diabetes (ref. Non)	1.56	1.54	1.58
Hospitalization of obesity (ref. Non)	0.96	0.93	0.99
Hospitalization of hypertension (ref. Non)	0.66	0.65	0.67
Cancer (ref. Non)	4.86	4.81	4.91

Model 1. Adjusted for age at start; Model 2. Adjusted for age at start and individual characteristics; Model 3. Model 2 + comorbidities.

For peer review only

Supplementary Table 3f. Hazard ratio (HR) and 95% confidence interval of mortality in women.

	Model 1			Model 2			Model 3		
	HR	95% CI		HR	95% CI		HR	95% CI	
Birth order (ref. First birth)									
Second	0.98	0.96	0.99	0.97	0.96	0.98	0.96	0.95	0.98
Third	1.01	1.00	1.03	1.00	0.98	1.02	0.98	0.96	1.00
Fourth	1.05	1.02	1.08	1.02	0.99	1.05	1.01	0.98	1.04
Fifth+	1.11	1.08	1.15	1.08	1.04	1.12	1.03	0.99	1.07
Age (years)	1.10	1.10	1.10	1.10	1.10	1.10	1.09	1.08	1.09
Family income (ref. High)									
Low income				0.84	0.83	0.86	0.94	0.93	0.96
Middle–low income				1.03	1.02	1.05	1.06	1.04	1.07
Middle–high income				1.05	1.04	1.07	1.05	1.04	1.07
Educational level (ref. ≤ 9 years)									
10–11 years				1.08	1.06	1.10	1.01	1.00	1.03
≥ 12 years				0.85	0.84	0.86	0.82	0.81	0.83
Socioeconomic status (ref. professionals)									
Farmers/self-employed/others				1.98	1.93	2.02	1.89	1.85	1.94
Blue collar workers				1.36	1.33	1.39	1.35	1.32	1.38
White collar workers				1.12	1.10	1.15	1.11	1.09	1.14
Region of residence (ref. Large cities)									
Southern Sweden				1.00	0.99	1.01	1.03	1.01	1.04
Northern Sweden				1.03	1.02	1.05	1.11	1.09	1.12
Immigrant status (ref. Born in Sweden)				0.89	0.86	0.92	0.95	0.92	0.99
Marital status (ref. Not married)				1.63	1.62	1.65	1.49	1.47	1.50
Number of siblings				0.99	0.99	0.99	0.99	0.99	1.00
Hospitalization of chronic lower respiratory disease (ref. Non)							1.61	1.59	1.64
Hospitalization of alcoholism and related liver disease (ref. Non)							3.12	3.06	3.18
Hospitalization of diabetes (ref. Non)							1.56	1.54	1.58
Hospitalization of obesity (ref. Non)							0.96	0.93	0.99
Hospitalization of hypertension (ref. Non)							0.66	0.65	0.67
Cancer (ref. Non)							4.86	4.81	4.91

Model 1. Adjusted for age at start; Model 2. Adjusted for age at start and individual characteristics; Model 3. Model 2 + comorbidities.

For peer review only

Supplemental Figure S1.

Flow-chart of the study sample identified in the Swedish Multi-Generation Register (MGR).

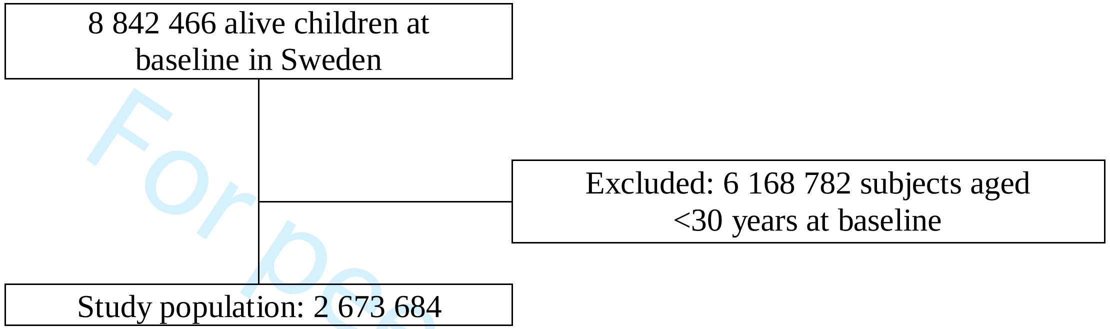

For peer review only

STROBE Statement—Checklist of items that should be included in reports of ***cohort studies***

	Item No	Recommendation	Page No
**Title and abstract**	1	(*a*) Indicate the study's design with a commonly used term in the title or the abstract	1
		(*b*) Provide in the abstract an informative and balanced summary of what was done and what was found	2
**Introduction**			
Background/rationale	2	Explain the scientific background and rationale for the investigation being reported	4
Objectives	3	State specific objectives, including any prespecified hypotheses	4
**Methods**			
Study design	4	Present key elements of study design early in the paper	4
Setting	5	Describe the setting, locations, and relevant dates, including periods of recruitment, exposure, follow-up, and data collection	5
Participants	6	(*a*) Give the eligibility criteria, and the sources and methods of selection of participants. Describe methods of follow-up<br>(*b*) For matched studies, give matching criteria and number of exposed and unexposed	4-5
Variables	7	Clearly define all outcomes, exposures, predictors, potential confounders, and effect modifiers. Give diagnostic criteria, if applicable	5
Data sources/ measurement	8*	For each variable of interest, give sources of data and details of methods of assessment (measurement). Describe comparability of assessment methods if there is more than one group	5
Bias	9	Describe any efforts to address potential sources of bias	8
Study size	10	Explain how the study size was arrived at	4-5
Quantitative variables	11	Explain how quantitative variables were handled in the analyses. If applicable, describe which groupings were chosen and why	5
Statistical methods	12	(*a*) Describe all statistical methods, including those used to control for confounding<br>(*b*) Describe any methods used to examine subgroups and interactions<br>(*c*) Explain how missing data were addressed<br>(*d*) If applicable, explain how loss to follow-up was addressed<br>(*e*) Describe any sensitivity analyses	6
**Results**			
Participants	13*	(a) Report numbers of individuals at each stage of study—eg numbers potentially eligible, examined for eligibility, confirmed eligible, included in the study, completing follow-up, and analysed<br>(b) Give reasons for non-participation at each stage<br>(c) Consider use of a flow diagram	6
Descriptive data	14*	(a) Give characteristics of study participants (eg demographic, clinical, social) and information on exposures and potential confounders<br>(b) Indicate number of participants with missing data for each variable of interest<br>(c) Summarise follow-up time (eg, average and total amount)	12
Outcome data	15*	Report numbers of outcome events or summary measures over time	13,14

For peer review only

For peer review only - http://bmjopen.bmj.com/site/about/guidelines.xhtml

Main results	16	(*a*) Give unadjusted estimates and, if applicable, confounder-adjusted estimates and their precision (eg, 95% confidence interval). Make clear which confounders were adjusted for and why they were included<br>(*b*) Report category boundaries when continuous variables were categorized<br>(*c*) If relevant, consider translating estimates of relative risk into absolute risk for a meaningful time period	13,14
Other analyses	17	Report other analyses done—eg analyses of subgroups and interactions, and sensitivity analyses	NA
**Discussion**			
Key results	18	Summarise key results with reference to study objectives	7
Limitations	19	Discuss limitations of the study, taking into account sources of potential bias or imprecision. Discuss both direction and magnitude of any potential bias	8
Interpretation	20	Give a cautious overall interpretation of results considering objectives, limitations, multiplicity of analyses, results from similar studies, and other relevant evidence	8
Generalisability	21	Discuss the generalisability (external validity) of the study results	8
**Other information**			
Funding	22	Give the source of funding and the role of the funders for the present study and, if applicable, for the original study on which the present article is based	8

*Give information separately for exposed and unexposed groups.

Note: An Explanation and Elaboration article discusses each checklist item and gives methodological background and published examples of transparent reporting. The STROBE checklist is best used in conjunction with this article (freely available on the Web sites of PLoS Medicine at http://www.plosmedicine.org/, Annals of Internal Medicine at http://www.annals.org/, and Epidemiology at http://www.epidem.com/). Information on the STROBE Initiative is available at http://www.strobe-statement.org.

For peer review only - http://bmjopen.bmj.com/site/about/guidelines.xhtml

For peer review only

BMJ Open

Sibling rank and sibling number in relation to cardiovascular disease and mortality risk: a nationwide cohort study

Journal:	*BMJ Open*
Manuscript ID	bmjopen-2020-042881.R1
Article Type:	Original research
Date Submitted by the Author:	21-Feb-2021
Complete List of Authors:	Nilsson, Peter; Lunds Universitet, Clinical Sciences<br>Sundquist, Jan; Lund University, Center for Primary Health Care Research;<br>Sundquist, Kristina; Lund University, Center for Primary Health Care Research<br>Li, Xinjun; Lund University, Center for Primary Health Care Research
**Primary Subject Heading**:	Epidemiology
Secondary Subject Heading:	Cardiovascular medicine
Keywords:	EPIDEMIOLOGY, REPRODUCTIVE MEDICINE, SOCIAL MEDICINE

SCHOLARONE™
Manuscripts

I, the Submitting Author has the right to grant and does grant on behalf of all authors of the Work (as defined in the below author licence), an exclusive licence and/or a non-exclusive licence for contributions from authors who are: i) UK Crown employees; ii) where BMJ has agreed a CC-BY licence shall apply, and/or iii) in accordance with the terms applicable for US Federal Government officers or employees acting as part of their official duties; on a worldwide, perpetual, irrevocable, royalty-free basis to BMJ Publishing Group Ltd ("BMJ") its licensees and where the relevant Journal is co-owned by BMJ to the co-owners of the Journal, to publish the Work in this journal and any other BMJ products and to exploit all rights, as set out in our licence.

The Submitting Author accepts and understands that any supply made under these terms is made by BMJ to the Submitting Author unless you are acting as an employee on behalf of your employer or a postgraduate student of an affiliated institution which is paying any applicable article publishing charge ("APC") for Open Access articles. Where the Submitting Author wishes to make the Work available on an Open Access basis (and intends to pay the relevant APC), the terms of reuse of such Open Access shall be governed by a Creative Commons licence – details of these licences and which Creative Commons licence will apply to this Work are set out in our licence referred to above.

Other than as permitted in any relevant BMJ Author's Self Archiving Policies, I confirm this Work has not been accepted for publication elsewhere, is not being considered for publication elsewhere and does not duplicate material already published. I confirm all authors consent to publication of this Work and authorise the granting of this licence.

For peer review only

Original manuscript for the BMJ OPEN 2021 02 18/PN

Revised Version

Sibling rank and sibling number in relation to cardiovascular disease and mortality risk: a nationwide cohort study

Peter M Nilsson [1], Jan Sundquist [1,2], Kristina Sundquist [1,2] and Xinjun Li [1]

[1] Department of Clinical Sciences, Lund University, Skåne University Hospital, Malmö, Sweden

[2] Center for Primary Care Research, Lund University, Skåne University Hospital, Malmö, Sweden

For peer review only

Short title: Sibling rank, CVD and mortality risk

Number of words: 247 in the Abstract, 2160 in full text

Number of references: 26

Number of tables: 3 + 3 supplemental

Number of figures: 3 + 1 supplemental

Conflict of interest: None

Corresponding author:

Peter M Nilsson, MD, PhD

Lund University, Department of Clinical Sciences,

Skåne University Hospital,

Jan Waldenströms gata 15, 5th floor

S-205 02 Malmö, Sweden.

Email: Peter.Nilsson@med.lu.se

Phone: +46-40-33 24 15

For peer review only - http://bmjopen.bmj.com/site/about/guidelines.xhtml

Abstract

Background: The number and rank order of siblings could be of importance for risk of cardiovascular disease and mortality. Previous studies have used only fatal events for risk prediction. We therefore aimed to use also non-fatal coronary and cardiovascular events in fully adjusted models.

Methods: From the Multi-Generation Register in Sweden data were used from 1.36 million men and 1.32 million women (born 1932 to 1960), aged 30-58 years at baseline and with follow-up from 1990 to 2015. Mean age at follow-up was 67 years (range 55-83 years). Fatal and non-fatal events were retrieved from national registers.

Results: For men, more siblings (with no siblings as reference) was associated with an increased cardiovascular risk (if >4 siblings), risk for coronary events (if >3 siblings), but lower total mortality risk (if >1 sibling), following full adjustment. For women, more siblings was associated with an increased risk of cardiovascular events (if >3 siblings), coronary events (if >2 siblings), but lower total mortality (if >1 sibling). First-born men had a lower risk of cardiovascular events than their later-born siblings, but higher total mortality than second and third-born siblings. For first-born women the risks of cardiovascular were also lower than for later-born siblings. The mortality risk was higher than for second-born siblings, but equal to higher numbered siblings.

Conclusion: Being first-born is associated with a favourable effect on non-fatal cardiovascular and coronary events for both men and women. The underlying biological mechanisms for this should be studied in a socio-cultural context.

Key words: Cardiovascular, Coronary, Epidemiology, Mortality, Register, Risk, Sibling

For peer review only - http://bmjopen.bmj.com/site/about/guidelines.xhtml

Strengths and limitations of the study

- This national register linkage study across generations includes data on both fatal and, for the first time, non-fatal cardiovascular and coronary events, as well as total mortality in relation to sibling number and rank
- Adjustment has been made for confounders including markers of social background (educational level, occupation) of the individual, but not for parental socioeconomic status. Competing risk analyses have been applied
- Limitations of the study include the lack of data from primary health care visits and that the historical register data do not fully reflect the ethnical diversity of Sweden today

For peer review only

Introduction

A positive family history of disease is a well-established variable to be used in risk algorithms for cardiovascular disease (CVD), even if it may be hard to quantify based on subjective recall only [1,2]. An alternative is to use register-based data for a more objective appraisal of the family burden of CVD [3]. One special feature of family structure is the *number of siblings* and *sibling rank* that can also be mapped by use of national registers such as the Multiple-Generation Register (MGR) of Sweden [4]. So far this register has been mostly used to describe the risk of some selected CVD manifestations of individuals in relation to the risk of their siblings, for example for thromboembolic disease [5] or other diseases [6-9].

In a previous report based on the MGR it was shown that increased number of sibling reflecting family size was not associated with increased total and cause-specific mortality risk in ages 40-74 years, but no analyses were made for risk of non-fatal CVD or coronary disease [10]. However, another corresponding study based on MGR data could show that total and cause-specific mortality in ages 30-69 years increased with increasing birth order [11].

The influence of sibling rank has been less well studied in relation to non-fatal cardiovascular and coronary risk. Previous studies have indicated a worse cardiovascular risk factor burden in first-borns, for example increased body mass index (BMI) and systolic blood pressure, but lower insulin sensitivity, than later born siblings [12-16]. On the other hand, first-borns seem to have a better physical fitness at military conscript testing [17], less caries [18] and run a lower risk of leukemia in adolescence [19] as well as lower suicide risk in a Finnish population [20].

Against this background we *aimed* to analyse the influence of sibling number and rank on risk of non-fatal and fatal cardiovascular and coronary manifestations, as well as total mortality, in the MGR of Sweden, after extensive adjustment for background demographic factors and family social status.

Subjects and methods

Setting and participants

The dataset used in this study was constructed by linking several national Swedish registers. The Swedish government-owned Statistics Sweden provided the Multi-Generation Register (MGR), in which persons born in Sweden in or after 1932 (the present study population) are linked to their parents [4]. We included all births (multiple births, full and half siblings) in the analyses. Linkages were made to National Census data in order to ascertain individual-level socioeconomic status. The final link was made by adding data from the Swedish Cause of Death Register (1961-2015) and the Swedish Hospital Discharge Register, with recorded dates of hospitalization and hospital diagnoses

For peer review only - http://bmjopen.bmj.com/site/about/guidelines.xhtml

since 1964, but on a national level since 1987, and now until end of 2015. National Swedish registers are of high validity for medical research [21, 22]. For analysing risk of CVD, CHD and mortality in relation to number of siblings and birth order, we collected data from 1.36 million men and 1.32 million women (born 1932 to 1960), aged 30-58 years at baseline and with follow-up from 1990 to 2015. For the definition of study subjects based on the MGR, see supplemental **Figure S1**.

Patient and public involvement

No patient involved.

Follow-up of CVD events and total mortality

We used the following ICD-codes for fatal or non-fatal CVD (ICD-9, 390-459, ICD-10, I00-I99); and for CHD (ICD-9, 410-414, ICD-10, I20-I25). Non-fatal events were followed in the national Hospital Discharge Register, and fatal events and total mortality until 31st December 2015 in the national Mortality Register.

Definitions

Family income: Family income was calculated at start of follow-up (1990) as annual family income divided by the number of members in the family. The income calculation was weighted, taking the ages of the family members into account. For example, children were given lower consumption weights than adults. The calculation was performed as follows: the sum of all family members' incomes was multiplied by the individual's consumption weight divided by the family members' total consumption weight. The final variable was calculated as empirical quartiles from the distribution and classified as low, middle-low, middle-high, and high.

Immigration status: born in Sweden or in other countries

Marital status: individuals were classified as married/cohabitating or never married, widowed, or divorced.

Socioeconomic status: was divided into four categories: the self-employed/farmers/all others, blue collar workers, white collar workers, or professionals

Education: was based on educational level, which was classified into three categories: ≤ 9 years, 10–11 years, and ≥ 12 years.

Geographic region: was divided into large cities (cities with a population of more than 200,000 inhabitants), Southern Sweden, and Northern Sweden.

Co-morbidity: was defined as the first hospitalization during the follow-up period of: chronic obstructive pulmonary disease, COPD (both hospitalization and mortality were included) (ICD-9 490–

For peer review only - http://bmjopen.bmj.com/site/about/guidelines.xhtml

496 and ICD-10 J40-J49), obesity (ICD-9 278A and ICD-10 E65-E68), alcoholism related liver disease (both hospitalizations and mortality were included) (ICD-9 291, 303, 571 and ICD-10 F10 and K70), hypertension (ICD-9 401-405 and ICD-10 I10–I15), and diabetes (both hospitalizations and mortality were included) (ICD-9 250 and ICD-10 E10-E14), and cancer (cancer were included both from cancer register and mortality, ICD-9 140-239, and ICD-10 C00-D48).

Statistical methods

Person-years at risk were calculated from the start of follow-up on 1 January 1990 until hospitalization or death from CVD, death from other causes, emigration, or the end of the follow-up, 31st December 2015. Age-adjusted incidence rates for first hospitalization and mortality were calculated for the entire follow-up period. We used the Cox's proportional hazard model to calculate the hazard ratio (HR) with 95 percent confidence intervals (CI) for total (fatal and non-fatal) CVD and CHD event risk, and for total mortality, for both men and women) in relation to number siblings and birth order. This was done after adjustment for age at start, individual characteristics (family income, marital status, immigrant background and educational level, region of residence, socioeconomic status), and finally for co-morbidities in order to adjust for competing mortality risk. Individuals without sibling was used as the reference. The proportionality assumptions were checked by plotting the incidence rates over time and by calculating Schoenfeld (partial) residuals and these assumptions were fulfilled. We used SAS version 9.4 (SAS Institute Inc. Cary, NC, USA) for all statistical analyses.

A further adjustment was made for total number of siblings in relation to birth order when the risk for different outcomes was calculated, using the category "first birth" as reference. A competing risk model used for mortality as a competing risk for incident CVD. A p-value < 0.05 was considered significant.

Results

With an average of 20 years (Q1-Q3 16-25 years) follow-up, in 1,358,647 men we used data on 592,863 CVD events, 131,533 coronary events, and 240,371 total deaths. For 1,315,037 women, the corresponding numbers were 486,147 CVD events, 55,933 coronary events and 160,269 deaths, respectively. The mean age of the study population at the end of the follow-up was 67 years (range 55-83 years). The number of siblings and birth order of men and women are depicted in **Table 1**.

Risk associated with number of siblings in men and women

For men, a higher number of siblings (with no siblings as reference) was associated with a lower risk of cardiovascular events (if 1-2 siblings), but an increased risk (if $\geq$4 siblings); an increased risk for

For peer review only - http://bmjopen.bmj.com/site/about/guidelines.xhtml

coronary events (if ≥3 siblings); but lower total mortality risk (if ≥1 sibling), following full adjustment, **Table 2.**

For women, a higher number of siblings (with no siblings as reference) was associated with an increased risk of cardiovascular events (if ≥3 siblings), coronary events (if ≥2 siblings), but lower total mortality (if ≥1 sibling), following full adjustment, **Table 3.**

Risk associated with sibling rank in men and women

According to sibling rank, first-born men had a lower risk of both cardiovascular and coronary events than their later-born siblings, but higher total mortality than second and third-born siblings, following full adjustment, **Table 2**.

For first-born women the risks of cardiovascular and coronary events were also lower than in their later-born siblings. The mortality risk was higher than for second-born siblings, but equal to higher numbered siblings, following full adjustment, **Table 3**.

For hazard ratios of CHD, CVD and total mortality by number of siblings and birth order in men and women, respectively, see **Figures 1-3.**

Supplemental material

For detailed data on the distribution of the study population, number of CVD, coronary heart disease (CHD), and mortality events in men and women, see Supplemental **Table S1**. For detailed data on the risk associated with factors adjusted for, see Supplemental **Tables S2-S4,** for men and women, respectively.

Discussion

In this very large observational study based on a national Multi-Generation Register it was found that first-born men and women are at lower risk of both cardiovascular and coronary events than their later-born siblings, but had higher total mortality risk than second and third-born siblings (men). For women the mortality risk for first-born women was higher than for second-born siblings, following full adjustment for a number of background factors.

For total mortality in relation to sibling number our data are at odds with a previous study using the same register in Sweden, showing no increased mortality associated until 74 years with a higher number of siblings [10]. However, we used higher numbers, longer follow-up and more extensive adjustment.

For peer review only - http://bmjopen.bmj.com/site/about/guidelines.xhtml

For the influence of sibling rank, a previous study could show higher risk of total and cause-specific mortality with increasing sibling rank until 69 years [11]. This was similar in our study for risk of non-fatal cardiovascular and coronary events during longer follow-up and extensive adjustment.

These findings of lower cardiovascular risk in first-borns are at contrast to previous reports of a higher level of cardiovascular risk factors in such individuals followed until adolescence or young adulthood [12-16]. The burden of risk factors might have been compensated for by a better physical fitness, as noticed in first-born men coming for military conscript testing at the age of around 18 years [17]. In contrast to these observations, our extensive data indicate a lower cardiovascular risk in first-borns. Other unmeasured factors linked to being first-born, such as cognition or bodily development, could have contributed to our findings of a relative protection, even if we adjusted for a long list of potential confounders such as educational level, socio-economic status, marital status, and co-morbidities.

Besides filling a knowledge gap, this is of public health interest as different countries endorse different policies to support families and number of children. Our findings relate to family size and the biological as well as social roles related to sibling rank with its health implications.

More research is needed to understand the links between sibling number and rank with health outcomes. This could address, for example, the dilution of resources theory [23] of special relevance for disadvantaged girls; epigenetic factors influencing the metabolic syndrome in offspring [24]; and maternal health during pregnancy, including the effects of multiple births/child-rearing on maternal health and family resources, especially in deprived settings with large families [25].

Limitations and strengths of the study

The Swedish hospital discharge register contains no information about diagnostic procedures, which is a limitation. Moreover, specialist doctors in hospital care made the diagnosis. Another limitation is that we had no data on life-style related factors such as body mass index, smoking, and diet, because it would be unrealistic to gather such data for an entire national population. However, we did adjust for socioeconomic status, obesity, diabetes, COPD, and alcoholism and related liver disorders, which are associated with factors such as smoking and alcohol. Given a focus on family size, knowing that siblings who died young, and therefore not contributed to resource dilution for a proportion of the index person's childhood, would be of interest and importance. It would also shed light (potentially) on family circumstances and health. Regretfully, we currently lack data on parental SES to adjust for.

Strengths of the study include complete nationwide coverage from 1990 in a country with high standards of diagnosis, and with diagnoses often being made by specialists during extended examinations in clinics. Another important strength of our study is that it was based on nationwide registers and was thus free of selection and recall bias. The Swedish MGR and the Swedish Hospital

For peer review only - http://bmjopen.bmj.com/site/about/guidelines.xhtml

Discharge Register are validated data sources that have been proven to be reliable in the study of many diseases [4, 21,22]. Data in our dataset are almost 100% complete [4]. Generalisability (external validity) should hold at least for countries and populations similar to Sweden.

Future research should be directed to find biological or social mechanisms linking the status of being first-born to lower risk of cardiovascular disease, as indicated by our observational findings. A previous Norwegian study in military conscripts indicated that the role of being first-born is influenced by social factors, as a second-born son may achieve characteristics of a first-born brother who died young [26].

In conclusion, our data indicate a favourable effect on non-fatal cardiovascular and coronary events by being first-born, both for men and women.

a. Contributorship statement

PMN provided the original idea, and XL made the statistical analyses. PMN and XL drafted the first manuscript. All authors (PMN, XL, JS, and KS) contributed to the final manuscript. PMN and XL made the revisions.

b. Competing interests

None

c. Funding

This work was supported by the grants to Kristina Sundquist from The Swedish Research Council; ALF funding from Region Skåne and the Swedish Heart-Lung foundation.

d. Data sharing statement

Data will not be shared, but reasonable requests may be directed to Professor Kristina Sundquist, the PI of the registers.

For peer review only - http://bmjopen.bmj.com/site/about/guidelines.xhtml

References

1. Banerjee A. A review of family history of cardiovascular disease: risk factor and research tool. *Int J Clin Pract*. 2012; 66:536-43.

2. Tada H, Melander O, Louie JZ, *et al.* Risk prediction by genetic risk scores for coronary heart disease is independent of self-reported family history. *Eur Heart J*. 2016;37(6):561–567.

3. Nilsson PM, Nilsson J-Å, and Berglund G. Family burden of cardiovascular mortality – risk implications for offspring in a national register linkage study based upon the Malmö Preventive Project. *J Internal Med* 2004; 255:229-35.

4. Ekbom A. The Swedish Multi-generation Register. *Methods Mol Biol*. 2011;675:215–220.

5. Zöller B, Ji J, Sundquist J, Sundquist K. Body Height and Incident Risk of Venous Thromboembolism: A Cosibling Design. *Circ Cardiovasc Genet.* 2017;10(5):e001651.

6. Kasiman K, Lundholm C, Sandin S, Malki N, Sparén P, Ingelsson E. Familial effects on ischemic stroke: the role of sibling kinship, sex, and age of onset. *Circ Cardiovasc Genet.* 2012;5(2):226–233.

7. Martinsson A, Li X, Zöller B, *et al.* Familial Aggregation of Aortic Valvular Stenosis: A Nationwide Study of Sibling Risk. *Circ Cardiovasc Genet*. 2017; 10(6):e001742.

8. Lindgren MP, Ji J, Smith JG, Sundquist J, Sundquist K, Zöller B. Mortality risks associated with sibling heart failure [published online ahead of print, 2019 Oct 12]. *Int J Cardiol.* 2019; S0167-5273(19)30895-2.

9. Berntsson J, Li X, Zöller B, *et al.* Risk of Stroke in Patients With Atrial Fibrillation Is Associated With Stroke in Siblings: A Nationwide Study. *J Am Heart Assoc.* 2020; 9(3):e014132.

10. Baranowska-Rataj A, Barclay K, Kolk M. The effect of number of siblings on adult mortality: Evidence from Swedish registers for cohorts born between 1938 and 1972. *Popul Stud (Camb).* 2017; 71(1):43–63.

11. Barclay K, Kolk M. Birth order and mortality: a population-based cohort study. Demography. 2015; 52(2):613–639.

12. Ayyavoo A, Derraik JG, Hofman PL, Cutfield WS. Is being first-born another risk factor for metabolic and cardiovascular diseases? *Future Cardiol.* 2013; 9(4):447–450.

13. Theodore RF, Broadbent J, Nagin D, et al. Childhood to Early-Midlife Systolic Blood Pressure Trajectories: Early-Life Predictors, Effect Modifiers, and Adult Cardiovascular Outcomes. *Hypertension.* 2015;66(6):1108–1115.

For peer review only - http://bmjopen.bmj.com/site/about/guidelines.xhtml

14. Albert BB, de Bock M, Derraik JG, et al. Among overweight middle-aged men, first-borns have lower insulin sensitivity than second-borns. *Sci Rep.* 2014; 4:3906.

15. Siervo M, Horta BL, Stephan BC, Victora CG, Wells JC. First-borns carry a higher metabolic risk in early adulthood: evidence from a prospective cohort study. *PLoS One*. 2010; 5(11):e13907.

16. Jelenkovic A, Silventoinen K, Tynelius P, Myrskylä M, Rasmussen F. Association of birth order with cardiovascular disease risk factors in young adulthood: a study of one million Swedish men. *PLoS One.* 2013; 8(5):e63361.

17. Barclay K, Myrskylä M. Birth order and physical fitness in early adulthood: evidence from Swedish military conscription data. *Soc Sci Med.* 2014; 123:141–148.

18. Julihn A, Soares FC, Hammarfjord U, Hjern A, Dahllöf G. Birth order is associated with caries development in young children: a register-based cohort study. *BMC Public Health.* 2020; 20(1):218.

19. Von Behren J, Spector LG, Mueller BA, *et al.* Birth order and risk of childhood cancer: a pooled analysis from five US States. *Int J Cancer.* 2011; 128(11):2709–2716.

20. Saarela J, Cederström A, Rostila M. Birth order and mortality in two ethno-linguistic groups: Register-based evidence from Finland. *Soc Sci Med.* 2016; 158:8–13.

21. Ludvigsson JF, Almqvist C, Bonamy AK, et al. Registers of the Swedish total population and their use in medical research. Eur J Epidemiol. 2016; 31(2):125-36.

22. Ludvigsson JF, Andersson E, Ekbom A, *et al.* External review and validation of the Swedish national inpatient register. *BMC Public Health.* 2011; 11:450.

23. Kalmijn M, van de Werfhorst HG. Sibship Size and Gendered Resource Dilution in Different Societal Contexts. PLoS One. 2016; 11(8):e0160953.

24. Dunford AR, Sangster JM. Maternal and paternal periconceptional nutrition as an indicator of offspring metabolic syndrome risk in later life through epigenetic imprinting: A systematic review. Diabetes Metab Syndr. 2017; 11 Suppl 2:S655-S662.

25. Young MF, Ramakrishnan U. Maternal Undernutrition before and during Pregnancy and Offspring Health and Development. Ann Nutr Metab. 2021 Feb 1:1-13.

26. Kristensen P, Bjerkedal T. Explaining the relation between birth order and intelligence. *Science* 2007; 316(5832):1717.

For peer review only - http://bmjopen.bmj.com/site/about/guidelines.xhtml

For peer review only

For peer review only - http://bmjopen.bmj.com/site/about/guidelines.xhtml

Table 1. Distribution of population, number of CVD, CHD, and mortality events

	Population		CVD events		CHD events		Mortality events	
	No.	(%)	No. (% of population)	%	No. (% of population)	%	No. (% of population)	%
**Men**	1358647		592863 (43.6)		131533 (9.7)		240371 (17.7)	
Number of sibling								
Non sibling	214700	15.8	105516 (49.1)	17.8	23671 (11.0)	18.0	50709 (23.6)	21.1
One sibling	443877	32.7	189839 (42.8)	32.0	39729 (9.0)	30.2	73140 (16.5)	30.4
Two siblings	338812	24.9	140361 (41.4)	23.7	30184 (8.9)	22.9	52790 (15.6)	22.0
Three siblings	183067	13.5	77378 (42.3)	13.1	17663 (9.6)	13.4	30266 (16.5)	12.6
Four or more siblings	178191	13.1	79769 (44.8)	13.5	20286 (11.4)	15.4	33466 (18.8)	13.9
Birth order								
First	684765	50.4	318341 (46.5)	53.7	70238 (10.3)	53.4	140857 (20.6)	58.6
Second	402879	29.7	166757 (41.4)	28.1	36654 (9.1)	27.9	62267 (15.5)	25.9
Third	164540	12.1	65853 (40.0)	11.1	14736 (9.0)	11.2	23081 (14.0)	9.6
Fourth	62765	4.6	24729 (39.4)	4.2	5737 (9.1)	4.4	8425 (13.4)	3.5
Fifth+	43698	3.2	17183 (39.3)	2.9	4168 (9.5)	3.2	5741 (13.1)	2.4
**Women**	1315037		486147 (37.0)		55933 (4.3)		160269 (12.2)	
Number of sibling								
Non sibling	210121	16.0	87261 (41.5)	17.9	10289 (4.9)	18.4	34521 (16.4)	21.5
One sibling	430315	32.7	154154 (35.8)	31.7	16280 (3.8)	29.1	49132 (11.4)	30.7
Two siblings	324379	24.7	113739 (35.1)	23.4	12500 (3.9)	22.3	34843 (10.7)	21.7
Three siblings	176631	13.4	63871 (36.2)	13.1	7512 (4.3)	13.4	19766 (11.2)	12.3
Four or more siblings	173591	13.2	67122 (38.7)	13.8	9352 (5.4)	16.7	22007 (12.7)	13.7
Birth order								
First	664459	50.5	262015 (39.4)	53.9	30342 (4.6)	54.2	94779 (14.3)	59.1
Second	388391	29.5	136263 (35.1)	28.0	15191 (3.9)	27.2	40984 (10.6)	25.6
Third	159311	12.1	53711 (33.7)	11.0	6231 (3.9)	11.1	15205 (9.5)	9.5
Fourth	60676	4.6	20264 (33.4)	4.2	2379 (3.9)	4.3	5558 (9.2)	3.5
Fifth+	42200	3.2	13894 (32.9)	2.9	1790 (4.2)	3.2	3743 (8.9)	2.3

For peer review only - http://bmjopen.bmj.com/site/about/guidelines.xhtml

Table 2. Hazard ratio (HR) and 95% confidence interval of CVD, CHD, and mortality in *men*

	CVD			CHD			Mortality		
	HR[a,b]	95% CI		HR[a]	95% CI		HR[a]	95% CI	
**Number of siblings (ref. Non sibling)**									
One sibling	0.98	0.97	0.99	0.99	0.97	1.01	0.93	0.92	0.94
Two siblings	0.97	0.97	0.98	1.01	0.99	1.03	0.91	0.90	0.92
Three siblings	0.98	0.97	0.99	1.04	1.02	1.07	0.93	0.92	0.94
Four or more siblings	1.00	0.99	1.01	1.10	1.07	1.12	0.96	0.94	0.97
**Birth order (ref. First birth)**									
Second	1.00	1.00	1.01	1.08	1.06	1.09	0.96	0.95	0.97
Third	1.02	1.02	1.03	1.13	1.11	1.15	0.98	0.96	0.99
Fourth	1.04	1.02	1.05	1.17	1.14	1.21	0.98	0.95	1.00
Fifth+	1.07	1.05	1.09	1.23	1.19	1.28	1.01	0.98	1.05

a: Full adjusted model: Adjusted for age at start, individual characteristics of family income, marital status, educational attainment, immigrant status, socioeconomic status, region of residence, comorbidities, number of siblings, and birth order
b: Multivariable competing risk survival analysis

For peer review only

Table 3. Hazard ratio (HR) and 95% confidence interval of CVD, CHD, and mortality in *women*

	CVD			CHD			Mortality		
	HR[a,b]	95% CI		HR[a]	95% CI		HR[a]	95% CI	
**Number of siblings (ref. Non sibling)**									
One sibling	0.98	0.98	0.99	0.99	0.97	1.02	0.94	0.93	0.95
Two siblings	0.99	0.98	1.00	1.03	1.00	1.06	0.92	0.91	0.94
Three siblings	1.00	0.99	1.01	1.07	1.04	1.11	0.93	0.91	0.95
Four or more siblings	1.01	1.00	1.03	1.17	1.13	1.21	0.95	0.93	0.96
**Birth order (ref. First birth)**									
Second	1.01	1.00	1.02	1.07	1.05	1.09	0.96	0.95	0.98
Third	1.02	1.01	1.03	1.14	1.11	1.18	0.98	0.96	1.00
Fourth	1.04	1.02	1.05	1.14	1.09	1.20	1.00	0.98	1.03
Fifth+	1.05	1.03	1.07	1.22	1.15	1.29	1.03	0.99	1.07

a: Full adjusted model: Adjusted for age at start, individual characteristics of family income, marital status, educational attainment, immigrant status, socioeconomic status, region of residence, comorbidities, number of siblings, and birth order
b: Multivariable competing risk survival analysis

For peer review only

For peer review only - http://bmjopen.bmj.com/site/about/guidelines.xhtml

Figure legends:

Figure 1. Hazard ratios of CHD by number of siblings and birth order in *men* (a,b) and *women* (c,d)

Figure 2. Hazard ratios of CVD by number of siblings and birth order in *men* (a,b) and *women* (c,d)

Figure 3. Hazard ratios of mortality by number of siblings and birth order in *men* (a,b) and *women* (c,d)

Supplemental Figure S1. Flow-chart of the study sample identified in the Swedish Multi-Generation Register (MGR).

For peer review only

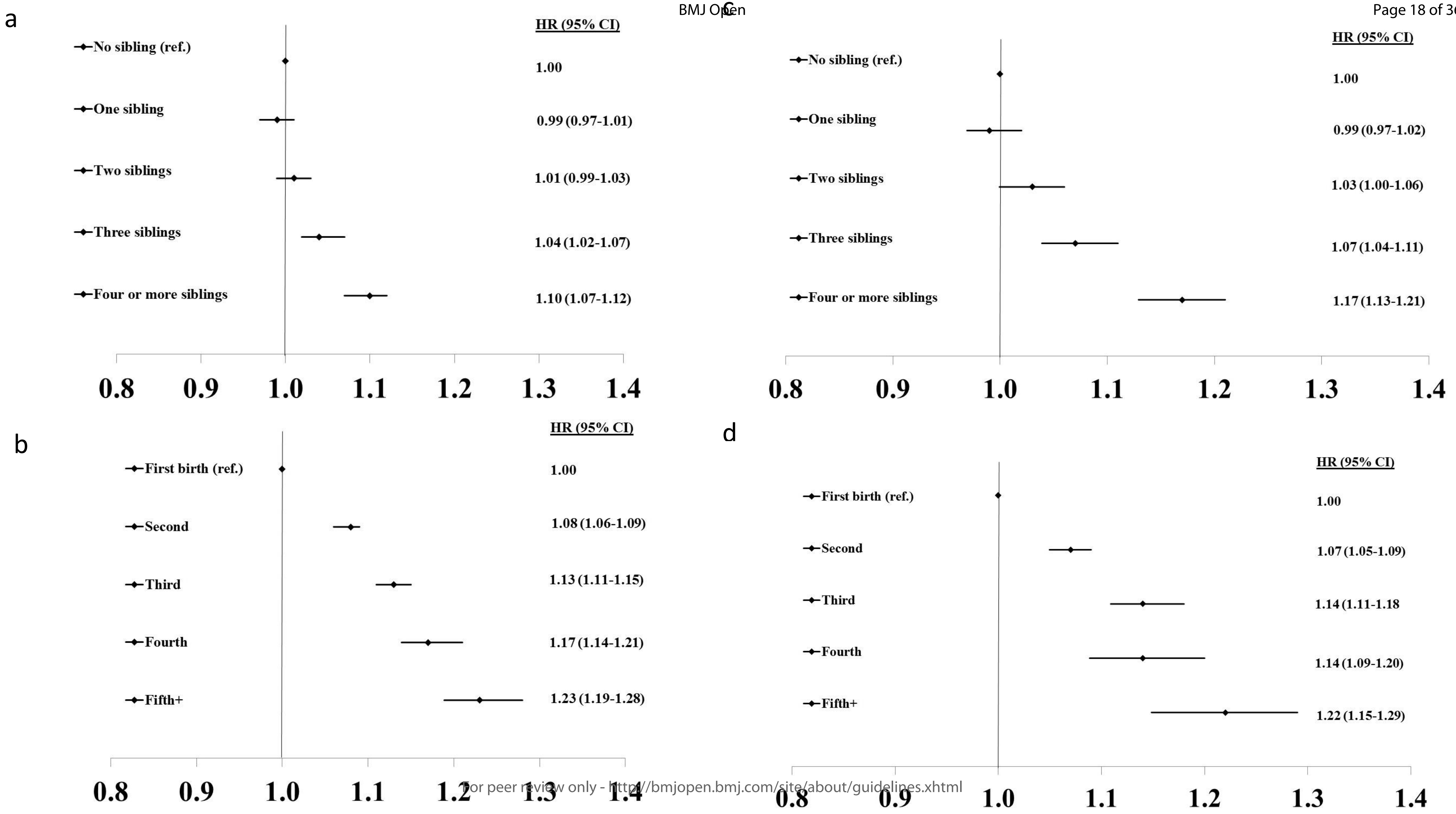
a
HR (95% CI)
No sibling (ref.) 1.00
One sibling 0.99 (0.97-1.01)
Two siblings 1.01 (0.99-1.03)
Three siblings 1.04 (1.02-1.07)
Four or more siblings 1.10 (1.07-1.12)
0.8 0.9 1.0 1.1 1.2 1.3 1.4
b
HR (95% CI)
First birth (ref.) 1.00
Second 1.08 (1.06-1.09)
Third 1.13 (1.11-1.15)
Fourth 1.17 (1.14-1.21)
Fifth+ 1.23 (1.19-1.28)
0.8 0.9 1.0 1.1 1.2 1.3 1.4
c
HR (95% CI)
No sibling (ref.) 1.00
One sibling 0.99 (0.97-1.02)
Two siblings 1.03 (1.00-1.06)
Three siblings 1.07 (1.04-1.11)
Four or more siblings 1.17 (1.13-1.21)
0.8 0.9 1.0 1.1 1.2 1.3 1.4
d
HR (95% CI)
First birth (ref.) 1.00
Second 1.07 (1.05-1.09)
Third 1.14 (1.11-1.18)
Fourth 1.14 (1.09-1.20)
Fifth+ 1.22 (1.15-1.29)
0.8 0.9 1.0 1.1 1.2 1.3 1.4

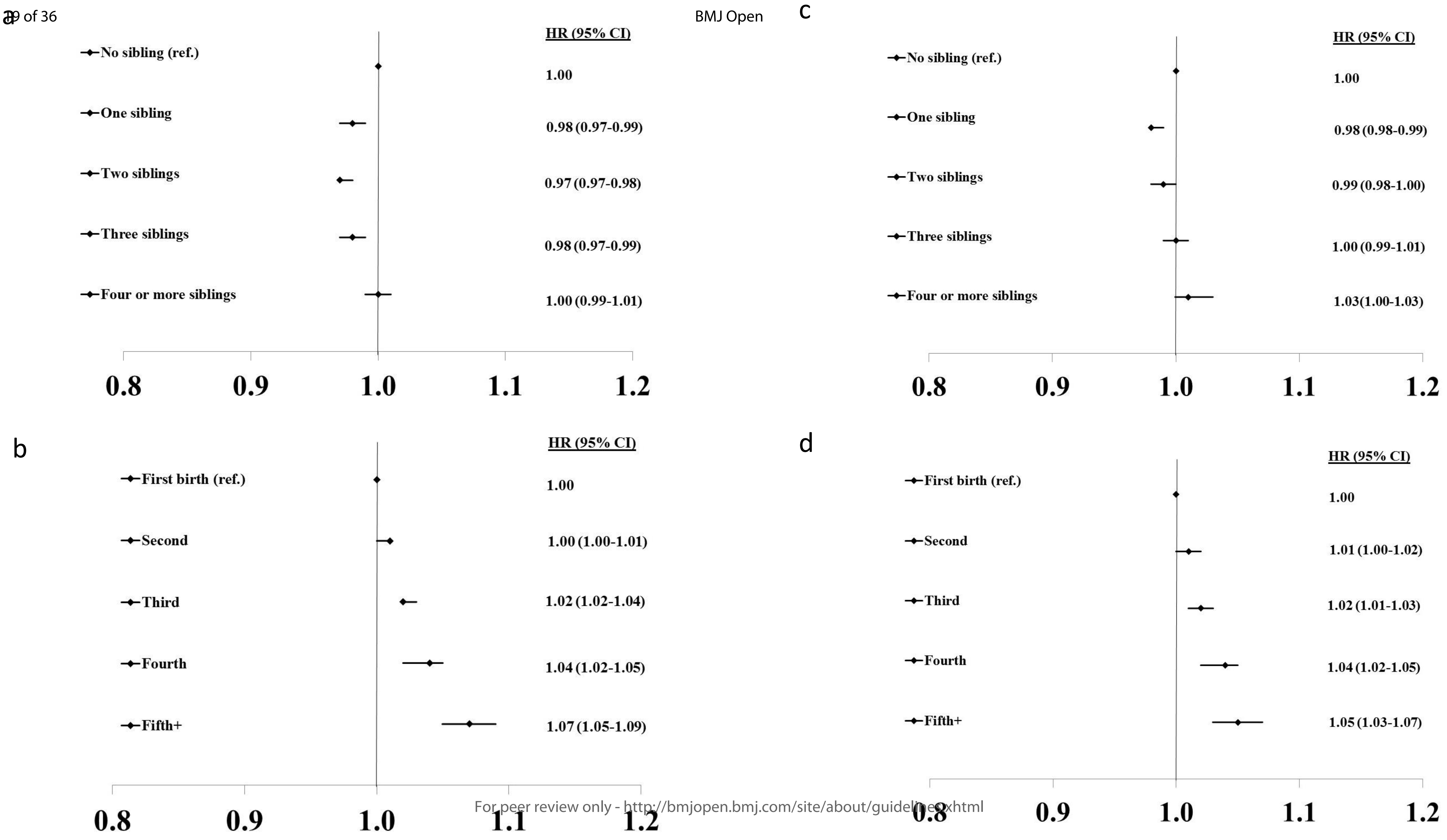
a
HR (95% CI)
No sibling (ref.)
1.00
One sibling
0.98 (0.97-0.99)
Two siblings
0.97 (0.97-0.98)
Three siblings
0.98 (0.97-0.99)
Four or more siblings
1.00 (0.99-1.01)
0.8
0.9
1.0
1.1
1.2
b
HR (95% CI)
First birth (ref.)
1.00
Second
1.00 (1.00-1.01)
Third
1.02 (1.02-1.04)
Fourth
1.04 (1.02-1.05)
Fifth+
1.07 (1.05-1.09)
0.8
0.9
1.0
1.1
1.2
c
HR (95% CI)
No sibling (ref.)
1.00
One sibling
0.98 (0.98-0.99)
Two siblings
0.99 (0.98-1.00)
Three siblings
1.00 (0.99-1.01)
Four or more siblings
1.03(1.00-1.03)
0.8
0.9
1.0
1.1
1.2
d
HR (95% CI)
First birth (ref.)
1.00
Second
1.01 (1.00-1.02)
Third
1.02 (1.01-1.03)
Fourth
1.04 (1.02-1.05)
Fifth+
1.05 (1.03-1.07)
0.8
0.9
1.0
1.1
1.2

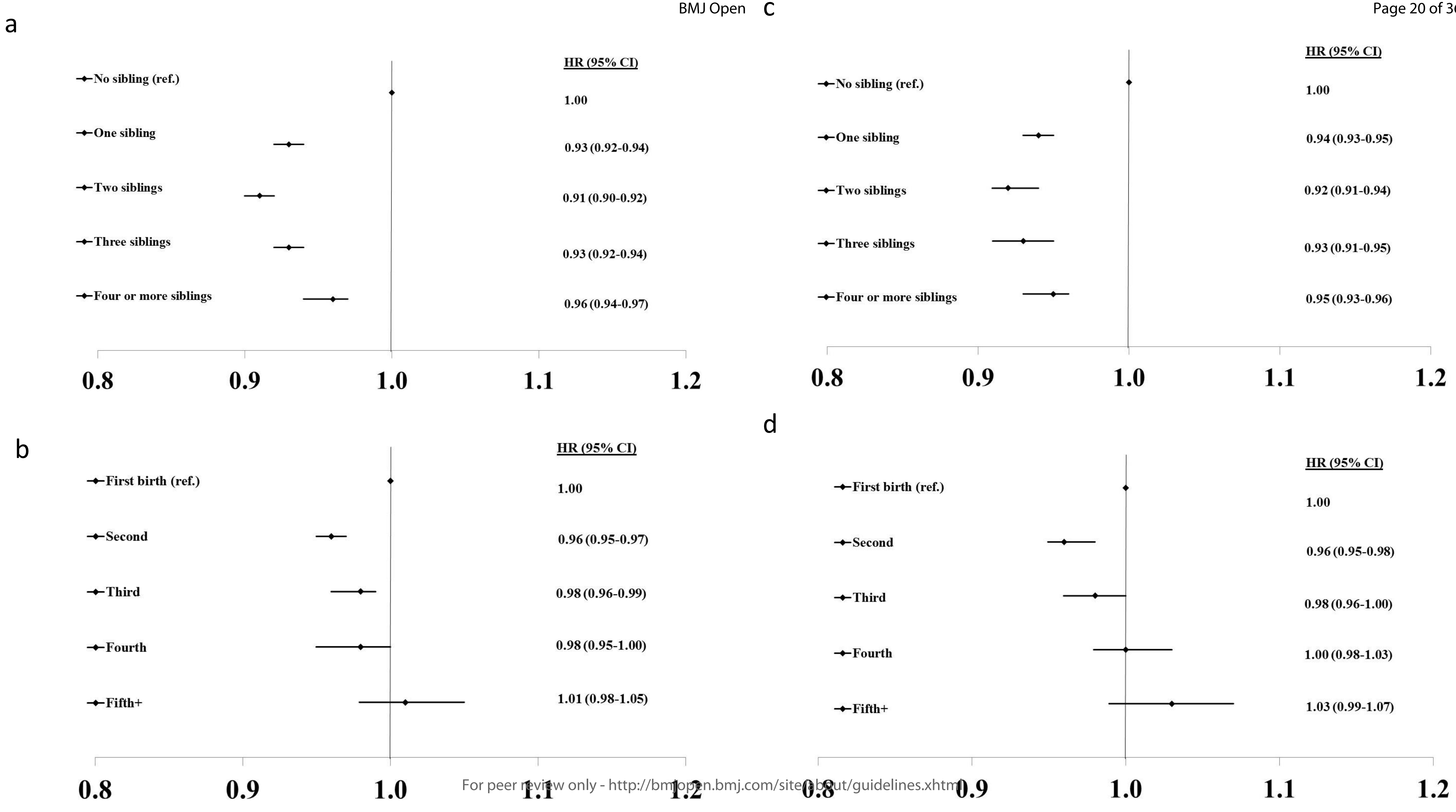

For peer review only - http://bmjopen.bmj.com/site/about/guidelines.xhtml

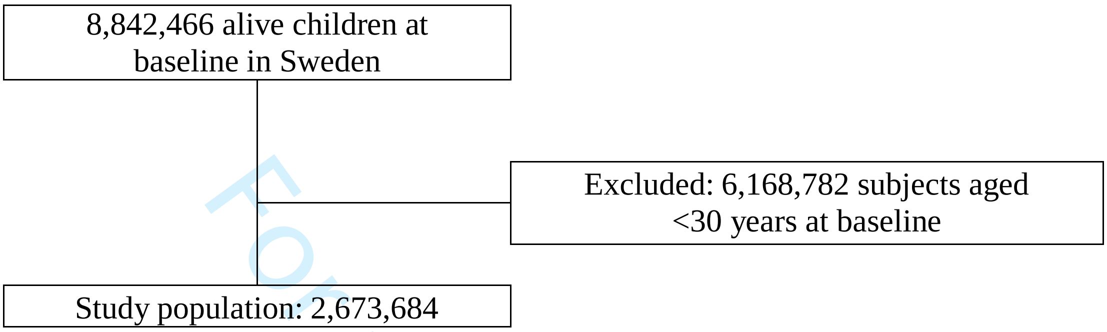

For peer review only

Supplementary Table 1a. Distribution of population, number of CVD, CHD, and mortality events in *men*.

	Population		CVD events		CHD events		Mortality events	
	No.	(%)	No.	%	No.	%	No.	%
Total population (%)	1358647		592863		131533		240371	
Age (years)								
30-39	531559	39.1	152615	25.7	25327	19.3	39094	16.3
40-49	554137	40.8	265251	44.7	59088	44.9	96549	40.2
50-58	272951	20.1	174997	29.5	47118	35.8	104728	43.6
Family income								
Low income	334083	24.6	123575	20.8	26649	20.3	47582	19.8
Middle–low income	320284	23.6	135148	22.8	29613	22.5	51243	21.3
Middle–high income	321653	23.7	152589	25.7	35041	26.6	66400	27.6
High income	382627	28.2	181551	30.6	40230	30.6	75146	31.3
Marital status								
Married/cohabiting	809314	59.6	370633	62.5	85212	64.8	124039	51.6
Never married, Widowed, or divorced	549333	40.4	222230	37.5	46321	35.2	116332	48.4
Educational attainment								
≤ 9 years	281207	20.7	146179	24.7	38890	29.6	81428	33.9
10–11 years	173208	12.7	67512	11.4	13955	10.6	26218	10.9
≥ 12 years	904232	66.6	379172	64.0	78688	59.8	132725	55.2
Immigrant status								
Sweden	1322658	97.4	580707	97.9	128732	97.9	235464	98.0
Other countries	35989	2.6	12156	2.1	2801	2.1	4907	2.0
Socioeconomic status								
Farmers/self-employed/others	308729	22.7	129408	21.8	29064	22.1	71960	29.9
Blue collar workers	494090	36.4	217215	36.6	50626	38.5	89989	37.4
White collar workers	351621	25.9	157170	26.5	34594	26.3	52582	21.9
Professionals	204207	15.0	89070	15.0	17249	13.1	25840	10.8
Urban/rural status								
Large cities	495681	36.5	207593	35.0	41153	31.3	85765	35.7
Southern Sweden	618686	45.5	274100	46.2	61581	46.8	108663	45.2
Northern Sweden	244280	18.0	111170	18.8	28799	21.9	45943	19.1
Hospitalization of alcoholism and related liver disease								
No	1275305	93.9	548796	92.6	124051	94.3	202724	84.3
Yes	83342	6.1	44067	7.4	7482	5.7	37647	15.7
Hospitalization of diabetes								
No	1226642	90.3	494000	83.3	104716	79.6	200226	83.3
Yes	132005	9.7	988863	16.7	26817	20.4	40145	16.7

For peer review only

Hospitalization of hypertension								
No	1059935	78.0	294151	49.6	76609	58.2	183958	76.5
Yes	298712	22.0	298712	50.4	54924	41.8	56413	23.5
Hospitalization of obesity								
No	1338892	98.5	577543	97.4	128595	97.8	236644	98.4
Yes	19755	1.5	15320	2.6	2938	2.2	3727	1.6
Hospitalization of chronic lower respiratory disease								
No	1288108	94.8	546393	92.2	120575	91.7	216522	90.1
Yes	70539	5.2	46470	7.8	10958	8.3	23849	9.9
Cancer								
No	1109571	81.7	452446	76.3	103202	78.5	139486	58.0
Yes	249076	18.3	140417	23.7	28331	21.5	100885	42.0
Number of sibling								
Non sibling	214700	15.8	105516	17.8	23671	18.0	50709	21.1
One sibling	443877	32.7	189839	32.0	39729	30.2	73140	30.4
Two siblings	338812	24.9	140361	23.7	30184	22.9	52790	22.0
Three siblings	183067	13.5	77378	13.1	17663	13.4	30266	12.6
Four or more siblings	178191	13.1	79769	13.5	20286	15.4	33466	13.9
Birth order								
First	684765	50.4	318341	53.7	70238	53.4	140857	58.6
Second	402879	29.7	166757	28.1	36654	27.9	62267	25.9
Third	164540	12.1	65853	11.1	14736	11.2	23081	9.6
Fourth	62765	4.6	24729	4.2	5737	4.4	8425	3.5
Fifth+	43698	3.2	17183	2.9	4168	3.2	5741	2.4

For peer review only

Supplementary Table 1b. Distribution of population, number of CVD, CHD, and mortality events in *women*.

	Population		CVD events		CHD events		Mortality events	
	No.	(%)	No.	%	No.	%	No.	%
Total population (%)	1315037		486147		55933		160269	
Age (years)								
30-39	507872	38.6	125326	25.8	9681	17.3	24571	15.3
40-49	537035	40.8	210113	43.2	23738	42.4	64055	40.0
50+	270130	20.5	150708	31.0	22514	40.3	71643	44.7
Family income								
Low income	343296	26.1	100542	20.7	10467	18.7	29723	18.5
Middle–low income	340837	25.9	121716	25.0	13432	24.0	37773	23.6
Middle–high income	347955	26.5	142924	29.4	17579	31.4	50537	31.5
High income	282949	21.5	120965	24.9	14455	25.8	42236	26.4
Marital status								
Married/cohabiting	887258	67.5	331886	68.3	38106	68.1	96533	60.2
Never married, Widowed, or divorced	427779	32.5	154261	31.7	17827	31.9	63736	39.8
Educational attainment								
≤ 9 years	232824	17.7	102037	21.0	15415	27.6	48455	30.2
10–11 years	165367	12.6	60209	12.4	6867	12.3	20489	12.8
≥ 12 years	916846	69.7	323901	66.6	33651	60.2	91325	57.0
Immigrant status								
Sweden	1281472	97.4	476075	97.9	54756	97.9	157515	98.3
Other countries	33565	2.6	10072	2.1	1177	2.1	2754	1.7
Socioeconomic status								
Farmers/self-employed/others	272189	20.7	96279	19.8	12450	22.3	45553	28.4
Blue collar workers	437324	33.3	174549	35.9	21821	39.0	55960	34.9
White collar workers	496782	37.8	178249	36.7	18324	32.8	49169	30.7
Professionals	108742	8.3	37070	7.6	3338	6.0	9587	6.0
Urban/rural status								
Large cities	495097	37.6	173872	35.8	17499	31.3	57794	36.1
Southern Sweden	591636	45.0	223375	45.9	25965	46.4	72872	45.5
Northern Sweden	228304	17.4	88900	18.3	12469	22.3	29603	18.5
Hospitalization of alcoholism and related liver disease								
No	1283561	97.6	471088	96.9	54416	97.3	149212	93.1
Yes	31476	2.4	15059	3.1	1517	2.7	11057	6.9
Hospitalization of diabetes								
No	1234361	93.9	429522	88.4	45537	81.4	139644	87.1

For peer review only

Yes	80676	6.1	56625	11.6	10396	18.6	20625	12.9
Hospitalization of hypertension								
No	1067928	81.2	239038	49.2	31364	56.1	124898	77.9
Yes	247109	18.8	247109	50.8	24569	43.9	35371	22.1
Hospitalization of obesity								
No	1286838	97.9	468228	96.3	54003	96.5	156771	97.8
Yes	28199	2.1	17919	3.7	1930	3.5	3498	2.2
Hospitalization of chronic lower respiratory disease								
No	1226846	93.3	433906	89.3	48084	86.0	137028	85.5
Yes	88191	6.7	52241	10.7	7849	14.0	23241	14.5
Cancer								
No	1051617	80.0	364226	74.9	43617	78.0	70300	43.9
Yes	263420	20.0	121921	25.1	12316	22.0	89969	56.1
Number of sibling								
Non sibling	210121	16.0	87261	17.9	10289	18.4	34521	21.5
One sibling	430315	32.7	154154	31.7	16280	29.1	49132	30.7
Two siblings	324379	24.7	113739	23.4	12500	22.3	34843	21.7
Three siblings	176631	13.4	63871	13.1	7512	13.4	19766	12.3
Four or more siblings	173591	13.2	67122	13.8	9352	16.7	22007	13.7
Birth order								
First	664459	50.5	262015	53.9	30342	54.2	94779	59.1
Second	388391	29.5	136263	28.0	15191	27.2	40984	25.6
Third	159311	12.1	53711	11.0	6231	11.1	15205	9.5
Fourth	60676	4.6	20264	4.2	2379	4.3	5558	3.5
Fifth+	42200	3.2	13894	2.9	1790	3.2	3743	2.3

For peer review only

Supplementary Table 2a. Hazard ratio (HR) and 95% confidence interval of CVD in *men*, using multivariable competing risk survival analysis

	Model 1			Model 2			Model 3		
	HR*	95% CI		HR*	95% CI		HR*	95% CI	
Number of siblings (ref. No sibling)									
One sibling	0.97	0.97	0.98	0.98	0.97	0.98	0.98	0.97	0.99
Two siblings	0.97	0.96	0.98	0.97	0.96	0.97	0.97	0.97	0.98
Three siblings	0.99	0.98	1.00	0.98	0.97	0.99	0.98	0.97	0.99
Four or more children	1.04	1.03	1.05	1.00	0.99	1.01	1.00	0.99	1.01
Age (years)	1.07	1.07	1.07	1.08	1.07	1.08	1.06	1.06	1.06
Family income (ref. High)									
Low income				0.92	0.92	0.93	0.99	0.98	1.00
Middle–low income				1.02	1.01	1.03	1.05	1.04	1.06
Middle–high income				1.04	1.03	1.05	1.05	1.04	1.06
Educational level (ref. > 12 years)									
10–11 years				1.11	1.10	1.12	1.06	1.05	1.07
≥ 12 years				1.00	1.00	1.01	1.00	0.99	1.00
Socioeconomic status (ref. professionals)									
Farmers/self-employed/others				1.22	1.21	1.23	1.17	1.16	1.18
Blue collar workers				1.15	1.14	1.16	1.09	1.08	1.10
White collar workers				1.08	1.07	1.08	1.04	1.04	1.05
Region of residence (ref. Large cities)									
Southern Sweden				1.00	1.00	1.01	1.03	1.02	1.03
Northern Sweden				1.04	1.03	1.05	1.05	1.04	1.06
Immigrant status (ref. Born in Sweden)				0.95	0.93	0.97	0.96	0.95	0.98
Marital status (ref. Not married)				1.18	1.17	1.19	1.17	1.16	1.18
Birth order				1.01	1.01	1.01	1.01	1.01	1.02
Hospitalization of chronic lower respiratory disease (ref. Non)							1.26	1.25	1.28
Hospitalization of alcoholisms and related liver disease (ref. Non)							1.74	1.73	1.76
Hospitalization of diabetes (ref. Non)							1.39	1.38	1.40
Hospitalization of obesity (ref. Non)							1.37	1.35	1.40
Hospitalization of hypertension (ref. Non)							3.72	3.70	3.74
Cancer (ref. Non)							1.36	1.35	1.36

Model 1. Adjusted for age at start; Model 2. Adjusted for age at start and individual characteristics; Model 3. Model 2 + comorbidities.
*: Multivariable competing risk survival analysis

For peer review only

Supplementary Table 2b. Hazard ratio (HR) and 95% confidence interval of CVD in *men*, using multivariable competing risk survival analysis

	Model 1			Model 2			Model 3		
	HR*	95% CI		HR*	95% CI		HR*	95% CI	
Birth order (ref. First birth)									
Second	0.99	0.99	1.00	0.99	0.98	1.00	1.00	1.00	1.01
Third	1.02	1.01	1.03	1.01	1.00	1.02	1.02	1.02	1.03
Fourth	1.05	1.03	1.06	1.03	1.01	1.04	1.04	1.02	1.05
Fifth+	1.10	1.08	1.12	1.07	1.05	1.08	1.07	1.05	1.09
Age (years)	1.07	1.07	1.07	1.08	1.07	1.08	1.06	1.06	1.06
Family income (ref. High)									
Low income				0.92	0.92	0.93	0.99	0.98	1.00
Middle–low income				1.02	1.01	1.03	1.05	1.04	1.06
Middle–high income				1.04	1.03	1.05	1.05	1.04	1.06
Educational level (ref. > 12 years)									
10–11 years				1.11	1.10	1.12	1.06	1.05	1.07
≥ 12 years				1.00	1.00	1.01	1.00	0.99	1.00
Socioeconomic status (ref. professionals)									
Farmers/self-employed/others				1.22	1.21	1.23	1.17	1.16	1.18
Blue collar workers				1.15	1.14	1.16	1.09	1.08	1.10
White collar workers				1.08	1.07	1.08	1.04	1.04	1.05
Region of residence (ref. Large cities)									
Southern Sweden				1.00	1.00	1.01	1.03	1.02	1.03
Northern Sweden				1.04	1.03	1.05	1.05	1.04	1.06
Immigrant status (ref. Born in Sweden)				0.95	0.93	0.97	0.97	0.95	0.98
Marital status (ref. Not married)				1.18	1.17	1.19	1.17	1.16	1.18
Number of siblings				1.00	1.00	1.00	1.00	1.00	1.00
Hospitalization of chronic lower respiratory disease (ref. Non)							1.26	1.25	1.28
Hospitalization of alcoholisms and related liver disease (ref. Non)							1.74	1.73	1.76
Hospitalization of diabetes (ref. Non)							1.39	1.38	1.40
Hospitalization of obesity (ref. Non)							1.37	1.35	1.40
Hospitalization of hypertension (ref. Non)							3.72	3.70	3.74
Cancer (ref. Non)							1.36	1.35	1.36

Model 1. Adjusted for age at start; Model 2. Adjusted for age at start and individual characteristics; Model 3. Model 2 + comorbidities.
*: Multivariable competing risk survival analysis

For peer review only

Supplementary Table 2c. Hazard ratio (HR) and 95% confidence interval of CVD in *women*, using multivariable competing risk survival analysis

	Model 1			Model 2			Model 3		
	HR*	95% CI		HR*	95% CI		HR*	95% CI	
Number of siblings (ref. No sibling)									
One sibling	0.98	0.97	0.98	0.98	0.97	0.98	0.98	0.98	0.99
Two siblings	0.99	0.98	0.99	0.98	0.97	0.99	0.99	0.98	1.00
Three siblings	1.01	1.00	1.02	1.00	0.99	1.01	1.00	0.99	1.01
Four or more children	1.06	1.05	1.07	1.03	1.02	1.04	1.01	1.00	1.03
Age (years)	1.06	1.06	1.07	1.06	1.06	1.06	1.04	1.04	1.04
Family income (ref. High)									
Low income				0.87	0.86	0.87	0.95	0.94	0.96
Middle–low income				1.01	1.00	1.02	1.04	1.03	1.05
Middle–high income				1.02	1.01	1.03	1.03	1.02	1.03
Educational level (ref. > 12 years)									
10–11 years				1.13	1.12	1.14	1.08	1.06	1.09
≥ 12 years				1.04	1.03	1.05	1.03	1.02	1.04
Socioeconomic status (ref. professionals)									
Farmers/self-employed/others				1.30	1.28	1.31	1.20	1.19	1.22
Blue collar workers				1.25	1.24	1.27	1.12	1.11	1.13
White collar workers				1.08	1.07	1.10	1.03	1.02	1.04
Region of residence (ref. Large cities)									
Southern Sweden				1.03	1.03	1.04	1.03	1.03	1.04
Northern Sweden				1.06	1.06	1.07	1.04	1.03	1.05
Immigrant status (ref. Born in Sweden)				0.97	0.96	0.99	0.98	0.96	1.00
Marital status (ref. Not married)				1.13	1.12	1.14	1.10	1.09	1.11
Birth order				1.00	1.00	1.01	1.01	1.01	1.01
Hospitalization of chronic lower respiratory disease (ref. Non)							1.38	1.36	1.39
Hospitalization of alcoholisms and related liver disease (ref. Non)							1.67	1.65	1.69
Hospitalization of diabetes (ref. Non)							1.38	1.37	1.39
Hospitalization of obesity (ref. Non)							1.32	1.30	1.34
Hospitalization of hypertension (ref. Non)							4.86	4.83	4.89
Cancer (ref. Non)							1.62	1.61	1.63

Model 1. Adjusted for age at start; Model 2. Adjusted for age at start and individual characteristics; Model 3. Model 2 + comorbidities.
*: Multivariable competing risk survival analysis

For peer review only

Supplementary Table 2d. Hazard ratio (HR) and 95% confidence interval of CVD in *women*, using multivariable competing risk survival analysis

	Model 1			Model 2			Model 3		
	HR*	95% CI		HR*	95% CI		HR*	95% CI	
Birth order (ref. First birth)									
Second	1.00	0.99	1.01	0.99	0.98	1.00	1.01	1.00	1.02
Third	1.03	1.02	1.04	1.00	0.99	1.01	1.02	1.01	1.03
Fourth	1.06	1.04	1.07	1.01	1.00	1.03	1.04	1.02	1.05
Fifth+	1.09	1.07	1.10	1.01	0.99	1.03	1.05	1.03	1.07
Age (years)	1.07	1.07	1.07	1.06	1.06	1.06	1.04	1.04	1.04
Family income (ref. High)									
Low income				0.87	0.86	0.87	0.95	0.94	0.96
Middle–low income				1.01	1.00	1.02	1.04	1.03	1.05
Middle–high income				1.02	1.01	1.03	1.03	1.02	1.03
Educational level (ref. > 12 years)									
10–11 years				1.13	1.12	1.14	1.08	1.06	1.09
≥ 12 years				1.04	1.03	1.05	1.03	1.02	1.04
Socioeconomic status (ref. professionals)									
Farmers/self-employed/others				1.30	1.28	1.31	1.21	1.19	1.22
Blue collar workers				1.26	1.24	1.27	1.12	1.11	1.13
White collar workers				1.09	1.07	1.10	1.03	1.02	1.05
Region of residence (ref. Large cities)									
Southern Sweden				1.03	1.03	1.04	1.03	1.03	1.04
Northern Sweden				1.06	1.06	1.07	1.04	1.03	1.05
Immigrant status (ref. Born in Sweden)				0.97	0.96	0.99	0.98	0.96	1.00
Marital status (ref. Not married)				1.13	1.13	1.14	1.10	1.09	1.11
Number of siblings				1.01	1.01	1.01	1.00	1.00	1.01
Hospitalization of chronic lower respiratory disease (ref. Non)							1.38	1.36	1.39
Hospitalization of alcoholisms and related liver disease (ref. Non)							1.67	1.65	1.69
Hospitalization of diabetes (ref. Non)							1.38	1.37	1.39
Hospitalization of obesity (ref. Non)							1.32	1.30	1.34
Hospitalization of hypertension (ref. Non)							4.86	4.83	4.89
Cancer (ref. Non)							1.62	1.61	1.63

Model 1. Adjusted for age at start; Model 2. Adjusted for age at start and individual characteristics; Model 3. Model 2 + comorbidities.

*: Multivariable competing risk survival analysis

For peer review only

Supplementary Table 3a. Hazard ratio (HR) and 95% confidence interval of CHD in *men*

	Model 1			Model 2			Model 3		
	HR	95% CI		HR	95% CI		HR	95% CI	
Number of siblings (ref. No sibling)									
One sibling	1.00	0.98	1.02	0.99	0.97	1.01	0.99	0.97	1.01
Two siblings	1.04	1.03	1.06	1.00	0.98	1.02	1.01	0.99	1.03
Three siblings	1.12	1.10	1.14	1.04	1.02	1.06	1.04	1.02	1.07
Four or more children	1.27	1.25	1.30	1.10	1.08	1.12	1.10	1.07	1.12
Age (years)	1.08	1.08	1.08	1.08	1.08	1.08	1.07	1.07	1.07
Family income (ref. High)									
Low income				0.93	0.91	0.94	0.96	0.95	0.98
Middle–low income				1.00	0.98	1.01	1.02	1.00	1.03
Middle–high income				1.01	1.00	1.03	1.02	1.00	1.03
Educational level (ref. > 12 years)									
10–11 years				1.05	1.03	1.07	1.04	1.02	1.07
≥ 12 years				0.94	0.93	0.95	0.95	0.94	0.96
Socioeconomic status (ref. professionals)									
Farmers/self-employed/others				1.23	1.20	1.25	1.18	1.16	1.21
Blue collar workers				1.26	1.24	1.28	1.21	1.19	1.23
White collar workers				1.18	1.16	1.20	1.15	1.13	1.17
Region of residence (ref. Large cities)									
Southern Sweden				1.11	1.10	1.12	1.12	1.11	1.14
Northern Sweden				1.32	1.30	1.34	1.31	1.29	1.33
Immigrant status (ref. Born in Sweden)				1.09	1.05	1.13	1.10	1.06	1.14
Marital status (ref. Not married)				1.00	0.99	1.01	0.99	0.98	1.01
Birth order				1.05	1.04	1.05	1.05	1.05	1.06
Hospitalization of chronic lower respiratory disease (ref. Non)							1.29	1.26	1.31
Hospitalization of alcoholisms and related liver disease (ref. Non)							1.01	0.99	1.04
Hospitalization of diabetes (ref. Non)							1.75	1.73	1.78
Hospitalization of obesity (ref. Non)							1.13	1.09	1.17
Hospitalization of hypertension (ref. Non)							1.86	1.84	1.88
Cancer (ref. Non)							0.91	0.89	0.92

Model 1. Adjusted for age at start; Model 2. Adjusted for age at start and individual characteristics; Model 3. Model 2 + comorbidities.

For peer review only

Supplementary Table 3b. Hazard ratio (HR) and 95% confidence interval of CHD in *men*

	Model 1			Model 2			Model 3		
	HR	95% CI		HR	95% CI		HR	95% CI	
Birth order (ref. First birth)									
Second	1.09	1.08	1.10	1.06	1.05	1.07	1.08	1.06	1.09
Third	1.19	1.17	1.21	1.11	1.09	1.13	1.13	1.11	1.15
Fourth	1.28	1.25	1.32	1.15	1.12	1.19	1.17	1.14	1.21
Fifth+	1.44	1.39	1.48	1.22	1.17	1.26	1.23	1.19	1.28
Age (years)	1.08	1.08	1.09	1.08	1.08	1.08	1.07	1.07	1.07
Family income (ref. High)									
Low income				0.93	0.91	0.94	0.96	0.95	0.98
Middle–low income				1.00	0.98	1.01	1.02	1.00	1.03
Middle–high income				1.01	1.00	1.02	1.02	1.00	1.03
Educational level (ref. > 12 years)									
10–11 years				1.05	1.03	1.08	1.05	1.03	1.07
≥ 12 years				0.94	0.93	0.95	0.95	0.94	0.97
Socioeconomic status (ref. professionals)									
Farmers/self-employed/others				1.23	1.20	1.25	1.18	1.16	1.21
Blue collar workers				1.26	1.24	1.28	1.21	1.19	1.23
White collar workers				1.18	1.16	1.20	1.15	1.13	1.17
Region of residence (ref. Large cities)									
Southern Sweden				1.11	1.10	1.13	1.12	1.11	1.14
Northern Sweden				1.32	1.30	1.34	1.31	1.29	1.33
Immigrant status (ref. Born in Sweden)				1.09	1.05	1.13	1.10	1.06	1.14
Marital status (ref. Not married)				1.00	0.99	1.01	0.99	0.98	1.01
Number of siblings				1.02	1.02	1.02	1.02	1.02	1.02
Hospitalization of chronic lower respiratory disease (ref. Non)							1.29	1.26	1.31
Hospitalization of alcoholisms and related liver disease (ref. Non)							1.01	0.99	1.04
Hospitalization of diabetes (ref. Non)							1.75	1.73	1.78
Hospitalization of obesity (ref. Non)							1.13	1.09	1.17
Hospitalization of hypertension (ref. Non)							1.86	1.84	1.88
Cancer (ref. Non)							0.91	0.89	0.92

Model 1. Adjusted for age at start; Model 2. Adjusted for age at start and individual characteristics; Model 3. Model 2 + comorbidities.

For peer review only

Supplementary Table 3c. Hazard ratio (HR) and 95% confidence interval of CHD in *women*

	Model 1			Model 2			Model 3		
	HR	95% CI		HR	95% CI		HR	95% CI	
Number of siblings (ref. No sibling)									
One sibling	0.99	0.97	1.02	0.98	0.96	1.01	0.99	0.97	1.02
Two siblings	1.07	1.04	1.10	1.03	1.00	1.05	1.03	1.00	1.06
Three siblings	1.17	1.13	1.20	1.08	1.04	1.11	1.07	1.04	1.11
Four or more children	1.40	1.36	1.44	1.20	1.16	1.24	1.17	1.13	1.21
Age (years)	1.09	1.09	1.09	1.09	1.08	1.09	1.07	1.07	1.07
Family income (ref. High)									
Low income				0.85	0.83	0.88	0.91	0.88	0.93
Middle–low income				1.00	0.98	1.03	1.00	0.98	1.03
Middle–high income				1.02	1.00	1.05	1.02	0.99	1.04
Educational level (ref. > 12 years)									
10–11 years				1.07	1.04	1.10	1.06	1.03	1.09
≥ 12 years				0.92	0.90	0.94	0.94	0.92	0.96
Socioeconomic status (ref. professionals)									
Farmers/self-employed/others				1.52	1.46	1.59	1.37	1.31	1.42
Blue collar workers				1.47	1.42	1.53	1.33	1.28	1.38
White collar workers				1.19	1.14	1.23	1.14	1.10	1.18
Region of residence (ref. Large cities)									
Southern Sweden				1.14	1.12	1.16	1.15	1.13	1.17
Northern Sweden				1.40	1.37	1.44	1.38	1.34	1.41
Immigrant status (ref. Born in Sweden)				1.13	1.07	1.20	1.13	1.07	1.20
Marital status (ref. Not married)				1.12	1.10	1.14	1.08	1.06	1.10
Birth order				1.04	1.03	1.05	1.05	1.04	1.06
Hospitalization of chronic lower respiratory disease (ref. Non)							1.69	1.65	1.73
Hospitalization of alcoholisms and related liver disease (ref. Non)							1.17	1.11	1.23
Hospitalization of diabetes (ref. Non)							2.20	2.15	2.25
Hospitalization of obesity (ref. Non)							1.11	1.06	1.17
Hospitalization of hypertension (ref. Non)							2.22	2.18	2.26
Cancer (ref. Non)							0.94	0.93	0.96

Model 1. Adjusted for age at start; Model 2. Adjusted for age at start and individual characteristics; Model 3. Model 2 + comorbidities.

For peer review only

Supplementary Table 3d. Hazard ratio (HR) and 95% confidence interval of CHD in *women*

	Model 1			Model 2			Model 3		
	HR	95% CI		HR	95% CI		HR	95% CI	
Birth order (ref. First birth)									
Second	1.10	1.07	1.12	1.05	1.02	1.07	1.07	1.05	1.09
Third	1.24	1.21	1.28	1.11	1.08	1.14	1.14	1.11	1.18
Fourth	1.33	1.27	1.38	1.11	1.06	1.16	1.14	1.09	1.20
Fifth+	1.56	1.48	1.63	1.17	1.11	1.24	1.22	1.15	1.29
Age (years)	1.09	1.09	1.09	1.09	1.08	1.09	1.07	1.07	1.07
Family income (ref. High)									
Low income				0.85	0.83	0.88	0.91	0.88	0.93
Middle–low income				1.00	0.98	1.03	1.01	0.98	1.03
Middle–high income				1.02	1.00	1.05	1.02	0.99	1.04
Educational level (ref. > 12 years)									
10–11 years				1.07	1.04	1.10	1.06	1.03	1.09
≥ 12 years				0.92	0.90	0.94	0.94	0.92	0.96
Socioeconomic status (ref. professionals)									
Farmers/self-employed/others				1.52	1.46	1.59	1.37	1.31	1.42
Blue collar workers				1.47	1.42	1.53	1.33	1.28	1.38
White collar workers				1.19	1.14	1.23	1.14	1.10	1.18
Region of residence (ref. Large cities)									
Southern Sweden				1.14	1.12	1.17	1.15	1.13	1.17
Northern Sweden				1.40	1.37	1.44	1.38	1.34	1.41
Immigrant status (ref. Born in Sweden)				1.13	1.07	1.20	1.13	1.07	1.20
Marital status (ref. Not married)				1.12	1.10	1.14	1.08	1.06	1.10
Number of siblings				1.04	1.03	1.04	1.03	1.03	1.04
Hospitalization of chronic lower respiratory disease (ref. Non)							1.69	1.65	1.73
Hospitalization of alcoholisms and related liver disease (ref. Non)							1.17	1.11	1.23
Hospitalization of diabetes (ref. Non)							2.20	2.15	2.25
Hospitalization of obesity (ref. Non)							1.11	1.06	1.17
Hospitalization of hypertension (ref. Non)							2.22	2.18	2.26
Cancer (ref. Non)							0.94	0.93	0.96

Model 1. Adjusted for age at start; Model 2. Adjusted for age at start and individual characteristics; Model 3. Model 2 + comorbidities.

For peer review only

Supplementary Table 4a. Hazard ratio (HR) and 95% confidence interval of mortality in *men*

	Model 1			Model 2			Model 3		
	HR	95% CI		HR	95% CI		HR	95% CI	
Number of siblings (ref. No sibling)									
One sibling	0.91	0.90	0.92	0.92	0.91	0.93	0.93	0.92	0.94
Two siblings	0.92	0.91	0.93	0.91	0.90	0.92	0.91	0.90	0.92
Three siblings	0.96	0.94	0.97	0.93	0.92	0.94	0.93	0.92	0.94
Four or more children	1.03	1.01	1.04	0.96	0.94	0.97	0.96	0.94	0.97
Age (years)	1.10	1.10	1.10	1.11	1.11	1.11	1.10	1.10	1.10
Family income (ref. High)									
Low income				1.05	1.04	1.07	1.08	1.07	1.10
Middle–low income				1.17	1.15	1.18	1.14	1.13	1.15
Middle–high income				1.18	1.17	1.19	1.15	1.14	1.16
Educational level (ref. > 12 years)									
10–11 years				1.12	1.10	1.13	1.08	1.06	1.09
≥ 12 years				0.91	0.90	0.91	0.89	0.88	0.90
Socioeconomic status (ref. professionals)									
Farmers/self-employed/others				1.73	1.71	1.76	1.63	1.60	1.65
Blue collar workers				1.38	1.36	1.40	1.34	1.32	1.36
White collar workers				1.15	1.13	1.17	1.14	1.12	1.16
Region of residence (ref. Large cities)									
Southern Sweden				0.94	0.93	0.95	0.99	0.98	1.00
Northern Sweden				0.98	0.97	0.99	1.08	1.07	1.09
Immigrant status (ref. Born in Sweden)				1.04	1.01	1.07	1.06	1.03	1.09
Marital status (ref. Not married)				1.88	1.87	1.90	1.71	1.70	1.73
Birth order				1.00	1.00	1.01	0.99	0.99	1.00
Hospitalization of chronic lower respiratory disease (ref. Non)							1.28	1.26	1.30
Hospitalization of alcoholisms and related liver disease (ref. Non)							3.02	2.98	3.05
Hospitalization of diabetes (ref. Non)							1.43	1.41	1.45
Hospitalization of obesity (ref. Non)							1.09	1.06	1.13
Hospitalization of hypertension (ref. Non)							0.66	0.65	0.67
Cancer (ref. Non)							2.62	2.60	2.65

Model 1. Adjusted for age at start; Model 2. Adjusted for age at start and individual characteristics; Model 3. Model 2 + comorbidities.

For peer review only

Supplementary Table 4b. Hazard ratio (HR) and 95% confidence interval of mortality in *men*

	Model 1			Model 2			Model 3		
	HR	95% CI		HR	95% CI		HR	95% CI	
Birth order (ref. First birth)									
Second	0.98	0.97	0.99	0.97	0.96	0.98	0.96	0.95	0.97
Third	1.01	0.99	1.02	0.99	0.98	1.01	0.98	0.96	0.99
Fourth	1.04	1.01	1.06	1.00	0.98	1.03	0.98	0.95	1.00
Fifth+	1.11	1.08	1.14	1.06	1.02	1.09	1.01	0.98	1.05
Age (years)	1.10	1.10	1.10	1.11	1.11	1.11	1.10	1.10	1.10
Family income (ref. High)									
Low income				1.06	1.04	1.07	1.08	1.07	1.10
Middle–low income				1.17	1.15	1.18	1.14	1.13	1.16
Middle–high income				1.18	1.17	1.19	1.15	1.14	1.16
Educational level (ref. > 12 years)									
10–11 years				1.12	1.10	1.13	1.07	1.06	1.09
≥ 12 years				0.90	0.90	0.91	0.89	0.88	0.90
Socioeconomic status (ref. professionals)									
Farmers/self-employed/others				1.73	1.71	1.76	1.63	1.60	1.66
Blue collar workers				1.38	1.36	1.40	1.34	1.32	1.36
White collar workers				1.15	1.13	1.17	1.14	1.13	1.16
Region of residence (ref. Large cities)									
Southern Sweden				0.94	0.93	0.95	0.99	0.98	1.00
Northern Sweden				0.98	0.97	0.99	1.08	1.07	1.09
Immigrant status (ref. Born in Sweden)				1.05	1.02	1.08	1.06	1.03	1.09
Marital status (ref. Not married)				1.88	1.87	1.90	1.71	1.70	1.73
Number of siblings				1.00	0.99	1.00	1.00	0.99	1.00
Hospitalization of chronic lower respiratory disease (ref. Non)							1.28	1.26	1.30
Hospitalization of alcoholisms and related liver disease (ref. Non)							3.02	2.98	3.05
Hospitalization of diabetes (ref. Non)							1.43	1.41	1.45
Hospitalization of obesity (ref. Non)							1.09	1.06	1.13
Hospitalization of hypertension (ref. Non)							0.66	0.65	0.67
Cancer (ref. Non)							2.62	2.60	2.64

Model 1. Adjusted for age at start; Model 2. Adjusted for age at start and individual characteristics; Model 3. Model 2 + comorbidities.

For peer review only

Supplementary Table 4c. Hazard ratio (HR) and 95% confidence interval of mortality in *women*

	Model 1			Model 2			Model 3		
	HR	95% CI		HR	95% CI		HR	95% CI	
Number of siblings (ref. No sibling)									
One sibling	0.92	0.91	0.94	0.93	0.92	0.95	0.94	0.93	0.95
Two siblings	0.92	0.91	0.94	0.91	0.90	0.93	0.92	0.91	0.94
Three siblings	0.95	0.93	0.96	0.92	0.90	0.93	0.93	0.91	0.95
Four or more children	1.01	0.99	1.03	0.94	0.92	0.95	0.95	0.93	0.96
Age (years)	1.10	1.10	1.10	1.10	1.10	1.10	1.09	1.08	1.09
Family income (ref. High)									
Low income				0.84	0.83	0.86	0.94	0.93	0.96
Middle–low income				1.03	1.01	1.05	1.06	1.04	1.07
Middle–high income				1.05	1.04	1.06	1.05	1.04	1.07
Educational level (ref. > 12 years)									
10–11 years				1.08	1.06	1.10	1.01	1.00	1.03
≥ 12 years				0.85	0.84	0.87	0.82	0.81	0.83
Socioeconomic status (ref. professionals)									
Farmers/self-employed/others				1.97	1.93	2.02	1.88	1.84	1.93
Blue collar workers				1.36	1.33	1.39	1.35	1.32	1.38
White collar workers				1.12	1.10	1.15	1.11	1.09	1.14
Region of residence (ref. Large cities)									
Southern Sweden				1.00	0.99	1.01	1.03	1.02	1.04
Northern Sweden				1.03	1.02	1.05	1.11	1.09	1.12
Immigrant status (ref. Born in Sweden)				0.89	0.86	0.92	0.95	0.92	0.99
Marital status (ref. Not married)				1.63	1.62	1.65	1.48	1.47	1.50
Birth order				1.01	1.00	1.01	1.00	0.99	1.01
Hospitalization of chronic lower respiratory disease (ref. Non)							1.61	1.58	1.63
Hospitalization of alcoholisms and related liver disease (ref. Non)							3.26	3.20	3.32
Hospitalization of diabetes (ref. Non)							1.58	1.56	1.61
Hospitalization of obesity (ref. Non)							0.96	0.93	0.99
Hospitalization of hypertension (ref. Non)							0.66	0.65	0.67
Cancer (ref. Non)							4.86	4.81	4.91

Model 1. Adjusted for age at start; Model 2. Adjusted for age at start and individual characteristics; Model 3. Model 2 + comorbidities.

For peer review only

Supplementary Table 4d. Hazard ratio (HR) and 95% confidence interval of mortality in *women*

	Model 1			Model 2			Model 3		
	HR	95% CI		HR	95% CI		HR	95% CI	
Birth order (ref. First birth)									
Second	0.98	0.96	0.99	0.97	0.96	0.98	0.96	0.95	0.98
Third	1.01	1.00	1.03	1.00	0.98	1.02	0.98	0.96	1.00
Fourth	1.05	1.02	1.08	1.02	0.99	1.05	1.00	0.98	1.03
Fifth+	1.11	1.08	1.15	1.08	1.04	1.12	1.03	0.99	1.07
Age (years)	1.10	1.10	1.10	1.10	1.10	1.10	1.09	1.08	1.09
Family income (ref. High)									
Low income				0.84	0.83	0.86	0.94	0.93	0.96
Middle–low income				1.03	1.02	1.05	1.06	1.04	1.07
Middle–high income				1.05	1.04	1.07	1.05	1.04	1.07
Educational level (ref. > 12 years)									
10–11 years				1.08	1.06	1.10	1.01	0.99	1.03
≥ 12 years				0.85	0.84	0.86	0.82	0.81	0.83
Socioeconomic status (ref. professionals)									
Farmers/self-employed/others				1.98	1.93	2.02	1.88	1.84	1.93
Blue collar workers				1.36	1.33	1.39	1.35	1.32	1.38
White collar workers				1.12	1.10	1.15	1.11	1.09	1.14
Region of residence (ref. Large cities)									
Southern Sweden				1.00	0.99	1.01	1.03	1.02	1.04
Northern Sweden				1.03	1.02	1.05	1.11	1.09	1.12
Immigrant status (ref. Born in Sweden)				0.89	0.86	0.92	0.95	0.92	0.99
Marital status (ref. Not married)				1.63	1.62	1.65	1.48	1.47	1.50
Number of siblings				0.99	0.99	0.99	0.99	0.99	1.00
Hospitalization of chronic lower respiratory disease (ref. Non)							1.61	1.58	1.63
Hospitalization of alcoholisms and related liver disease (ref. Non)							3.26	3.20	3.33
Hospitalization of diabetes (ref. Non)							1.58	1.56	1.61
Hospitalization of obesity (ref. Non)							0.96	0.93	0.99
Hospitalization of hypertension (ref. Non)							0.66	0.65	0.67
Cancer (ref. Non)							4.86	4.81	4.91

Model 1. Adjusted for age at start; Model 2. Adjusted for age at start and individual characteristics; Model 3. Model 2 + comorbidities.

For peer review only

BMJ Open

Sibling rank and sibling number in relation to cardiovascular disease and mortality risk: a nationwide cohort study

Journal:	*BMJ Open*
Manuscript ID	bmjopen-2020-042881.R2
Article Type:	Original research
Date Submitted by the Author:	27-Mar-2021
Complete List of Authors:	Nilsson, Peter; Lunds Universitet, Clinical Sciences<br>Sundquist, Jan; Lund University, Center for Primary Health Care Research;<br>Sundquist, Kristina; Lund University, Center for Primary Health Care Research<br>Li, Xinjun; Lund University, Center for Primary Health Care Research
**Primary Subject Heading**:	Epidemiology
Secondary Subject Heading:	Cardiovascular medicine
Keywords:	EPIDEMIOLOGY, REPRODUCTIVE MEDICINE, SOCIAL MEDICINE

SCHOLARONE™
Manuscripts

I, the Submitting Author has the right to grant and does grant on behalf of all authors of the Work (as defined in the below author licence), an exclusive licence and/or a non-exclusive licence for contributions from authors who are: i) UK Crown employees; ii) where BMJ has agreed a CC-BY licence shall apply, and/or iii) in accordance with the terms applicable for US Federal Government officers or employees acting as part of their official duties; on a worldwide, perpetual, irrevocable, royalty-free basis to BMJ Publishing Group Ltd ("BMJ") its licensees and where the relevant Journal is co-owned by BMJ to the co-owners of the Journal, to publish the Work in this journal and any other BMJ products and to exploit all rights, as set out in our licence.

The Submitting Author accepts and understands that any supply made under these terms is made by BMJ to the Submitting Author unless you are acting as an employee on behalf of your employer or a postgraduate student of an affiliated institution which is paying any applicable article publishing charge ("APC") for Open Access articles. Where the Submitting Author wishes to make the Work available on an Open Access basis (and intends to pay the relevant APC), the terms of reuse of such Open Access shall be governed by a Creative Commons licence – details of these licences and which Creative Commons licence will apply to this Work are set out in our licence referred to above.

Other than as permitted in any relevant BMJ Author's Self Archiving Policies, I confirm this Work has not been accepted for publication elsewhere, is not being considered for publication elsewhere and does not duplicate material already published. I confirm all authors consent to publication of this Work and authorise the granting of this licence.

For peer review only

Original manuscript for the BMJ OPEN 2021 03 27/PN

Revised Version # 4.

Sibling rank and sibling number in relation to cardiovascular disease and mortality risk: a nationwide cohort study

Peter M Nilsson [1], Jan Sundquist [1,2], Kristina Sundquist [1,2] and Xinjun Li [1]

[1] Department of Clinical Sciences, Lund University, Skåne University Hospital, Malmö, Sweden

[2] Center for Primary Care Research, Lund University, Skåne University Hospital, Malmö, Sweden

For peer review only

Short title: Sibling rank, CVD and mortality risk

Number of words: 247 in the Abstract, 2160 in full text

Number of references: 26

Number of tables: 3 + 3 supplemental

Number of figures: 3 + 1 supplemental

Conflict of interest: None

Corresponding author:

Peter M Nilsson, MD, PhD

Lund University, Department of Clinical Sciences,

Skåne University Hospital,

Jan Waldenströms gata 15, 5th floor

S-205 02 Malmö, Sweden.

Email: Peter.Nilsson@med.lu.se

Phone: +46-40-33 24 15

For peer review only - http://bmjopen.bmj.com/site/about/guidelines.xhtml

Abstract

Background: The number and rank order of siblings could be of importance for risk of cardiovascular disease and mortality. Previous studies have used only fatal events for risk prediction. We therefore aimed to use also non-fatal coronary and cardiovascular events in fully adjusted models.

Methods: From the Multi-Generation Register in Sweden data were used from 1.36 million men and 1.32 million women (born 1932 to 1960), aged 30-58 years at baseline and with follow-up from 1990 to 2015. Mean age at follow-up was 67 years (range 55-83 years). Fatal and non-fatal events were retrieved from national registers.

Results:

Compared to men with no siblings, those with 1-2 siblings had a lower, and those with 4 or more siblings had a higher adjusted risk of cardiovascular events. Again, compared to men with no siblings, those with more than 1 sibling had a lower total mortality risk, and those with 3 or more siblings had an increased risk of coronary events.

Correspondingly, compared to women with no siblings those women with 3 siblings or more had an increased risk of cardiovascular events, and those with 2 siblings or more had an increased risk of coronary events. Women with 1 sibling or more were at lower total mortality risk, following full adjustment.

Conclusion: Being first-born is associated with a favourable effect on non-fatal cardiovascular and coronary events for both men and women. The underlying biological mechanisms for this should be studied in a socio-cultural context.

Key words: Cardiovascular, Coronary, Epidemiology, Mortality, Register, Risk, Sibling

For peer review only

Strengths and limitations of the study

- This national register linkage study across generations includes data on both fatal and, for the first time, non-fatal cardiovascular and coronary events, as well as total mortality in relation to sibling number and rank
- Adjustment has been made for confounders including markers of social background (educational level, occupation) of the individual, but not for parental socioeconomic status. Competing risk analyses have been applied
- Limitations of the study include the lack of data from primary health care visits and that the historical register data do not fully reflect the ethnical diversity of Sweden today

For peer review only

For peer review only - http://bmjopen.bmj.com/site/about/guidelines.xhtml

Introduction

A positive family history of disease is a well-established variable to be used in risk algorithms for cardiovascular disease (CVD), even if it may be hard to quantify based on subjective recall only [1,2]. An alternative is to use register-based data for a more objective appraisal of the family burden of CVD [3]. One special feature of family structure is the *number of siblings* and *sibling rank* that can also be mapped by use of national registers such as the Multiple-Generation Register (MGR) of Sweden [4]. So far this register has been mostly used to describe the risk of some selected CVD manifestations of individuals in relation to the risk of their siblings, for example for thromboembolic disease [5] or other diseases [6-9].

In a previous report based on the MGR it was shown that increased number of sibling reflecting family size was not associated with increased total and cause-specific mortality risk in ages 40-74 years, but no analyses were made for risk of non-fatal CVD or coronary disease [10]. However, another corresponding study based on MGR data could show that total and cause-specific mortality in ages 30-69 years increased with increasing birth order [11].

The influence of sibling rank has been less well studied in relation to non-fatal cardiovascular and coronary risk. Previous studies have indicated a worse cardiovascular risk factor burden in first-borns, for example increased body mass index (BMI) and systolic blood pressure, but lower insulin sensitivity, than later born siblings [12-16]. On the other hand, first-borns seem to have a better physical fitness at military conscript testing [17], less caries [18] and run a lower risk of leukemia in adolescence [19] as well as lower suicide risk in a Finnish population [20].

Against this background we *aimed* to analyse the influence of sibling number and rank on risk of non-fatal and fatal cardiovascular and coronary manifestations, as well as total mortality, in the MGR of Sweden, after extensive adjustment for background demographic factors and family social status.

Subjects and methods

Setting and participants

The dataset used in this study was constructed by linking several national Swedish registers. The Swedish government-owned Statistics Sweden provided the Multi-Generation Register (MGR), in which persons born in Sweden in or after 1932 (the present study population) are linked to their parents [4]. We included all births (multiple births, full and half siblings) in the analyses. Linkages were made to National Census data in order to ascertain individual-level socioeconomic status. The final link was made by adding data from the Swedish Cause of Death Register (1961-2015) and the Swedish Hospital Discharge Register, with recorded dates of hospitalization and hospital diagnoses

For peer review only

since 1964, but on a national level since 1987, and now until end of 2015. National Swedish registers are of high validity for medical research [21, 22]. For analysing risk of CVD, CHD and mortality in relation to number of siblings and birth order, we collected data from 1.36 million men and 1.32 million women (born 1932 to 1960), aged 30-58 years at baseline and with follow-up from 1990 to 2015. For the definition of study subjects based on the MGR, see supplemental **Figure S1**.

Patient and public involvement

No patient involved.

Follow-up of CVD events and total mortality

We used the following ICD-codes for fatal or non-fatal CVD (ICD-9, 390-459, ICD-10, I00-I99); and for CHD (ICD-9, 410-414, ICD-10, I20-I25). Non-fatal events were followed in the national Hospital Discharge Register, and fatal events and total mortality until 31st December 2015 in the national Mortality Register.

Definitions

Family income: Family income was calculated at start of follow-up (1990) as annual family income divided by the number of members in the family. The income calculation was weighted, taking the ages of the family members into account. For example, children were given lower consumption weights than adults. The calculation was performed as follows: the sum of all family members' incomes was multiplied by the individual's consumption weight divided by the family members' total consumption weight. The final variable was calculated as empirical quartiles from the distribution and classified as low, middle-low, middle-high, and high.

Immigration status: born in Sweden or in other countries

Marital status: individuals were classified as married/cohabitating or never married, widowed, or divorced.

Socioeconomic status: was divided into four categories: the self-employed/farmers/all others, blue collar workers, white collar workers, or professionals

Education: was based on educational level, which was classified into three categories: ≤ 9 years, 10–11 years, and ≥ 12 years.

Geographic region: was divided into large cities (cities with a population of more than 200,000 inhabitants), Southern Sweden, and Northern Sweden.

Co-morbidity: was defined as the first hospitalization during the follow-up period of: chronic obstructive pulmonary disease, COPD (both hospitalization and mortality were included) (ICD-9 490–

For peer review only - http://bmjopen.bmj.com/site/about/guidelines.xhtml

496 and ICD-10 J40-J49), obesity (ICD-9 278A and ICD-10 E65-E68), alcoholism related liver disease (both hospitalizations and mortality were included) (ICD-9 291, 303, 571 and ICD-10 F10 and K70), hypertension (ICD-9 401-405 and ICD-10 I10–I15), and diabetes (both hospitalizations and mortality were included) (ICD-9 250 and ICD-10 E10-E14), and cancer (cancer were included both from cancer register and mortality, ICD-9 140-239, and ICD-10 C00-D48).

Statistical methods

Person-years at risk were calculated from the start of follow-up on 1 January 1990 until hospitalization or death from CVD, death from other causes, emigration, or the end of the follow-up, 31st December 2015. Age-adjusted incidence rates for first hospitalization and mortality were calculated for the entire follow-up period. We used the Cox's proportional hazard model to calculate the hazard ratio (HR) with 95 percent confidence intervals (CI) for total (fatal and non-fatal) CVD and CHD event risk, and for total mortality, for both men and women) in relation to number siblings and birth order. This was done after adjustment for age at start, individual characteristics (family income, marital status, immigrant background and educational level, region of residence, socioeconomic status), and finally for co-morbidities in order to adjust for competing mortality risk. Individuals without sibling was used as the reference. The proportionality assumptions were checked by plotting the incidence rates over time and by calculating Schoenfeld (partial) residuals and these assumptions were fulfilled. We used SAS version 9.4 (SAS Institute Inc. Cary, NC, USA) for all statistical analyses.

A further adjustment was made for total number of siblings in relation to birth order when the risk for different outcomes was calculated, using the category "first birth" as reference. A competing risk model used for mortality as a competing risk for incident CVD. A p-value < 0.05 was considered significant.

Results

With an average of 20 years (Q1-Q3 16-25 years) follow-up, in 1,358,647 men we used data on 592,863 CVD events, 131,533 coronary events, and 240,371 total deaths. For 1,315,037 women, the corresponding numbers were 486,147 CVD events, 55,933 coronary events and 160,269 deaths, respectively. The mean age of the study population at the end of the follow-up was 67 years (range 55-83 years). The number of siblings and birth order of men and women are depicted in **Table 1**.

Risk associated with number of siblings in men and women

Compared to men with no siblings, those with 1-2 siblings had a lower, and those with 4 or more siblings had a higher risk of cardiovascular events. Again, compared to men with no siblings, those

For peer review only

with more than 1 sibling had a lower total mortality risk, and those with 3 or more siblings had an increased risk of coronary events, following full adjustment, **Table 2.**

Correspondingly, compared to women with no siblings those women with 3 siblings or more had an increased risk of cardiovascular events, and those with 2 siblings or more had an increased risk of coronary events. Women with 1 sibling or more were at lower total mortality risk, following full adjustment, **Table 3.**

Risk associated with sibling rank in men and women

According to sibling rank, first-born men had a lower risk of both cardiovascular and coronary events than their later-born siblings, but higher total mortality than second and third-born siblings, following full adjustment, **Table 2**.

For first-born women the risks of cardiovascular and coronary events were also lower than in their later-born siblings. The mortality risk was higher than for second-born siblings, but equal to higher numbered siblings, following full adjustment, **Table 3**.

For hazard ratios of CHD, CVD and total mortality by number of siblings and birth order in men and women, respectively, see **Figures 1-3.**

Supplemental material

For detailed data on the distribution of the study population, number of CVD, coronary heart disease (CHD), and mortality events in men and women, see Supplemental **Table S1**. For detailed data on the risk associated with factors adjusted for, see Supplemental **Tables S2-S4,** for men and women, respectively.

Discussion

In this very large observational study based on a national Multi-Generation Register it was found that first-born men and women are at lower risk of both cardiovascular and coronary events than their later-born siblings, but had higher total mortality risk than second and third-born siblings (men). For women the mortality risk for first-born women was higher than for second-born siblings, following full adjustment for a number of background factors.

For total mortality in relation to sibling number our data are at odds with a previous study using the same register in Sweden, showing no increased mortality associated until 74 years with a higher number of siblings [10]. However, we used higher numbers, longer follow-up and more extensive adjustment.

For peer review only - http://bmjopen.bmj.com/site/about/guidelines.xhtml

For the influence of sibling rank, a previous study could show higher risk of total and cause-specific mortality with increasing sibling rank until 69 years [11]. This was similar in our study for risk of non-fatal cardiovascular and coronary events during longer follow-up and extensive adjustment.

These findings of lower cardiovascular risk in first-borns are at contrast to previous reports of a higher level of cardiovascular risk factors in such individuals followed until adolescence or young adulthood [12-16]. The burden of risk factors might have been compensated for by a better physical fitness, as noticed in first-born men coming for military conscript testing at the age of around 18 years [17]. In contrast to these observations, our extensive data indicate a lower cardiovascular risk in first-borns. Other unmeasured factors linked to being first-born, such as cognition or bodily development, could have contributed to our findings of a relative protection, even if we adjusted for a long list of potential confounders such as educational level, socio-economic status, marital status, and co-morbidities.

Besides filling a knowledge gap, this is of public health interest as different countries endorse different policies to support families and number of children. Our findings relate to family size and the biological as well as social roles related to sibling rank with its health implications.

More research is needed to understand the links between sibling number and rank with health outcomes. This could address, for example, the dilution of resources theory [23] of special relevance for disadvantaged girls; epigenetic factors influencing the metabolic syndrome in offspring [24]; and maternal health during pregnancy, including the effects of multiple births/child-rearing on maternal health and family resources, especially in deprived settings with large families [25].

Limitations and strengths of the study

The Swedish hospital discharge register contains no information about diagnostic procedures, which is a limitation. Moreover, specialist doctors in hospital care made the diagnosis. Another limitation is that we had no data on life-style related factors such as body mass index, smoking, and diet, because it would be unrealistic to gather such data for an entire national population. However, we did adjust for socioeconomic status, obesity, diabetes, COPD, and alcoholism and related liver disorders, which are associated with factors such as smoking and alcohol. Given a focus on family size, knowing that siblings who died young, and therefore not contributed to resource dilution for a proportion of the index person's childhood, would be of interest and importance. It would also shed light (potentially) on family circumstances and health. Regretfully, we currently lack data on parental SES to adjust for.

Strengths of the study include complete nationwide coverage from 1990 in a country with high standards of diagnosis, and with diagnoses often being made by specialists during extended examinations in clinics. Another important strength of our study is that it was based on nationwide registers and was thus free of selection and recall bias. The Swedish MGR and the Swedish Hospital

For peer review only - http://bmjopen.bmj.com/site/about/guidelines.xhtml

Discharge Register are validated data sources that have been proven to be reliable in the study of many diseases [4, 21,22]. Data in our dataset are almost 100% complete [4]. Generalisability (external validity) should hold at least for countries and populations similar to Sweden.

Future research should be directed to find biological or social mechanisms linking the status of being first-born to lower risk of cardiovascular disease, as indicated by our observational findings. A previous Norwegian study in military conscripts indicated that the role of being first-born is influenced by social factors, as a second-born son may achieve characteristics of a first-born brother who died young [26].

In conclusion, our data indicate a favourable effect on non-fatal cardiovascular and coronary events by being first-born, both for men and women.

a. Contributorship statement

PMN provided the original idea, and XL made the statistical analyses. PMN and XL drafted the first manuscript. All authors (PMN, XL, JS, and KS) contributed to the final manuscript. PMN and XL made the revisions.

b. Competing interests

None

c. Funding

This work was supported by the grants to Kristina Sundquist from The Swedish Research Council; ALF funding from Region Skåne and the Swedish Heart-Lung foundation.

d. Data sharing statement

Data will not be shared, but reasonable requests may be directed to Professor Kristina Sundquist, the PI of the registers.

e. Ethics statement

The study was approved by the Regional Ethics Review Board at the Lund University, Sweden (ID number 2012/795; approved 2013-02-06).

For peer review only - http://bmjopen.bmj.com/site/about/guidelines.xhtml

References

1. Banerjee A. A review of family history of cardiovascular disease: risk factor and research tool. *Int J Clin Pract*. 2012; 66:536-43.

2. Tada H, Melander O, Louie JZ, *et al.* Risk prediction by genetic risk scores for coronary heart disease is independent of self-reported family history. *Eur Heart J*. 2016;37(6):561–567.

3. Nilsson PM, Nilsson J-Å, and Berglund G. Family burden of cardiovascular mortality – risk implications for offspring in a national register linkage study based upon the Malmö Preventive Project. *J Internal Med* 2004; 255:229-35.

4. Ekbom A. The Swedish Multi-generation Register. *Methods Mol Biol*. 2011;675:215–220.

5. Zöller B, Ji J, Sundquist J, Sundquist K. Body Height and Incident Risk of Venous Thromboembolism: A Cosibling Design. *Circ Cardiovasc Genet.* 2017;10(5):e001651.

6. Kasiman K, Lundholm C, Sandin S, Malki N, Sparén P, Ingelsson E. Familial effects on ischemic stroke: the role of sibling kinship, sex, and age of onset. *Circ Cardiovasc Genet.* 2012;5(2):226–233.

7. Martinsson A, Li X, Zöller B, *et al.* Familial Aggregation of Aortic Valvular Stenosis: A Nationwide Study of Sibling Risk. *Circ Cardiovasc Genet*. 2017; 10(6):e001742.

8. Lindgren MP, Ji J, Smith JG, Sundquist J, Sundquist K, Zöller B. Mortality risks associated with sibling heart failure [published online ahead of print, 2019 Oct 12]. *Int J Cardiol.* 2019; S0167-5273(19)30895-2.

9. Berntsson J, Li X, Zöller B, *et al.* Risk of Stroke in Patients With Atrial Fibrillation Is Associated With Stroke in Siblings: A Nationwide Study. *J Am Heart Assoc.* 2020; 9(3):e014132.

10. Baranowska-Rataj A, Barclay K, Kolk M. The effect of number of siblings on adult mortality: Evidence from Swedish registers for cohorts born between 1938 and 1972. *Popul Stud (Camb).* 2017; 71(1):43–63.

11. Barclay K, Kolk M. Birth order and mortality: a population-based cohort study. Demography. 2015; 52(2):613–639.

12. Ayyavoo A, Derraik JG, Hofman PL, Cutfield WS. Is being first-born another risk factor for metabolic and cardiovascular diseases? *Future Cardiol.* 2013; 9(4):447–450.

13. Theodore RF, Broadbent J, Nagin D, et al. Childhood to Early-Midlife Systolic Blood Pressure Trajectories: Early-Life Predictors, Effect Modifiers, and Adult Cardiovascular Outcomes. *Hypertension.* 2015;66(6):1108–1115.

For peer review only

14. Albert BB, de Bock M, Derraik JG, et al. Among overweight middle-aged men, first-borns have lower insulin sensitivity than second-borns. *Sci Rep.* 2014; 4:3906.

15. Siervo M, Horta BL, Stephan BC, Victora CG, Wells JC. First-borns carry a higher metabolic risk in early adulthood: evidence from a prospective cohort study. *PLoS One*. 2010; 5(11):e13907.

16. Jelenkovic A, Silventoinen K, Tynelius P, Myrskylä M, Rasmussen F. Association of birth order with cardiovascular disease risk factors in young adulthood: a study of one million Swedish men. *PLoS One.* 2013; 8(5):e63361.

17. Barclay K, Myrskylä M. Birth order and physical fitness in early adulthood: evidence from Swedish military conscription data. *Soc Sci Med.* 2014; 123:141–148.

18. Julihn A, Soares FC, Hammarfjord U, Hjern A, Dahllöf G. Birth order is associated with caries development in young children: a register-based cohort study. *BMC Public Health.* 2020; 20(1):218.

19. Von Behren J, Spector LG, Mueller BA, *et al.* Birth order and risk of childhood cancer: a pooled analysis from five US States. *Int J Cancer.* 2011; 128(11):2709–2716.

20. Saarela J, Cederström A, Rostila M. Birth order and mortality in two ethno-linguistic groups: Register-based evidence from Finland. *Soc Sci Med.* 2016; 158:8–13.

21. Ludvigsson JF, Almqvist C, Bonamy AK, et al. Registers of the Swedish total population and their use in medical research. Eur J Epidemiol. 2016; 31(2):125-36.

22. Ludvigsson JF, Andersson E, Ekbom A, *et al.* External review and validation of the Swedish national inpatient register. *BMC Public Health.* 2011; 11:450.

23. Kalmijn M, van de Werfhorst HG. Sibship Size and Gendered Resource Dilution in Different Societal Contexts. PLoS One. 2016; 11(8):e0160953.

24. Dunford AR, Sangster JM. Maternal and paternal periconceptional nutrition as an indicator of offspring metabolic syndrome risk in later life through epigenetic imprinting: A systematic review. Diabetes Metab Syndr. 2017; 11 Suppl 2:S655-S662.

25. Young MF, Ramakrishnan U. Maternal Undernutrition before and during Pregnancy and Offspring Health and Development. Ann Nutr Metab. 2021 Feb 1:1-13.

26. Kristensen P, Bjerkedal T. Explaining the relation between birth order and intelligence. *Science* 2007; 316(5832):1717.

For peer review only - http://bmjopen.bmj.com/site/about/guidelines.xhtml

For peer review only

For peer review only - http://bmjopen.bmj.com/site/about/guidelines.xhtml

Table 1. Distribution of population, number of CVD, CHD, and mortality events

	Population		CVD events		CHD events		Mortality events	
	No.	(%)	No. (% of population)	%	No. (% of population)	%	No. (% of population)	%
**Men**	1358647		592863 (43.6)		131533 (9.7)		240371 (17.7)	
Number of sibling								
Non sibling	214700	15.8	105516 (49.1)	17.8	23671 (11.0)	18.0	50709 (23.6)	21.1
One sibling	443877	32.7	189839 (42.8)	32.0	39729 (9.0)	30.2	73140 (16.5)	30.4
Two siblings	338812	24.9	140361 (41.4)	23.7	30184 (8.9)	22.9	52790 (15.6)	22.0
Three siblings	183067	13.5	77378 (42.3)	13.1	17663 (9.6)	13.4	30266 (16.5)	12.6
Four or more siblings	178191	13.1	79769 (44.8)	13.5	20286 (11.4)	15.4	33466 (18.8)	13.9
Birth order								
First	684765	50.4	318341 (46.5)	53.7	70238 (10.3)	53.4	140857 (20.6)	58.6
Second	402879	29.7	166757 (41.4)	28.1	36654 (9.1)	27.9	62267 (15.5)	25.9
Third	164540	12.1	65853 (40.0)	11.1	14736 (9.0)	11.2	23081 (14.0)	9.6
Fourth	62765	4.6	24729 (39.4)	4.2	5737 (9.1)	4.4	8425 (13.4)	3.5
Fifth+	43698	3.2	17183 (39.3)	2.9	4168 (9.5)	3.2	5741 (13.1)	2.4
**Women**	1315037		486147 (37.0)		55933 (4.3)		160269 (12.2)	
Number of sibling								
Non sibling	210121	16.0	87261 (41.5)	17.9	10289 (4.9)	18.4	34521 (16.4)	21.5
One sibling	430315	32.7	154154 (35.8)	31.7	16280 (3.8)	29.1	49132 (11.4)	30.7
Two siblings	324379	24.7	113739 (35.1)	23.4	12500 (3.9)	22.3	34843 (10.7)	21.7
Three siblings	176631	13.4	63871 (36.2)	13.1	7512 (4.3)	13.4	19766 (11.2)	12.3
Four or more siblings	173591	13.2	67122 (38.7)	13.8	9352 (5.4)	16.7	22007 (12.7)	13.7
Birth order								
First	664459	50.5	262015 (39.4)	53.9	30342 (4.6)	54.2	94779 (14.3)	59.1
Second	388391	29.5	136263 (35.1)	28.0	15191 (3.9)	27.2	40984 (10.6)	25.6
Third	159311	12.1	53711 (33.7)	11.0	6231 (3.9)	11.1	15205 (9.5)	9.5
Fourth	60676	4.6	20264 (33.4)	4.2	2379 (3.9)	4.3	5558 (9.2)	3.5
Fifth+	42200	3.2	13894 (32.9)	2.9	1790 (4.2)	3.2	3743 (8.9)	2.3

For peer review only - http://bmjopen.bmj.com/site/about/guidelines.xhtml

Table 2. Hazard ratio (HR) and 95% confidence interval of CVD, CHD, and mortality in *men*

	CVD			CHD			Mortality		
	HR[a,b]	95% CI		HR[a]	95% CI		HR[a]	95% CI	
**Number of siblings (ref. Non sibling)**									
One sibling	0.98	0.97	0.99	0.99	0.97	1.01	0.93	0.92	0.94
Two siblings	0.97	0.97	0.98	1.01	0.99	1.03	0.91	0.90	0.92
Three siblings	0.98	0.97	0.99	1.04	1.02	1.07	0.93	0.92	0.94
Four or more siblings	1.00	0.99	1.01	1.10	1.07	1.12	0.96	0.94	0.97
**Birth order (ref. First birth)**									
Second	1.00	1.00	1.01	1.08	1.06	1.09	0.96	0.95	0.97
Third	1.02	1.02	1.03	1.13	1.11	1.15	0.98	0.96	0.99
Fourth	1.04	1.02	1.05	1.17	1.14	1.21	0.98	0.95	1.00
Fifth+	1.07	1.05	1.09	1.23	1.19	1.28	1.01	0.98	1.05

a: Full adjusted model: Adjusted for age at start, individual characteristics of family income, marital status, educational attainment, immigrant status, socioeconomic status, region of residence, comorbidities, number of siblings, and birth order
b: Multivariable competing risk survival analysis

For peer review only

Table 3. Hazard ratio (HR) and 95% confidence interval of CVD, CHD, and mortality in *women*

	CVD			CHD			Mortality		
	HR[a,b]	95% CI		HR[a]	95% CI		HR[a]	95% CI	
**Number of siblings (ref. Non sibling)**									
One sibling	0.98	0.98	0.99	0.99	0.97	1.02	0.94	0.93	0.95
Two siblings	0.99	0.98	1.00	1.03	1.00	1.06	0.92	0.91	0.94
Three siblings	1.00	0.99	1.01	1.07	1.04	1.11	0.93	0.91	0.95
Four or more siblings	1.01	1.00	1.03	1.17	1.13	1.21	0.95	0.93	0.96
**Birth order (ref. First birth)**									
Second	1.01	1.00	1.02	1.07	1.05	1.09	0.96	0.95	0.98
Third	1.02	1.01	1.03	1.14	1.11	1.18	0.98	0.96	1.00
Fourth	1.04	1.02	1.05	1.14	1.09	1.20	1.00	0.98	1.03
Fifth+	1.05	1.03	1.07	1.22	1.15	1.29	1.03	0.99	1.07

a: Full adjusted model: Adjusted for age at start, individual characteristics of family income, marital status, educational attainment, immigrant status, socioeconomic status, region of residence, comorbidities, number of siblings, and birth order
b: Multivariable competing risk survival analysis

For peer review only

For peer review only - http://bmjopen.bmj.com/site/about/guidelines.xhtml

Figure legends:

Figure 1. Hazard ratios of CHD by number of siblings and birth order in *men* (a,b) and *women* (c,d)

Figure 2. Hazard ratios of CVD by number of siblings and birth order in *men* (a,b) and *women* (c,d)

Figure 3. Hazard ratios of mortality by number of siblings and birth order in *men* (a,b) and *women* (c,d)

Supplemental Figure S1. Flow-chart of the study sample identified in the Swedish Multi-Generation Register (MGR).

For peer review only

For peer review only - http://bmjopen.bmj.com/site/about/guidelines.xhtml

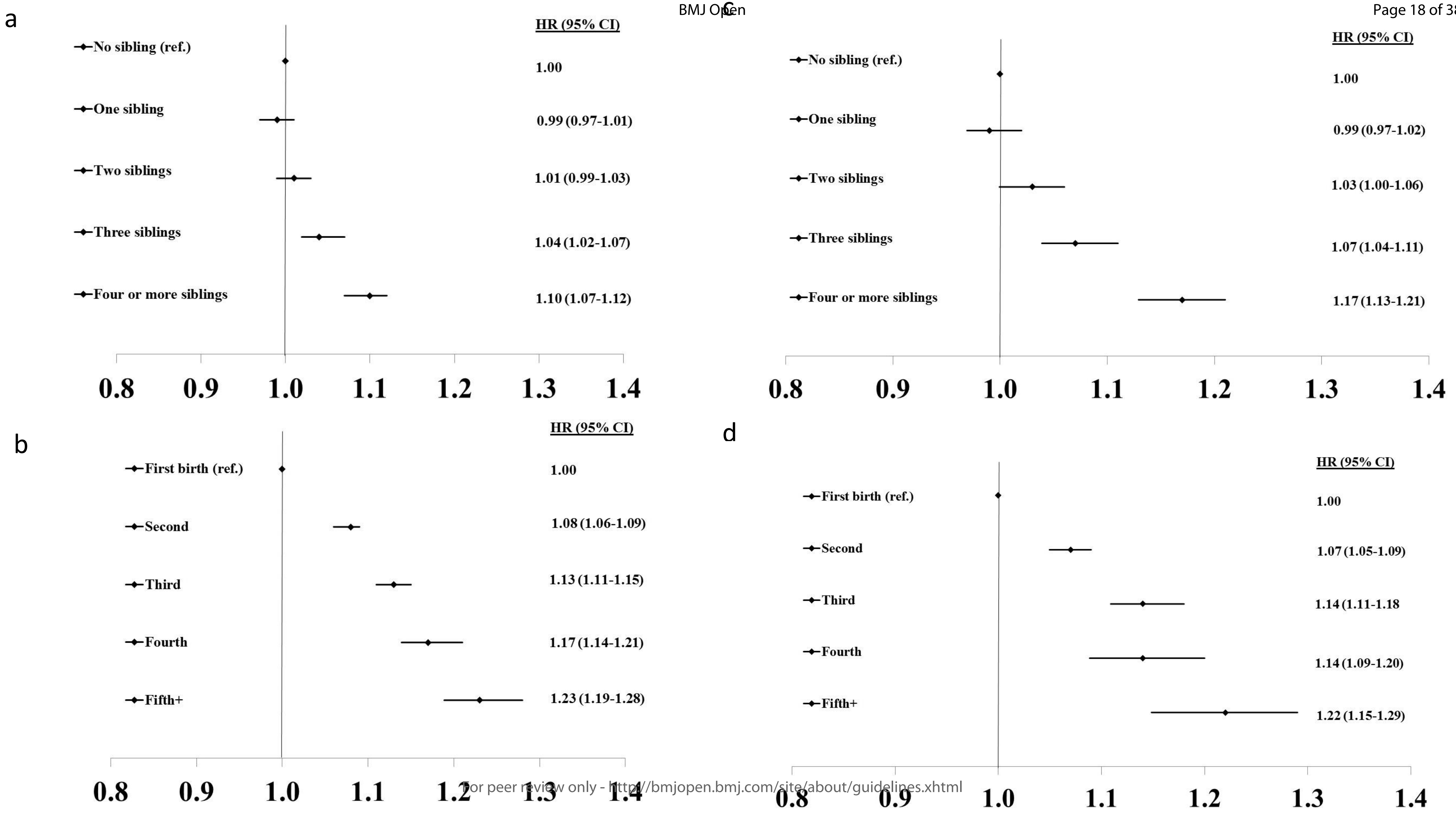
a
HR (95% CI)
No sibling (ref.)
1.00
One sibling
0.99 (0.97-1.01)
Two siblings
1.01 (0.99-1.03)
Three siblings
1.04 (1.02-1.07)
Four or more siblings
1.10 (1.07-1.12)
0.8 0.9 1.0 1.1 1.2 1.3 1.4
c
HR (95% CI)
No sibling (ref.)
1.00
One sibling
0.99 (0.97-1.02)
Two siblings
1.03 (1.00-1.06)
Three siblings
1.07 (1.04-1.11)
Four or more siblings
1.17 (1.13-1.21)
0.8 0.9 1.0 1.1 1.2 1.3 1.4
b
HR (95% CI)
First birth (ref.)
1.00
Second
1.08 (1.06-1.09)
Third
1.13 (1.11-1.15)
Fourth
1.17 (1.14-1.21)
Fifth+
1.23 (1.19-1.28)
0.8 0.9 1.0 1.1 1.2 1.3 1.4
d
HR (95% CI)
First birth (ref.)
1.00
Second
1.07 (1.05-1.09)
Third
1.14 (1.11-1.18)
Fourth
1.14 (1.09-1.20)
Fifth+
1.22 (1.15-1.29)
0.8 0.9 1.0 1.1 1.2 1.3 1.4

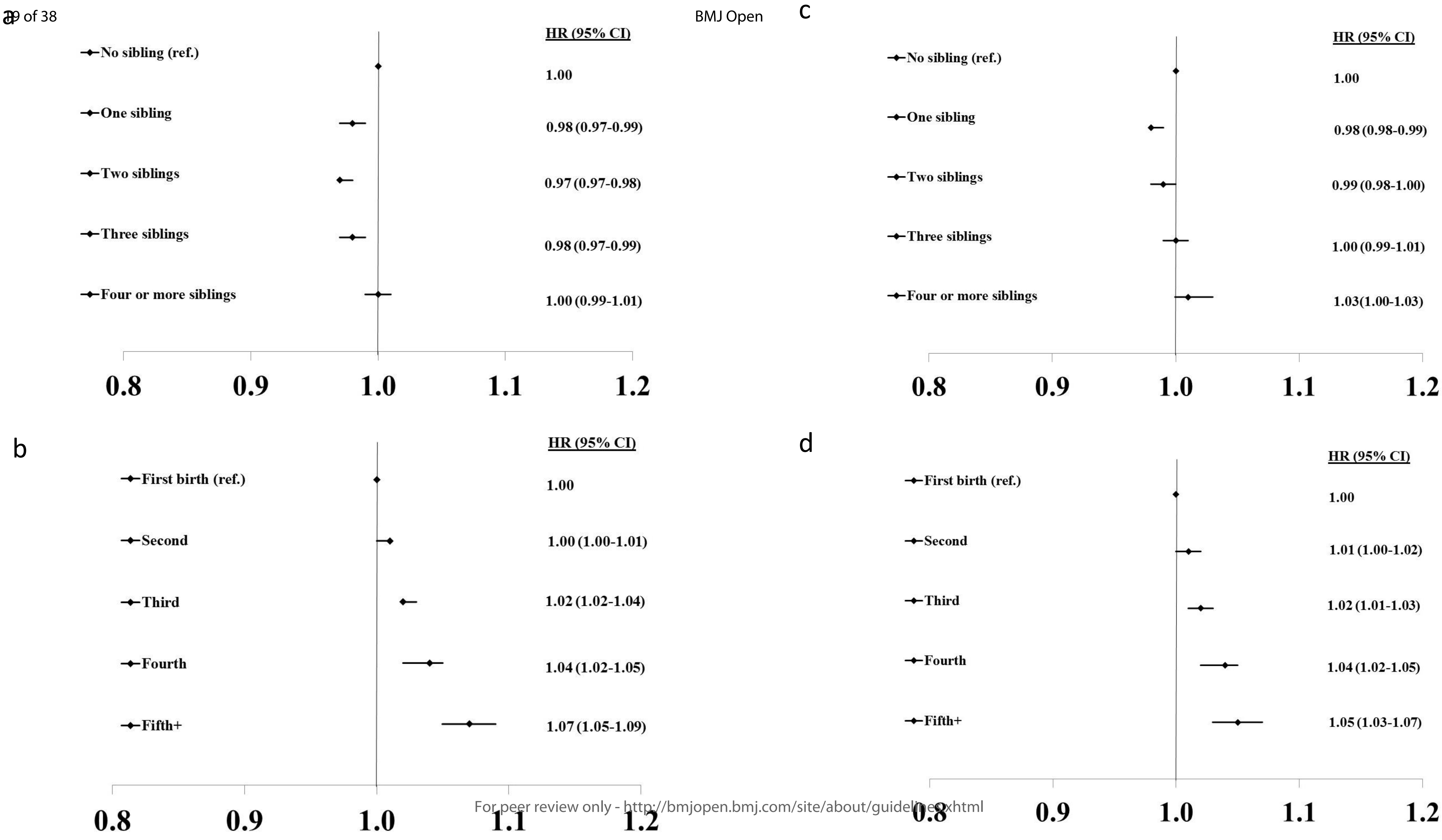
a
HR (95% CI)
No sibling (ref.)
1.00
One sibling
0.98 (0.97-0.99)
Two siblings
0.97 (0.97-0.98)
Three siblings
0.98 (0.97-0.99)
Four or more siblings
1.00 (0.99-1.01)
0.8
0.9
1.0
1.1
1.2
b
HR (95% CI)
First birth (ref.)
1.00
Second
1.00 (1.00-1.01)
Third
1.02 (1.02-1.04)
Fourth
1.04 (1.02-1.05)
Fifth+
1.07 (1.05-1.09)
0.8
0.9
1.0
1.1
1.2
c
HR (95% CI)
No sibling (ref.)
1.00
One sibling
0.98 (0.98-0.99)
Two siblings
0.99 (0.98-1.00)
Three siblings
1.00 (0.99-1.01)
Four or more siblings
1.03(1.00-1.03)
0.8
0.9
1.0
1.1
1.2
d
HR (95% CI)
First birth (ref.)
1.00
Second
1.01 (1.00-1.02)
Third
1.02 (1.01-1.03)
Fourth
1.04 (1.02-1.05)
Fifth+
1.05 (1.03-1.07)
0.8
0.9
1.0
1.1
1.2

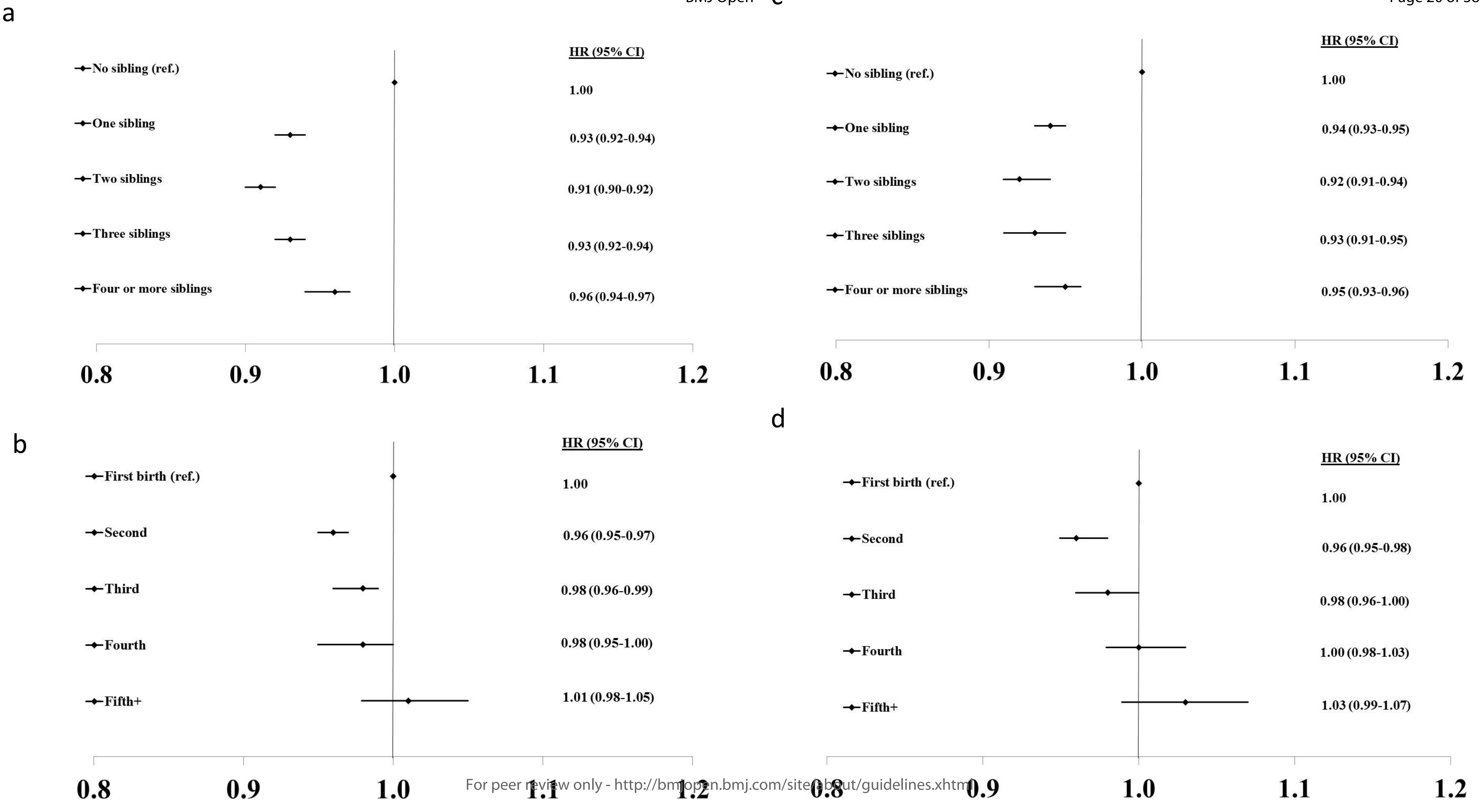
a
HR (95% CI)
No sibling (ref.)
1.00
One sibling
0.93 (0.92-0.94)
Two siblings
0.91 (0.90-0.92)
Three siblings
0.93 (0.92-0.94)
Four or more siblings
0.96 (0.94-0.97)
0.8
0.9
1.0
1.1
1.2
b
HR (95% CI)
First birth (ref.)
1.00
Second
0.96 (0.95-0.97)
Third
0.98 (0.96-0.99)
Fourth
0.98 (0.95-1.00)
Fifth+
1.01 (0.98-1.05)
0.8
0.9
1.0
1.1
1.2
c
HR (95% CI)
No sibling (ref.)
1.00
One sibling
0.94 (0.93-0.95)
Two siblings
0.92 (0.91-0.94)
Three siblings
0.93 (0.91-0.95)
Four or more siblings
0.95 (0.93-0.96)
0.8
0.9
1.0
1.1
1.2
d
HR (95% CI)
First birth (ref.)
1.00
Second
0.96 (0.95-0.98)
Third
0.98 (0.96-1.00)
Fourth
1.00 (0.98-1.03)
Fifth+
1.03 (0.99-1.07)
0.8
0.9
1.0
1.1
1.2

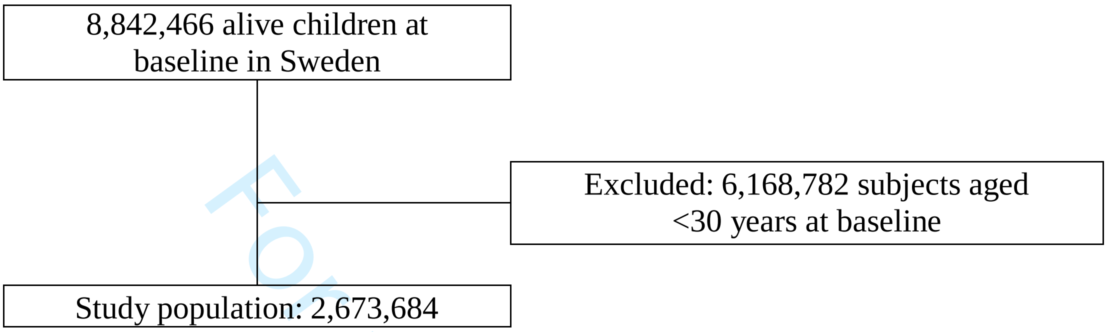

For peer review only

Supplementary Table 1a. Distribution of population, number of CVD, CHD, and mortality events in *men*.

	Population		CVD events		CHD events		Mortality events	
	No.	(%)	No.	%	No.	%	No.	%
Total population (%)	1358647		592863		131533		240371	
Age (years)								
30-39	531559	39.1	152615	25.7	25327	19.3	39094	16.3
40-49	554137	40.8	265251	44.7	59088	44.9	96549	40.2
50-58	272951	20.1	174997	29.5	47118	35.8	104728	43.6
Family income								
Low income	334083	24.6	123575	20.8	26649	20.3	47582	19.8
Middle–low income	320284	23.6	135148	22.8	29613	22.5	51243	21.3
Middle–high income	321653	23.7	152589	25.7	35041	26.6	66400	27.6
High income	382627	28.2	181551	30.6	40230	30.6	75146	31.3
Marital status								
Married/cohabiting	809314	59.6	370633	62.5	85212	64.8	124039	51.6
Never married, Widowed, or divorced	549333	40.4	222230	37.5	46321	35.2	116332	48.4
Educational attainment								
≤ 9 years	281207	20.7	146179	24.7	38890	29.6	81428	33.9
10–11 years	173208	12.7	67512	11.4	13955	10.6	26218	10.9
≥ 12 years	904232	66.6	379172	64.0	78688	59.8	132725	55.2
Immigrant status								
Sweden	1322658	97.4	580707	97.9	128732	97.9	235464	98.0
Other countries	35989	2.6	12156	2.1	2801	2.1	4907	2.0
Socioeconomic status								
Farmers/self-employed/others	308729	22.7	129408	21.8	29064	22.1	71960	29.9
Blue collar workers	494090	36.4	217215	36.6	50626	38.5	89989	37.4
White collar workers	351621	25.9	157170	26.5	34594	26.3	52582	21.9
Professionals	204207	15.0	89070	15.0	17249	13.1	25840	10.8
Urban/rural status								
Large cities	495681	36.5	207593	35.0	41153	31.3	85765	35.7
Southern Sweden	618686	45.5	274100	46.2	61581	46.8	108663	45.2
Northern Sweden	244280	18.0	111170	18.8	28799	21.9	45943	19.1
Hospitalization of alcoholism and related liver disease								
No	1275305	93.9	548796	92.6	124051	94.3	202724	84.3
Yes	83342	6.1	44067	7.4	7482	5.7	37647	15.7
Hospitalization of diabetes								
No	1226642	90.3	494000	83.3	104716	79.6	200226	83.3
Yes	132005	9.7	988863	16.7	26817	20.4	40145	16.7

For peer review only

Hospitalization of hypertension								
No	1059935	78.0	294151	49.6	76609	58.2	183958	76.5
Yes	298712	22.0	298712	50.4	54924	41.8	56413	23.5
Hospitalization of obesity								
No	1338892	98.5	577543	97.4	128595	97.8	236644	98.4
Yes	19755	1.5	15320	2.6	2938	2.2	3727	1.6
Hospitalization of chronic lower respiratory disease								
No	1288108	94.8	546393	92.2	120575	91.7	216522	90.1
Yes	70539	5.2	46470	7.8	10958	8.3	23849	9.9
Cancer								
No	1109571	81.7	452446	76.3	103202	78.5	139486	58.0
Yes	249076	18.3	140417	23.7	28331	21.5	100885	42.0
Number of sibling								
Non sibling	214700	15.8	105516	17.8	23671	18.0	50709	21.1
One sibling	443877	32.7	189839	32.0	39729	30.2	73140	30.4
Two siblings	338812	24.9	140361	23.7	30184	22.9	52790	22.0
Three siblings	183067	13.5	77378	13.1	17663	13.4	30266	12.6
Four or more siblings	178191	13.1	79769	13.5	20286	15.4	33466	13.9
Birth order								
First	684765	50.4	318341	53.7	70238	53.4	140857	58.6
Second	402879	29.7	166757	28.1	36654	27.9	62267	25.9
Third	164540	12.1	65853	11.1	14736	11.2	23081	9.6
Fourth	62765	4.6	24729	4.2	5737	4.4	8425	3.5
Fifth+	43698	3.2	17183	2.9	4168	3.2	5741	2.4

For peer review only

Supplementary Table 1b. Distribution of population, number of CVD, CHD, and mortality events in *women.*

	Population		CVD events		CHD events		Mortality events	
	No.	(%)	No.	%	No.	%	No.	%
Total population (%)	1315037		486147		55933		160269	
Age (years)								
30-39	507872	38.6	125326	25.8	9681	17.3	24571	15.3
40-49	537035	40.8	210113	43.2	23738	42.4	64055	40.0
50+	270130	20.5	150708	31.0	22514	40.3	71643	44.7
Family income								
Low income	343296	26.1	100542	20.7	10467	18.7	29723	18.5
Middle–low income	340837	25.9	121716	25.0	13432	24.0	37773	23.6
Middle–high income	347955	26.5	142924	29.4	17579	31.4	50537	31.5
High income	282949	21.5	120965	24.9	14455	25.8	42236	26.4
Marital status								
Married/cohabiting	887258	67.5	331886	68.3	38106	68.1	96533	60.2
Never married, Widowed, or divorced	427779	32.5	154261	31.7	17827	31.9	63736	39.8
Educational attainment								
≤ 9 years	232824	17.7	102037	21.0	15415	27.6	48455	30.2
10–11 years	165367	12.6	60209	12.4	6867	12.3	20489	12.8
≥ 12 years	916846	69.7	323901	66.6	33651	60.2	91325	57.0
Immigrant status								
Sweden	1281472	97.4	476075	97.9	54756	97.9	157515	98.3
Other countries	33565	2.6	10072	2.1	1177	2.1	2754	1.7
Socioeconomic status								
Farmers/self-employed/others	272189	20.7	96279	19.8	12450	22.3	45553	28.4
Blue collar workers	437324	33.3	174549	35.9	21821	39.0	55960	34.9
White collar workers	496782	37.8	178249	36.7	18324	32.8	49169	30.7
Professionals	108742	8.3	37070	7.6	3338	6.0	9587	6.0
Urban/rural status								
Large cities	495097	37.6	173872	35.8	17499	31.3	57794	36.1
Southern Sweden	591636	45.0	223375	45.9	25965	46.4	72872	45.5
Northern Sweden	228304	17.4	88900	18.3	12469	22.3	29603	18.5
Hospitalization of alcoholism and related liver disease								
No	1283561	97.6	471088	96.9	54416	97.3	149212	93.1
Yes	31476	2.4	15059	3.1	1517	2.7	11057	6.9
Hospitalization of diabetes								
No	1234361	93.9	429522	88.4	45537	81.4	139644	87.1

For peer review only

Yes	80676	6.1	56625	11.6	10396	18.6	20625	12.9
Hospitalization of hypertension								
No	1067928	81.2	239038	49.2	31364	56.1	124898	77.9
Yes	247109	18.8	247109	50.8	24569	43.9	35371	22.1
Hospitalization of obesity								
No	1286838	97.9	468228	96.3	54003	96.5	156771	97.8
Yes	28199	2.1	17919	3.7	1930	3.5	3498	2.2
Hospitalization of chronic lower respiratory disease								
No	1226846	93.3	433906	89.3	48084	86.0	137028	85.5
Yes	88191	6.7	52241	10.7	7849	14.0	23241	14.5
Cancer								
No	1051617	80.0	364226	74.9	43617	78.0	70300	43.9
Yes	263420	20.0	121921	25.1	12316	22.0	89969	56.1
Number of sibling								
Non sibling	210121	16.0	87261	17.9	10289	18.4	34521	21.5
One sibling	430315	32.7	154154	31.7	16280	29.1	49132	30.7
Two siblings	324379	24.7	113739	23.4	12500	22.3	34843	21.7
Three siblings	176631	13.4	63871	13.1	7512	13.4	19766	12.3
Four or more siblings	173591	13.2	67122	13.8	9352	16.7	22007	13.7
Birth order								
First	664459	50.5	262015	53.9	30342	54.2	94779	59.1
Second	388391	29.5	136263	28.0	15191	27.2	40984	25.6
Third	159311	12.1	53711	11.0	6231	11.1	15205	9.5
Fourth	60676	4.6	20264	4.2	2379	4.3	5558	3.5
Fifth+	42200	3.2	13894	2.9	1790	3.2	3743	2.3

For peer review only

Supplementary Table 2a. Hazard ratio (HR) and 95% confidence interval of CVD in *men*, using multivariable competing risk survival analysis

	Model 1			Model 2			Model 3		
	HR*	95% CI		HR*	95% CI		HR*	95% CI	
Number of siblings (ref. No sibling)									
One sibling	0.97	0.97	0.98	0.98	0.97	0.98	0.98	0.97	0.99
Two siblings	0.97	0.96	0.98	0.97	0.96	0.97	0.97	0.97	0.98
Three siblings	0.99	0.98	1.00	0.98	0.97	0.99	0.98	0.97	0.99
Four or more children	1.04	1.03	1.05	1.00	0.99	1.01	1.00	0.99	1.01
Age (years)	1.07	1.07	1.07	1.08	1.07	1.08	1.06	1.06	1.06
Family income (ref. High)									
Low income				0.92	0.92	0.93	0.99	0.98	1.00
Middle–low income				1.02	1.01	1.03	1.05	1.04	1.06
Middle–high income				1.04	1.03	1.05	1.05	1.04	1.06
Educational level (ref. > 12 years)									
10–11 years				1.11	1.10	1.12	1.06	1.05	1.07
≥ 12 years				1.00	1.00	1.01	1.00	0.99	1.00
Socioeconomic status (ref. professionals)									
Farmers/self-employed/others				1.22	1.21	1.23	1.17	1.16	1.18
Blue collar workers				1.15	1.14	1.16	1.09	1.08	1.10
White collar workers				1.08	1.07	1.08	1.04	1.04	1.05
Region of residence (ref. Large cities)									
Southern Sweden				1.00	1.00	1.01	1.03	1.02	1.03
Northern Sweden				1.04	1.03	1.05	1.05	1.04	1.06
Immigrant status (ref. Born in Sweden)				0.95	0.93	0.97	0.96	0.95	0.98
Marital status (ref. Not married)				1.18	1.17	1.19	1.17	1.16	1.18
Birth order				1.01	1.01	1.01	1.01	1.01	1.02
Hospitalization of chronic lower respiratory disease (ref. Non)							1.26	1.25	1.28
Hospitalization of alcoholisms and related liver disease (ref. Non)							1.74	1.73	1.76
Hospitalization of diabetes (ref. Non)							1.39	1.38	1.40
Hospitalization of obesity (ref. Non)							1.37	1.35	1.40
Hospitalization of hypertension (ref. Non)							3.72	3.70	3.74
Cancer (ref. Non)							1.36	1.35	1.36

Model 1. Adjusted for age at start; Model 2. Adjusted for age at start and individual characteristics; Model 3. Model 2 + comorbidities.
*: Multivariable competing risk survival analysis

For peer review only

Supplementary Table 2b. Hazard ratio (HR) and 95% confidence interval of CVD in *men*, using multivariable competing risk survival analysis

	Model 1			Model 2			Model 3		
	HR*	95% CI		HR*	95% CI		HR*	95% CI	
Birth order (ref. First birth)									
Second	0.99	0.99	1.00	0.99	0.98	1.00	1.00	1.00	1.01
Third	1.02	1.01	1.03	1.01	1.00	1.02	1.02	1.02	1.03
Fourth	1.05	1.03	1.06	1.03	1.01	1.04	1.04	1.02	1.05
Fifth+	1.10	1.08	1.12	1.07	1.05	1.08	1.07	1.05	1.09
Age (years)	1.07	1.07	1.07	1.08	1.07	1.08	1.06	1.06	1.06
Family income (ref. High)									
Low income				0.92	0.92	0.93	0.99	0.98	1.00
Middle–low income				1.02	1.01	1.03	1.05	1.04	1.06
Middle–high income				1.04	1.03	1.05	1.05	1.04	1.06
Educational level (ref. > 12 years)									
10–11 years				1.11	1.10	1.12	1.06	1.05	1.07
≥ 12 years				1.00	1.00	1.01	1.00	0.99	1.00
Socioeconomic status (ref. professionals)									
Farmers/self-employed/others				1.22	1.21	1.23	1.17	1.16	1.18
Blue collar workers				1.15	1.14	1.16	1.09	1.08	1.10
White collar workers				1.08	1.07	1.08	1.04	1.04	1.05
Region of residence (ref. Large cities)									
Southern Sweden				1.00	1.00	1.01	1.03	1.02	1.03
Northern Sweden				1.04	1.03	1.05	1.05	1.04	1.06
Immigrant status (ref. Born in Sweden)				0.95	0.93	0.97	0.97	0.95	0.98
Marital status (ref. Not married)				1.18	1.17	1.19	1.17	1.16	1.18
Number of siblings				1.00	1.00	1.00	1.00	1.00	1.00
Hospitalization of chronic lower respiratory disease (ref. Non)							1.26	1.25	1.28
Hospitalization of alcoholisms and related liver disease (ref. Non)							1.74	1.73	1.76
Hospitalization of diabetes (ref. Non)							1.39	1.38	1.40
Hospitalization of obesity (ref. Non)							1.37	1.35	1.40
Hospitalization of hypertension (ref. Non)							3.72	3.70	3.74
Cancer (ref. Non)							1.36	1.35	1.36

Model 1. Adjusted for age at start; Model 2. Adjusted for age at start and individual characteristics; Model 3. Model 2 + comorbidities.
*: Multivariable competing risk survival analysis

For peer review only

Supplementary Table 2c. Hazard ratio (HR) and 95% confidence interval of CVD in *women*, using multivariable competing risk survival analysis

	Model 1			Model 2			Model 3		
	HR*	95% CI		HR*	95% CI		HR*	95% CI	
Number of siblings (ref. No sibling)									
One sibling	0.98	0.97	0.98	0.98	0.97	0.98	0.98	0.98	0.99
Two siblings	0.99	0.98	0.99	0.98	0.97	0.99	0.99	0.98	1.00
Three siblings	1.01	1.00	1.02	1.00	0.99	1.01	1.00	0.99	1.01
Four or more children	1.06	1.05	1.07	1.03	1.02	1.04	1.01	1.00	1.03
Age (years)	1.06	1.06	1.07	1.06	1.06	1.06	1.04	1.04	1.04
Family income (ref. High)									
Low income				0.87	0.86	0.87	0.95	0.94	0.96
Middle–low income				1.01	1.00	1.02	1.04	1.03	1.05
Middle–high income				1.02	1.01	1.03	1.03	1.02	1.03
Educational level (ref. > 12 years)									
10–11 years				1.13	1.12	1.14	1.08	1.06	1.09
≥ 12 years				1.04	1.03	1.05	1.03	1.02	1.04
Socioeconomic status (ref. professionals)									
Farmers/self-employed/others				1.30	1.28	1.31	1.20	1.19	1.22
Blue collar workers				1.25	1.24	1.27	1.12	1.11	1.13
White collar workers				1.08	1.07	1.10	1.03	1.02	1.04
Region of residence (ref. Large cities)									
Southern Sweden				1.03	1.03	1.04	1.03	1.03	1.04
Northern Sweden				1.06	1.06	1.07	1.04	1.03	1.05
Immigrant status (ref. Born in Sweden)				0.97	0.96	0.99	0.98	0.96	1.00
Marital status (ref. Not married)				1.13	1.12	1.14	1.10	1.09	1.11
Birth order				1.00	1.00	1.01	1.01	1.01	1.01
Hospitalization of chronic lower respiratory disease (ref. Non)							1.38	1.36	1.39
Hospitalization of alcoholisms and related liver disease (ref. Non)							1.67	1.65	1.69
Hospitalization of diabetes (ref. Non)							1.38	1.37	1.39
Hospitalization of obesity (ref. Non)							1.32	1.30	1.34
Hospitalization of hypertension (ref. Non)							4.86	4.83	4.89
Cancer (ref. Non)							1.62	1.61	1.63

Model 1. Adjusted for age at start; Model 2. Adjusted for age at start and individual characteristics; Model 3. Model 2 + comorbidities.
*: Multivariable competing risk survival analysis

For peer review only

Supplementary Table 2d. Hazard ratio (HR) and 95% confidence interval of CVD in *women*, using multivariable competing risk survival analysis

	Model 1			Model 2			Model 3		
	HR*	95% CI		HR*	95% CI		HR*	95% CI	
Birth order (ref. First birth)									
Second	1.00	0.99	1.01	0.99	0.98	1.00	1.01	1.00	1.02
Third	1.03	1.02	1.04	1.00	0.99	1.01	1.02	1.01	1.03
Fourth	1.06	1.04	1.07	1.01	1.00	1.03	1.04	1.02	1.05
Fifth+	1.09	1.07	1.10	1.01	0.99	1.03	1.05	1.03	1.07
Age (years)	1.07	1.07	1.07	1.06	1.06	1.06	1.04	1.04	1.04
Family income (ref. High)									
Low income				0.87	0.86	0.87	0.95	0.94	0.96
Middle–low income				1.01	1.00	1.02	1.04	1.03	1.05
Middle–high income				1.02	1.01	1.03	1.03	1.02	1.03
Educational level (ref. > 12 years)									
10–11 years				1.13	1.12	1.14	1.08	1.06	1.09
≥ 12 years				1.04	1.03	1.05	1.03	1.02	1.04
Socioeconomic status (ref. professionals)									
Farmers/self-employed/others				1.30	1.28	1.31	1.21	1.19	1.22
Blue collar workers				1.26	1.24	1.27	1.12	1.11	1.13
White collar workers				1.09	1.07	1.10	1.03	1.02	1.05
Region of residence (ref. Large cities)									
Southern Sweden				1.03	1.03	1.04	1.03	1.03	1.04
Northern Sweden				1.06	1.06	1.07	1.04	1.03	1.05
Immigrant status (ref. Born in Sweden)				0.97	0.96	0.99	0.98	0.96	1.00
Marital status (ref. Not married)				1.13	1.13	1.14	1.10	1.09	1.11
Number of siblings				1.01	1.01	1.01	1.00	1.00	1.01
Hospitalization of chronic lower respiratory disease (ref. Non)							1.38	1.36	1.39
Hospitalization of alcoholisms and related liver disease (ref. Non)							1.67	1.65	1.69
Hospitalization of diabetes (ref. Non)							1.38	1.37	1.39
Hospitalization of obesity (ref. Non)							1.32	1.30	1.34
Hospitalization of hypertension (ref. Non)							4.86	4.83	4.89
Cancer (ref. Non)							1.62	1.61	1.63

Model 1. Adjusted for age at start; Model 2. Adjusted for age at start and individual characteristics; Model 3. Model 2 + comorbidities.

*: Multivariable competing risk survival analysis

For peer review only

Supplementary Table 3a. Hazard ratio (HR) and 95% confidence interval of CHD in *men*

	Model 1			Model 2			Model 3		
	HR	95% CI		HR	95% CI		HR	95% CI	
Number of siblings (ref. No sibling)									
One sibling	1.00	0.98	1.02	0.99	0.97	1.01	0.99	0.97	1.01
Two siblings	1.04	1.03	1.06	1.00	0.98	1.02	1.01	0.99	1.03
Three siblings	1.12	1.10	1.14	1.04	1.02	1.06	1.04	1.02	1.07
Four or more children	1.27	1.25	1.30	1.10	1.08	1.12	1.10	1.07	1.12
Age (years)	1.08	1.08	1.08	1.08	1.08	1.08	1.07	1.07	1.07
Family income (ref. High)									
Low income				0.93	0.91	0.94	0.96	0.95	0.98
Middle–low income				1.00	0.98	1.01	1.02	1.00	1.03
Middle–high income				1.01	1.00	1.03	1.02	1.00	1.03
Educational level (ref. > 12 years)									
10–11 years				1.05	1.03	1.07	1.04	1.02	1.07
≥ 12 years				0.94	0.93	0.95	0.95	0.94	0.96
Socioeconomic status (ref. professionals)									
Farmers/self-employed/others				1.23	1.20	1.25	1.18	1.16	1.21
Blue collar workers				1.26	1.24	1.28	1.21	1.19	1.23
White collar workers				1.18	1.16	1.20	1.15	1.13	1.17
Region of residence (ref. Large cities)									
Southern Sweden				1.11	1.10	1.12	1.12	1.11	1.14
Northern Sweden				1.32	1.30	1.34	1.31	1.29	1.33
Immigrant status (ref. Born in Sweden)				1.09	1.05	1.13	1.10	1.06	1.14
Marital status (ref. Not married)				1.00	0.99	1.01	0.99	0.98	1.01
Birth order				1.05	1.04	1.05	1.05	1.05	1.06
Hospitalization of chronic lower respiratory disease (ref. Non)							1.29	1.26	1.31
Hospitalization of alcoholisms and related liver disease (ref. Non)							1.01	0.99	1.04
Hospitalization of diabetes (ref. Non)							1.75	1.73	1.78
Hospitalization of obesity (ref. Non)							1.13	1.09	1.17
Hospitalization of hypertension (ref. Non)							1.86	1.84	1.88
Cancer (ref. Non)							0.91	0.89	0.92

Model 1. Adjusted for age at start; Model 2. Adjusted for age at start and individual characteristics; Model 3. Model 2 + comorbidities.

For peer review only

Supplementary Table 3b. Hazard ratio (HR) and 95% confidence interval of CHD in *men*

	Model 1			Model 2			Model 3		
	HR	95% CI		HR	95% CI		HR	95% CI	
Birth order (ref. First birth)									
Second	1.09	1.08	1.10	1.06	1.05	1.07	1.08	1.06	1.09
Third	1.19	1.17	1.21	1.11	1.09	1.13	1.13	1.11	1.15
Fourth	1.28	1.25	1.32	1.15	1.12	1.19	1.17	1.14	1.21
Fifth+	1.44	1.39	1.48	1.22	1.17	1.26	1.23	1.19	1.28
Age (years)	1.08	1.08	1.09	1.08	1.08	1.08	1.07	1.07	1.07
Family income (ref. High)									
Low income				0.93	0.91	0.94	0.96	0.95	0.98
Middle–low income				1.00	0.98	1.01	1.02	1.00	1.03
Middle–high income				1.01	1.00	1.02	1.02	1.00	1.03
Educational level (ref. > 12 years)									
10–11 years				1.05	1.03	1.08	1.05	1.03	1.07
≥ 12 years				0.94	0.93	0.95	0.95	0.94	0.97
Socioeconomic status (ref. professionals)									
Farmers/self-employed/others				1.23	1.20	1.25	1.18	1.16	1.21
Blue collar workers				1.26	1.24	1.28	1.21	1.19	1.23
White collar workers				1.18	1.16	1.20	1.15	1.13	1.17
Region of residence (ref. Large cities)									
Southern Sweden				1.11	1.10	1.13	1.12	1.11	1.14
Northern Sweden				1.32	1.30	1.34	1.31	1.29	1.33
Immigrant status (ref. Born in Sweden)				1.09	1.05	1.13	1.10	1.06	1.14
Marital status (ref. Not married)				1.00	0.99	1.01	0.99	0.98	1.01
Number of siblings				1.02	1.02	1.02	1.02	1.02	1.02
Hospitalization of chronic lower respiratory disease (ref. Non)							1.29	1.26	1.31
Hospitalization of alcoholisms and related liver disease (ref. Non)							1.01	0.99	1.04
Hospitalization of diabetes (ref. Non)							1.75	1.73	1.78
Hospitalization of obesity (ref. Non)							1.13	1.09	1.17
Hospitalization of hypertension (ref. Non)							1.86	1.84	1.88
Cancer (ref. Non)							0.91	0.89	0.92

Model 1. Adjusted for age at start; Model 2. Adjusted for age at start and individual characteristics; Model 3. Model 2 + comorbidities.

For peer review only

Supplementary Table 3c. Hazard ratio (HR) and 95% confidence interval of CHD in *women*

	Model 1			Model 2			Model 3		
	HR	95% CI		HR	95% CI		HR	95% CI	
Number of siblings (ref. No sibling)									
One sibling	0.99	0.97	1.02	0.98	0.96	1.01	0.99	0.97	1.02
Two siblings	1.07	1.04	1.10	1.03	1.00	1.05	1.03	1.00	1.06
Three siblings	1.17	1.13	1.20	1.08	1.04	1.11	1.07	1.04	1.11
Four or more children	1.40	1.36	1.44	1.20	1.16	1.24	1.17	1.13	1.21
Age (years)	1.09	1.09	1.09	1.09	1.08	1.09	1.07	1.07	1.07
Family income (ref. High)									
Low income				0.85	0.83	0.88	0.91	0.88	0.93
Middle–low income				1.00	0.98	1.03	1.00	0.98	1.03
Middle–high income				1.02	1.00	1.05	1.02	0.99	1.04
Educational level (ref. > 12 years)									
10–11 years				1.07	1.04	1.10	1.06	1.03	1.09
≥ 12 years				0.92	0.90	0.94	0.94	0.92	0.96
Socioeconomic status (ref. professionals)									
Farmers/self-employed/others				1.52	1.46	1.59	1.37	1.31	1.42
Blue collar workers				1.47	1.42	1.53	1.33	1.28	1.38
White collar workers				1.19	1.14	1.23	1.14	1.10	1.18
Region of residence (ref. Large cities)									
Southern Sweden				1.14	1.12	1.16	1.15	1.13	1.17
Northern Sweden				1.40	1.37	1.44	1.38	1.34	1.41
Immigrant status (ref. Born in Sweden)				1.13	1.07	1.20	1.13	1.07	1.20
Marital status (ref. Not married)				1.12	1.10	1.14	1.08	1.06	1.10
Birth order				1.04	1.03	1.05	1.05	1.04	1.06
Hospitalization of chronic lower respiratory disease (ref. Non)							1.69	1.65	1.73
Hospitalization of alcoholisms and related liver disease (ref. Non)							1.17	1.11	1.23
Hospitalization of diabetes (ref. Non)							2.20	2.15	2.25
Hospitalization of obesity (ref. Non)							1.11	1.06	1.17
Hospitalization of hypertension (ref. Non)							2.22	2.18	2.26
Cancer (ref. Non)							0.94	0.93	0.96

Model 1. Adjusted for age at start; Model 2. Adjusted for age at start and individual characteristics; Model 3. Model 2 + comorbidities.

For peer review only

Supplementary Table 3d. Hazard ratio (HR) and 95% confidence interval of CHD in *women*

	Model 1			Model 2			Model 3		
	HR	95% CI		HR	95% CI		HR	95% CI	
Birth order (ref. First birth)									
Second	1.10	1.07	1.12	1.05	1.02	1.07	1.07	1.05	1.09
Third	1.24	1.21	1.28	1.11	1.08	1.14	1.14	1.11	1.18
Fourth	1.33	1.27	1.38	1.11	1.06	1.16	1.14	1.09	1.20
Fifth+	1.56	1.48	1.63	1.17	1.11	1.24	1.22	1.15	1.29
Age (years)	1.09	1.09	1.09	1.09	1.08	1.09	1.07	1.07	1.07
Family income (ref. High)									
Low income				0.85	0.83	0.88	0.91	0.88	0.93
Middle–low income				1.00	0.98	1.03	1.01	0.98	1.03
Middle–high income				1.02	1.00	1.05	1.02	0.99	1.04
Educational level (ref. > 12 years)									
10–11 years				1.07	1.04	1.10	1.06	1.03	1.09
≥ 12 years				0.92	0.90	0.94	0.94	0.92	0.96
Socioeconomic status (ref. professionals)									
Farmers/self-employed/others				1.52	1.46	1.59	1.37	1.31	1.42
Blue collar workers				1.47	1.42	1.53	1.33	1.28	1.38
White collar workers				1.19	1.14	1.23	1.14	1.10	1.18
Region of residence (ref. Large cities)									
Southern Sweden				1.14	1.12	1.17	1.15	1.13	1.17
Northern Sweden				1.40	1.37	1.44	1.38	1.34	1.41
Immigrant status (ref. Born in Sweden)				1.13	1.07	1.20	1.13	1.07	1.20
Marital status (ref. Not married)				1.12	1.10	1.14	1.08	1.06	1.10
Number of siblings				1.04	1.03	1.04	1.03	1.03	1.04
Hospitalization of chronic lower respiratory disease (ref. Non)							1.69	1.65	1.73
Hospitalization of alcoholisms and related liver disease (ref. Non)							1.17	1.11	1.23
Hospitalization of diabetes (ref. Non)							2.20	2.15	2.25
Hospitalization of obesity (ref. Non)							1.11	1.06	1.17
Hospitalization of hypertension (ref. Non)							2.22	2.18	2.26
Cancer (ref. Non)							0.94	0.93	0.96

Model 1. Adjusted for age at start; Model 2. Adjusted for age at start and individual characteristics; Model 3. Model 2 + comorbidities.

For peer review only

Supplementary Table 4a. Hazard ratio (HR) and 95% confidence interval of mortality in *men*

	Model 1			Model 2			Model 3		
	HR	95% CI		HR	95% CI		HR	95% CI	
Number of siblings (ref. No sibling)									
One sibling	0.91	0.90	0.92	0.92	0.91	0.93	0.93	0.92	0.94
Two siblings	0.92	0.91	0.93	0.91	0.90	0.92	0.91	0.90	0.92
Three siblings	0.96	0.94	0.97	0.93	0.92	0.94	0.93	0.92	0.94
Four or more children	1.03	1.01	1.04	0.96	0.94	0.97	0.96	0.94	0.97
Age (years)	1.10	1.10	1.10	1.11	1.11	1.11	1.10	1.10	1.10
Family income (ref. High)									
Low income				1.05	1.04	1.07	1.08	1.07	1.10
Middle–low income				1.17	1.15	1.18	1.14	1.13	1.15
Middle–high income				1.18	1.17	1.19	1.15	1.14	1.16
Educational level (ref. > 12 years)									
10–11 years				1.12	1.10	1.13	1.08	1.06	1.09
≥ 12 years				0.91	0.90	0.91	0.89	0.88	0.90
Socioeconomic status (ref. professionals)									
Farmers/self-employed/others				1.73	1.71	1.76	1.63	1.60	1.65
Blue collar workers				1.38	1.36	1.40	1.34	1.32	1.36
White collar workers				1.15	1.13	1.17	1.14	1.12	1.16
Region of residence (ref. Large cities)									
Southern Sweden				0.94	0.93	0.95	0.99	0.98	1.00
Northern Sweden				0.98	0.97	0.99	1.08	1.07	1.09
Immigrant status (ref. Born in Sweden)				1.04	1.01	1.07	1.06	1.03	1.09
Marital status (ref. Not married)				1.88	1.87	1.90	1.71	1.70	1.73
Birth order				1.00	1.00	1.01	0.99	0.99	1.00
Hospitalization of chronic lower respiratory disease (ref. Non)							1.28	1.26	1.30
Hospitalization of alcoholisms and related liver disease (ref. Non)							3.02	2.98	3.05
Hospitalization of diabetes (ref. Non)							1.43	1.41	1.45
Hospitalization of obesity (ref. Non)							1.09	1.06	1.13
Hospitalization of hypertension (ref. Non)							0.66	0.65	0.67
Cancer (ref. Non)							2.62	2.60	2.65

Model 1. Adjusted for age at start; Model 2. Adjusted for age at start and individual characteristics; Model 3. Model 2 + comorbidities.

For peer review only

Supplementary Table 4b. Hazard ratio (HR) and 95% confidence interval of mortality in *men*

	Model 1			Model 2			Model 3		
	HR	95% CI		HR	95% CI		HR	95% CI	
Birth order (ref. First birth)									
Second	0.98	0.97	0.99	0.97	0.96	0.98	0.96	0.95	0.97
Third	1.01	0.99	1.02	0.99	0.98	1.01	0.98	0.96	0.99
Fourth	1.04	1.01	1.06	1.00	0.98	1.03	0.98	0.95	1.00
Fifth+	1.11	1.08	1.14	1.06	1.02	1.09	1.01	0.98	1.05
Age (years)	1.10	1.10	1.10	1.11	1.11	1.11	1.10	1.10	1.10
Family income (ref. High)									
Low income				1.06	1.04	1.07	1.08	1.07	1.10
Middle–low income				1.17	1.15	1.18	1.14	1.13	1.16
Middle–high income				1.18	1.17	1.19	1.15	1.14	1.16
Educational level (ref. > 12 years)									
10–11 years				1.12	1.10	1.13	1.07	1.06	1.09
≥ 12 years				0.90	0.90	0.91	0.89	0.88	0.90
Socioeconomic status (ref. professionals)									
Farmers/self-employed/others				1.73	1.71	1.76	1.63	1.60	1.66
Blue collar workers				1.38	1.36	1.40	1.34	1.32	1.36
White collar workers				1.15	1.13	1.17	1.14	1.13	1.16
Region of residence (ref. Large cities)									
Southern Sweden				0.94	0.93	0.95	0.99	0.98	1.00
Northern Sweden				0.98	0.97	0.99	1.08	1.07	1.09
Immigrant status (ref. Born in Sweden)				1.05	1.02	1.08	1.06	1.03	1.09
Marital status (ref. Not married)				1.88	1.87	1.90	1.71	1.70	1.73
Number of siblings				1.00	0.99	1.00	1.00	0.99	1.00
Hospitalization of chronic lower respiratory disease (ref. Non)							1.28	1.26	1.30
Hospitalization of alcoholisms and related liver disease (ref. Non)							3.02	2.98	3.05
Hospitalization of diabetes (ref. Non)							1.43	1.41	1.45
Hospitalization of obesity (ref. Non)							1.09	1.06	1.13
Hospitalization of hypertension (ref. Non)							0.66	0.65	0.67
Cancer (ref. Non)							2.62	2.60	2.64

Model 1. Adjusted for age at start; Model 2. Adjusted for age at start and individual characteristics; Model 3. Model 2 + comorbidities.

For peer review only

Supplementary Table 4c. Hazard ratio (HR) and 95% confidence interval of mortality in *women*

	Model 1			Model 2			Model 3		
	HR	95% CI		HR	95% CI		HR	95% CI	
Number of siblings (ref. No sibling)									
One sibling	0.92	0.91	0.94	0.93	0.92	0.95	0.94	0.93	0.95
Two siblings	0.92	0.91	0.94	0.91	0.90	0.93	0.92	0.91	0.94
Three siblings	0.95	0.93	0.96	0.92	0.90	0.93	0.93	0.91	0.95
Four or more children	1.01	0.99	1.03	0.94	0.92	0.95	0.95	0.93	0.96
Age (years)	1.10	1.10	1.10	1.10	1.10	1.10	1.09	1.08	1.09
Family income (ref. High)									
Low income				0.84	0.83	0.86	0.94	0.93	0.96
Middle–low income				1.03	1.01	1.05	1.06	1.04	1.07
Middle–high income				1.05	1.04	1.06	1.05	1.04	1.07
Educational level (ref. > 12 years)									
10–11 years				1.08	1.06	1.10	1.01	1.00	1.03
≥ 12 years				0.85	0.84	0.87	0.82	0.81	0.83
Socioeconomic status (ref. professionals)									
Farmers/self-employed/others				1.97	1.93	2.02	1.88	1.84	1.93
Blue collar workers				1.36	1.33	1.39	1.35	1.32	1.38
White collar workers				1.12	1.10	1.15	1.11	1.09	1.14
Region of residence (ref. Large cities)									
Southern Sweden				1.00	0.99	1.01	1.03	1.02	1.04
Northern Sweden				1.03	1.02	1.05	1.11	1.09	1.12
Immigrant status (ref. Born in Sweden)				0.89	0.86	0.92	0.95	0.92	0.99
Marital status (ref. Not married)				1.63	1.62	1.65	1.48	1.47	1.50
Birth order				1.01	1.00	1.01	1.00	0.99	1.01
Hospitalization of chronic lower respiratory disease (ref. Non)							1.61	1.58	1.63
Hospitalization of alcoholisms and related liver disease (ref. Non)							3.26	3.20	3.32
Hospitalization of diabetes (ref. Non)							1.58	1.56	1.61
Hospitalization of obesity (ref. Non)							0.96	0.93	0.99
Hospitalization of hypertension (ref. Non)							0.66	0.65	0.67
Cancer (ref. Non)							4.86	4.81	4.91

Model 1. Adjusted for age at start; Model 2. Adjusted for age at start and individual characteristics; Model 3. Model 2 + comorbidities.

For peer review only

Supplementary Table 4d. Hazard ratio (HR) and 95% confidence interval of mortality in *women*

	Model 1			Model 2			Model 3		
	HR	95% CI		HR	95% CI		HR	95% CI	
Birth order (ref. First birth)									
Second	0.98	0.96	0.99	0.97	0.96	0.98	0.96	0.95	0.98
Third	1.01	1.00	1.03	1.00	0.98	1.02	0.98	0.96	1.00
Fourth	1.05	1.02	1.08	1.02	0.99	1.05	1.00	0.98	1.03
Fifth+	1.11	1.08	1.15	1.08	1.04	1.12	1.03	0.99	1.07
Age (years)	1.10	1.10	1.10	1.10	1.10	1.10	1.09	1.08	1.09
Family income (ref. High)									
Low income				0.84	0.83	0.86	0.94	0.93	0.96
Middle–low income				1.03	1.02	1.05	1.06	1.04	1.07
Middle–high income				1.05	1.04	1.07	1.05	1.04	1.07
Educational level (ref. > 12 years)									
10–11 years				1.08	1.06	1.10	1.01	0.99	1.03
≥ 12 years				0.85	0.84	0.86	0.82	0.81	0.83
Socioeconomic status (ref. professionals)									
Farmers/self-employed/others				1.98	1.93	2.02	1.88	1.84	1.93
Blue collar workers				1.36	1.33	1.39	1.35	1.32	1.38
White collar workers				1.12	1.10	1.15	1.11	1.09	1.14
Region of residence (ref. Large cities)									
Southern Sweden				1.00	0.99	1.01	1.03	1.02	1.04
Northern Sweden				1.03	1.02	1.05	1.11	1.09	1.12
Immigrant status (ref. Born in Sweden)				0.89	0.86	0.92	0.95	0.92	0.99
Marital status (ref. Not married)				1.63	1.62	1.65	1.48	1.47	1.50
Number of siblings				0.99	0.99	0.99	0.99	0.99	1.00
Hospitalization of chronic lower respiratory disease (ref. Non)							1.61	1.58	1.63
Hospitalization of alcoholisms and related liver disease (ref. Non)							3.26	3.20	3.33
Hospitalization of diabetes (ref. Non)							1.58	1.56	1.61
Hospitalization of obesity (ref. Non)							0.96	0.93	0.99
Hospitalization of hypertension (ref. Non)							0.66	0.65	0.67
Cancer (ref. Non)							4.86	4.81	4.91

Model 1. Adjusted for age at start; Model 2. Adjusted for age at start and individual characteristics; Model 3. Model 2 + comorbidities.

For peer review only

STROBE 2007 (v4) Statement—Checklist of items that should be included in reports of *cohort studies*

Section/Topic	Item #	Recommendation	Reported on page #
**Title and abstract**	1	(*a*) Indicate the study's design with a commonly used term in the title or the abstract	1
		(*b*) Provide in the abstract an informative and balanced summary of what was done and what was found	2
**Introduction**			
Background/rationale	2	Explain the scientific background and rationale for the investigation being reported	3
Objectives	3	State specific objectives, including any prespecified hypotheses	3
**Methods**			
Study design	4	Present key elements of study design early in the paper	3-5
Setting	5	Describe the setting, locations, and relevant dates, including periods of recruitment, exposure, follow-up, and data collection	3-5
Participants	6	(*a*) Give the eligibility criteria, and the sources and methods of selection of participants. Describe methods of follow-up	3-5, and Figure S1
		(*b*) For matched studies, give matching criteria and number of exposed and unexposed	NA
Variables	7	Clearly define all outcomes, exposures, predictors, potential confounders, and effect modifiers. Give diagnostic criteria, if applicable	4
Data sources/ measurement	8*	For each variable of interest, give sources of data and details of methods of assessment (measurement). Describe comparability of assessment methods if there is more than one group	*4-5*
Bias	9	Describe any efforts to address potential sources of bias	4-5
Study size	10	Explain how the study size was arrived at	3
Quantitative variables	11	Explain how quantitative variables were handled in the analyses. If applicable, describe which groupings were chosen and why	4
Statistical methods	12	(*a*) Describe all statistical methods, including those used to control for confounding	5
		(*b*) Describe any methods used to examine subgroups and interactions	5
		(*c*) Explain how missing data were addressed	5
		(*d*) If applicable, explain how loss to follow-up was addressed	5
		(*e*) Describe any sensitivity analyses	5
**Results**			
Participants	13*	(a) Report numbers of individuals at each stage of study—eg numbers potentially eligible, examined for eligibility, confirmed eligible, included in the study, completing follow-up, and analysed	5

For peer review only

		(b) Give reasons for non-participation at each stage	NA
		(c) Consider use of a flow diagram	
Descriptive data	14*	(a) Give characteristics of study participants (eg demographic, clinical, social) and information on exposures and potential confounders	5, Table 1, and Supplementary Table 1a and 1b
		(b) Indicate number of participants with missing data for each variable of interest	NA
		(c) Summarise follow-up time (eg, average and total amount)	5
Outcome data	15*	Report numbers of outcome events or summary measures over time	5, Table 1, and Supplementary Table 1a, and 1b
Main results	16	(*a*) Give unadjusted estimates and, if applicable, confounder-adjusted estimates and their precision (eg, 95% confidence interval). Make clear which confounders were adjusted for and why they were included	5-6, Table 2 and 3, and Supplementary Tables 2a-2d, 3a-3d, and 4a-4d.
		(*b*) Report category boundaries when continuous variables were categorized	NA
		(*c*) If relevant, consider translating estimates of relative risk into absolute risk for a meaningful time period	NA
Other analyses	17	Report other analyses done—eg analyses of subgroups and interactions, and sensitivity analyses	5-6
**Discussion**			
Key results	18	Summarise key results with reference to study objectives	3, 7
**Limitations**			
Interpretation	20	Give a cautious overall interpretation of results considering objectives, limitations, multiplicity of analyses, results from similar studies, and other relevant evidence	3, 7, 8
Generalisability	21	Discuss the generalisability (external validity) of the study results	9
**Other information**			
Funding	22	Give the source of funding and the role of the funders for the present study and, if applicable, for the original study on which the present article is based	9

*Give information separately for cases and controls in case-control studies and, if applicable, for exposed and unexposed groups in cohort and cross-sectional studies.

Note: An Explanation and Elaboration article discusses each checklist item and gives methodological background and published examples of transparent reporting. The STROBE checklist is best used in conjunction with this article (freely available on the Web sites of PLoS Medicine at http://www.plosmedicine.org/, Annals of Internal Medicine at http://www.annals.org/, and Epidemiology at http://www.epidem.com/). Information on the STROBE Initiative is available at www.strobe-statement.org.

For peer review only

BMJ Open

Sibling rank and sibling number in relation to cardiovascular disease and mortality risk: a nationwide cohort study

Journal:	*BMJ Open*
Manuscript ID	bmjopen-2020-042881.R3
Article Type:	Original research
Date Submitted by the Author:	11-Apr-2021

Complete List of Authors:	Nilsson, Peter; Lunds Universitet, Clinical Sciences<br>Sundquist, Jan; Lund University, Center for Primary Health Care Research;<br>Sundquist, Kristina; Lund University, Center for Primary Health Care Research<br>Li, Xinjun; Lund University, Center for Primary Health Care Research

**Primary Subject Heading**:	Epidemiology
Secondary Subject Heading:	Cardiovascular medicine
Keywords:	EPIDEMIOLOGY, REPRODUCTIVE MEDICINE, SOCIAL MEDICINE

SCHOLARONE™
Manuscripts

I, the Submitting Author has the right to grant and does grant on behalf of all authors of the Work (as defined in the below author licence), an exclusive licence and/or a non-exclusive licence for contributions from authors who are: i) UK Crown employees; ii) where BMJ has agreed a CC-BY licence shall apply, and/or iii) in accordance with the terms applicable for US Federal Government officers or employees acting as part of their official duties; on a worldwide, perpetual, irrevocable, royalty-free basis to BMJ Publishing Group Ltd ("BMJ") its licensees and where the relevant Journal is co-owned by BMJ to the co-owners of the Journal, to publish the Work in this journal and any other BMJ products and to exploit all rights, as set out in our licence.

The Submitting Author accepts and understands that any supply made under these terms is made by BMJ to the Submitting Author unless you are acting as an employee on behalf of your employer or a postgraduate student of an affiliated institution which is paying any applicable article publishing charge ("APC") for Open Access articles. Where the Submitting Author wishes to make the Work available on an Open Access basis (and intends to pay the relevant APC), the terms of reuse of such Open Access shall be governed by a Creative Commons licence – details of these licences and which Creative Commons licence will apply to this Work are set out in our licence referred to above.

Other than as permitted in any relevant BMJ Author's Self Archiving Policies, I confirm this Work has not been accepted for publication elsewhere, is not being considered for publication elsewhere and does not duplicate material already published. I confirm all authors consent to publication of this Work and authorise the granting of this licence.

For peer review only

Original manuscript for the BMJ OPEN 2021 04 11/PN

Revised Version # 5.

Sibling rank and sibling number in relation to cardiovascular disease and mortality risk: a nationwide cohort study

Peter M Nilsson [1], Jan Sundquist [1,2], Kristina Sundquist [1,2] and Xinjun Li [1]

[1] Department of Clinical Sciences, Lund University, Skåne University Hospital, Malmö, Sweden

[2] Center for Primary Care Research, Lund University, Skåne University Hospital, Malmö, Sweden

For peer review only

Short title: Sibling rank, CVD and mortality risk

Number of words: 247 in the Abstract, 2160 in full text

Number of references: 26

Number of tables: 3 + 3 supplemental

Number of figures: 3 + 1 supplemental

Conflict of interest: None

Corresponding author:

Peter M Nilsson, MD, PhD

Lund University, Department of Clinical Sciences,

Skåne University Hospital,

Jan Waldenströms gata 15, 5th floor

S-205 02 Malmö, Sweden.

Email: Peter.Nilsson@med.lu.se

Phone: +46-40-33 24 15

For peer review only - http://bmjopen.bmj.com/site/about/guidelines.xhtml

Abstract

Background: The number and rank order of siblings could be of importance for risk of cardiovascular disease and mortality. Previous studies have used only fatal events for risk prediction. We therefore aimed to use also non-fatal coronary and cardiovascular events in fully adjusted models.

Methods: From the Multi-Generation Register in Sweden data were used from 1.36 million men and 1.32 million women (born 1932 to 1960), aged 30-58 years at baseline and with follow-up from 1990 to 2015. Mean age at follow-up was 67 years (range 55-83 years). Fatal and non-fatal events were retrieved from national registers.

Results:

Compared to men with no siblings, those with 1-2 siblings had a lower, and those with 4 or more siblings had a higher adjusted risk of cardiovascular events. Again, compared to men with no siblings, those with more than 1 sibling had a lower total mortality risk, and those with 3 or more siblings had an increased risk of coronary events.

Correspondingly, compared to women with no siblings those women with 3 siblings or more had an increased risk of cardiovascular events, and those with 2 siblings or more had an increased risk of coronary events. Women with 1 sibling or more were at lower total mortality risk, following full adjustment.

Conclusion: Being first-born is associated with a favourable effect on non-fatal cardiovascular and coronary events for both men and women. The underlying biological mechanisms for this should be studied in a socio-cultural context.

Key words: Cardiovascular, Coronary, Epidemiology, Mortality, Register, Risk, Sibling

For peer review only

Strengths and limitations of the study

- This national register linkage study across generations includes data on both fatal and, for the first time, non-fatal cardiovascular and coronary events, as well as total mortality in relation to sibling number and rank
- Adjustment has been made for confounders including markers of social background (educational level, occupation) of the individual, but not for parental socioeconomic status. Competing risk analyses have been applied
- Limitations of the study include the lack of data from primary health care visits and that the historical register data do not fully reflect the ethnical diversity of Sweden today

For peer review only

For peer review only - http://bmjopen.bmj.com/site/about/guidelines.xhtml

Introduction

A positive family history of disease is a well-established variable to be used in risk algorithms for cardiovascular disease (CVD), even if it may be hard to quantify based on subjective recall only [1,2]. An alternative is to use register-based data for a more objective appraisal of the family burden of CVD [3]. One special feature of family structure is the *number of siblings* and *sibling rank* that can also be mapped by use of national registers such as the Multiple-Generation Register (MGR) of Sweden [4]. So far this register has been mostly used to describe the risk of some selected CVD manifestations of individuals in relation to the risk of their siblings, for example for thromboembolic disease [5] or other diseases [6-9].

In a previous report based on the MGR it was shown that increased number of sibling reflecting family size was not associated with increased total and cause-specific mortality risk in ages 40-74 years, but no analyses were made for risk of non-fatal CVD or coronary disease [10]. However, another corresponding study based on MGR data could show that total and cause-specific mortality in ages 30-69 years increased with increasing birth order [11].

The influence of sibling rank has been less well studied in relation to non-fatal cardiovascular and coronary risk. Previous studies have indicated a worse cardiovascular risk factor burden in first-borns, for example increased body mass index (BMI) and systolic blood pressure, but lower insulin sensitivity, than later born siblings [12-16]. On the other hand, first-borns seem to have a better physical fitness at military conscript testing [17], less caries [18] and run a lower risk of leukemia in adolescence [19] as well as lower suicide risk in a Finnish population [20].

Against this background we *aimed* to analyse the influence of sibling number and rank on risk of non-fatal and fatal cardiovascular and coronary manifestations, as well as total mortality, in the MGR of Sweden, after extensive adjustment for background demographic factors and family social status.

For peer review only

Subjects and methods

Setting and participants

The dataset used in this study was constructed by linking several national Swedish registers. The Swedish government-owned Statistics Sweden provided the Multi-Generation Register (MGR), in which persons born in Sweden in or after 1932 (the present study population) are linked to their parents [4]. We included all births (multiple births, full and half siblings) in the analyses. Linkages were made to National Census data in order to ascertain individual-level socioeconomic status. The final link was made by adding data from the Swedish Cause of Death Register (1961-2015) and the Swedish Hospital Discharge Register, with recorded dates of hospitalization and hospital diagnoses

since 1964, but on a national level since 1987, and now until end of 2015. National Swedish registers are of high validity for medical research [21, 22]. For analysing risk of CVD, CHD and mortality in relation to number of siblings and birth order, we collected data from 1.36 million men and 1.32 million women (born 1932 to 1960), aged 30-58 years at baseline and with follow-up from 1990 to 2015. For the definition of study subjects based on the MGR, see supplemental **Figure S1**.

Patient and public involvement

No patient involved.

Follow-up of CVD events and total mortality

We used the following ICD-codes for fatal or non-fatal CVD (ICD-9, 390-459, ICD-10, I00-I99); and for CHD (ICD-9, 410-414, ICD-10, I20-I25). Non-fatal events were followed in the national Hospital Discharge Register, and fatal events and total mortality until 31st December 2015 in the national Mortality Register.

Definitions

Family income: Family income was calculated at start of follow-up (1990) as annual family income divided by the number of members in the family, as previously reported [23]. The income calculation was weighted, taking the ages of the family members into account. For example, children were given lower consumption weights than adults. The calculation was performed as follows: the sum of all family members' incomes was multiplied by the individual's consumption weight divided by the family members' total consumption weight. The final variable was calculated as empirical quartiles from the distribution and classified as low, middle-low, middle-high, and high.

Immigration status: born in Sweden or in other countries

Marital status: individuals were classified as married/cohabitating or never married, widowed, or divorced.

Socioeconomic status: was divided into four categories: the self-employed/farmers/all others, blue collar workers, white collar workers, or professionals, as previously reported [24]

Education: was based on educational level, which was classified into three categories: ≤ 9 years, 10–11 years, and ≥ 12 years

Geographic region: was divided into large cities (cities with a population of more than 200,000 inhabitants), Southern Sweden, and Northern Sweden [24]

Co-morbidity: was defined as the first hospitalization during the follow-up period of: chronic obstructive pulmonary disease, COPD (both hospitalization and mortality were included) (ICD-9 490–

For peer review only - http://bmjopen.bmj.com/site/about/guidelines.xhtml

496 and ICD-10 J40-J49), obesity (ICD-9 278A and ICD-10 E65-E68), alcoholism related liver disease (both hospitalizations and mortality were included) (ICD-9 291, 303, 571 and ICD-10 F10 and K70), hypertension (ICD-9 401-405 and ICD-10 I10–I15), and diabetes (both hospitalizations and mortality were included) (ICD-9 250 and ICD-10 E10-E14), and cancer (cancer were included both from cancer register and mortality, ICD-9 140-239, and ICD-10 C00-D48).

Statistical methods

Person-years at risk were calculated from the start of follow-up on 1 January 1990 until hospitalization or death from CVD, death from other causes, emigration, or the end of the follow-up, 31st December 2015. Age-adjusted incidence rates for first hospitalization and mortality were calculated for the entire follow-up period. We used the Cox's proportional hazard model to calculate the hazard ratio (HR) with 95 percent confidence intervals (CI) for total (fatal and non-fatal) CVD and CHD event risk, and for total mortality, for both men and women) in relation to number siblings and birth order. This was done after adjustment for age at start, individual characteristics (family income, marital status, immigrant background and educational level, region of residence, socioeconomic status), and finally for co-morbidities in order to adjust for competing mortality risk. Individuals without sibling was used as the reference. The proportionality assumptions were checked by plotting the incidence rates over time and by calculating Schoenfeld (partial) residuals and these assumptions were fulfilled. We used SAS version 9.4 (SAS Institute Inc. Cary, NC, USA) for all statistical analyses.

A further adjustment was made for total number of siblings in relation to birth order when the risk for different outcomes was calculated, using the category "first birth" as reference. A competing risk model used for mortality as a competing risk for incident CVD. A p-value < 0.05 was considered significant.

Results

With an average of 20 years (Q1-Q3 16-25 years) follow-up, in 1,358,647 men we used data on 592,863 CVD events, 131,533 coronary events, and 240,371 total deaths. For 1,315,037 women, the corresponding numbers were 486,147 CVD events, 55,933 coronary events and 160,269 deaths, respectively. The mean age of the study population at the end of the follow-up was 67 years (range 55-83 years). The number of siblings and birth order of men and women are depicted in **Table 1**.

Risk associated with number of siblings in men and women

Compared to men with no siblings, those with 1-2 siblings had a lower, and those with 4 or more siblings had a higher risk of cardiovascular events. Again, compared to men with no siblings, those

For peer review only - http://bmjopen.bmj.com/site/about/guidelines.xhtml

with more than 1 sibling had a lower total mortality risk, and those with 3 or more siblings had an increased risk of coronary events, following full adjustment, **Table 2.**

Correspondingly, compared to women with no siblings those women with 3 siblings or more had an increased risk of cardiovascular events, and those with 2 siblings or more had an increased risk of coronary events. Women with 1 sibling or more were at lower total mortality risk, following full adjustment, **Table 3.**

Risk associated with sibling rank in men and women

According to sibling rank, first-born men had a lower risk of both cardiovascular and coronary events than their later-born siblings, but higher total mortality than second and third-born siblings, following full adjustment, **Table 2**.

For first-born women the risks of cardiovascular and coronary events were also lower than in their later-born siblings. The mortality risk was higher than for second-born siblings, but equal to higher numbered siblings, following full adjustment, **Table 3**.

For hazard ratios of CHD, CVD and total mortality by number of siblings and birth order in men and women, respectively, see **Figures 1-3.**

Supplemental material

For detailed data on the distribution of the study population, number of CVD, coronary heart disease (CHD), and mortality events in men and women, see Supplemental **Table S1**. For detailed data on the risk associated with factors adjusted for, see Supplemental **Tables S2-S4,** for men and women, respectively.

Discussion

In this very large observational study based on a national Multi-Generation Register it was found that first-born men and women are at lower risk of both cardiovascular and coronary events than their later-born siblings, but had higher total mortality risk than second and third-born siblings (men). For women the mortality risk for first-born women was higher than for second-born siblings, following full adjustment for a number of background factors.

For total mortality in relation to sibling number our data are at odds with a previous study using the same register in Sweden, showing no increased mortality associated until 74 years with a higher number of siblings [10]. However, we used higher numbers, longer follow-up and more extensive adjustment.

For peer review only

For peer review only - http://bmjopen.bmj.com/site/about/guidelines.xhtml

For the influence of sibling rank, a previous study could show higher risk of total and cause-specific mortality with increasing sibling rank until 69 years [11]. This was similar in our study for risk of non-fatal cardiovascular and coronary events during longer follow-up and extensive adjustment.

These findings of lower cardiovascular risk in first-borns are at contrast to previous reports of a higher level of cardiovascular risk factors in such individuals followed until adolescence or young adulthood [12-16]. The burden of risk factors might have been compensated for by a better physical fitness, as noticed in first-born men coming for military conscript testing at the age of around 18 years [17]. In contrast to these observations, our extensive data indicate a lower cardiovascular risk in first-borns. Other unmeasured factors linked to being first-born, such as cognition or bodily development, could have contributed to our findings of a relative protection, even if we adjusted for a long list of potential confounders such as educational level, socio-economic status, marital status, and co-morbidities.

Besides filling a knowledge gap, this is of public health interest as different countries endorse different policies to support families and number of children. Our findings relate to family size and the biological as well as social roles related to sibling rank with its health implications.

More research is needed to understand the links between sibling number and rank with health outcomes. This could address, for example, the dilution of resources theory [25] of special relevance for disadvantaged girls; epigenetic factors influencing the metabolic syndrome in offspring [26]; and maternal health during pregnancy, including the effects of multiple births/child-rearing on maternal health and family resources, especially in deprived settings with large families [27].

Limitations and strengths of the study

The Swedish hospital discharge register contains no information about diagnostic procedures, which is a limitation. Moreover, specialist doctors in hospital care made the diagnosis. Another limitation is that we had no data on life-style related factors such as body mass index, smoking, and diet, because it would be unrealistic to gather such data for an entire national population. However, we did adjust for socioeconomic status, obesity, diabetes, COPD, and alcoholism and related liver disorders, which are associated with factors such as smoking and alcohol. Given a focus on family size, knowing that siblings who died young, and therefore not contributed to resource dilution for a proportion of the index person's childhood, would be of interest and importance. It would also shed light (potentially) on family circumstances and health. Regretfully, we currently lack data on parental SES to adjust for.

Strengths of the study include complete nationwide coverage from 1990 in a country with high standards of diagnosis, and with diagnoses often being made by specialists during extended examinations in clinics. Another important strength of our study is that it was based on nationwide registers and was thus free of selection and recall bias. The Swedish MGR and the Swedish Hospital

For peer review only - http://bmjopen.bmj.com/site/about/guidelines.xhtml

Discharge Register are validated data sources that have been proven to be reliable in the study of many diseases [4, 21,22]. Data in our dataset are almost 100% complete [4]. Generalisability (external validity) should hold at least for countries and populations similar to Sweden.

Future research should be directed to find biological or social mechanisms linking the status of being first-born to lower risk of cardiovascular disease, as indicated by our observational findings. A previous Norwegian study in military conscripts indicated that the role of being first-born is influenced by social factors, as a second-born son may achieve characteristics of a first-born brother who died young [28].

In conclusion, our data indicate a favourable effect on non-fatal cardiovascular and coronary events by being first-born, both for men and women.

a. Contributorship statement

PMN provided the original idea, and XL made the statistical analyses. PMN and XL drafted the first manuscript. All authors (PMN, XL, JS, and KS) contributed to the final manuscript. PMN and XL made the revisions.

b. Competing interests

None

c. Funding

This work was supported by the grants to Kristina Sundquist from The Swedish Research Council; ALF funding from Region Skåne and the Swedish Heart-Lung foundation.

d. Data sharing statement

Data will not be shared, but reasonable requests may be directed to Professor Kristina Sundquist, the PI of the registers.

e. Ethics statement

The study was approved by the Regional Ethics Review Board at the Lund University, Sweden (ID number 2012/795; approved 2013-02-06).

For peer review only - http://bmjopen.bmj.com/site/about/guidelines.xhtml

References

1. Banerjee A. A review of family history of cardiovascular disease: risk factor and research tool. *Int J Clin Pract*. 2012; 66:536-43.

2. Tada H, Melander O, Louie JZ, *et al.* Risk prediction by genetic risk scores for coronary heart disease is independent of self-reported family history. *Eur Heart J*. 2016;37(6):561–567.

3. Nilsson PM, Nilsson J-Å, and Berglund G. Family burden of cardiovascular mortality – risk implications for offspring in a national register linkage study based upon the Malmö Preventive Project. *J Internal Med* 2004; 255:229-35.

4. Ekbom A. The Swedish Multi-generation Register. *Methods Mol Biol*. 2011;675:215–220.

5. Zöller B, Ji J, Sundquist J, Sundquist K. Body Height and Incident Risk of Venous Thromboembolism: A Cosibling Design. *Circ Cardiovasc Genet.* 2017;10(5):e001651.

6. Kasiman K, Lundholm C, Sandin S, Malki N, Sparén P, Ingelsson E. Familial effects on ischemic stroke: the role of sibling kinship, sex, and age of onset. *Circ Cardiovasc Genet.* 2012;5(2):226–233.

7. Martinsson A, Li X, Zöller B, *et al.* Familial Aggregation of Aortic Valvular Stenosis: A Nationwide Study of Sibling Risk. *Circ Cardiovasc Genet*. 2017; 10(6):e001742.

8. Lindgren MP, Ji J, Smith JG, Sundquist J, Sundquist K, Zöller B. Mortality risks associated with sibling heart failure [published online ahead of print, 2019 Oct 12]. *Int J Cardiol.* 2019; S0167-5273(19)30895-2.

9. Berntsson J, Li X, Zöller B, *et al.* Risk of Stroke in Patients With Atrial Fibrillation Is Associated With Stroke in Siblings: A Nationwide Study. *J Am Heart Assoc.* 2020; 9(3):e014132.

10. Baranowska-Rataj A, Barclay K, Kolk M. The effect of number of siblings on adult mortality: Evidence from Swedish registers for cohorts born between 1938 and 1972. *Popul Stud (Camb).* 2017; 71(1):43–63.

11. Barclay K, Kolk M. Birth order and mortality: a population-based cohort study. Demography. 2015; 52(2):613–639.

12. Ayyavoo A, Derraik JG, Hofman PL, Cutfield WS. Is being first-born another risk factor for metabolic and cardiovascular diseases? *Future Cardiol.* 2013; 9(4):447–450.

13. Theodore RF, Broadbent J, Nagin D, et al. Childhood to Early-Midlife Systolic Blood Pressure Trajectories: Early-Life Predictors, Effect Modifiers, and Adult Cardiovascular Outcomes. *Hypertension.* 2015;66(6):1108–1115.

For peer review only

14. Albert BB, de Bock M, Derraik JG, et al. Among overweight middle-aged men, first-borns have lower insulin sensitivity than second-borns. *Sci Rep.* 2014; 4:3906.

15. Siervo M, Horta BL, Stephan BC, Victora CG, Wells JC. First-borns carry a higher metabolic risk in early adulthood: evidence from a prospective cohort study. *PLoS One*. 2010; 5(11):e13907.

16. Jelenkovic A, Silventoinen K, Tynelius P, Myrskylä M, Rasmussen F. Association of birth order with cardiovascular disease risk factors in young adulthood: a study of one million Swedish men. *PLoS One.* 2013; 8(5):e63361.

17. Barclay K, Myrskylä M. Birth order and physical fitness in early adulthood: evidence from Swedish military conscription data. *Soc Sci Med.* 2014; 123:141–148.

18. Julihn A, Soares FC, Hammarfjord U, Hjern A, Dahllöf G. Birth order is associated with caries development in young children: a register-based cohort study. *BMC Public Health.* 2020; 20(1):218.

19. Von Behren J, Spector LG, Mueller BA, *et al.* Birth order and risk of childhood cancer: a pooled analysis from five US States. *Int J Cancer.* 2011; 128(11):2709–2716.

20. Saarela J, Cederström A, Rostila M. Birth order and mortality in two ethno-linguistic groups: Register-based evidence from Finland. *Soc Sci Med.* 2016; 158:8–13.

21. Ludvigsson JF, Almqvist C, Bonamy AK, et al. Registers of the Swedish total population and their use in medical research. Eur J Epidemiol. 2016; 31(2):125-36.

22. Ludvigsson JF, Andersson E, Ekbom A, *et al.* External review and validation of the Swedish national inpatient register. *BMC Public Health.* 2011; 11:450.

23. Memarian E, Sundquist K, Calling S, Sundquist J, Li X. Socioeconomic factors, body mass index and bariatric surgery: a Swedish nationwide cohort study. *BMC Public Health*. 2019; 19(1):258.

24. Li X, Sundquist J, Zöller B, Sundquist K. Familial Risks of Glaucoma in the Population of Sweden. *J Glaucoma*. 2018; 27(9):802-806.

25. Kalmijn M, van de Werfhorst HG. Sibship Size and Gendered Resource Dilution in Different Societal Contexts. PLoS One. 2016; 11(8):e0160953.

26. Dunford AR, Sangster JM. Maternal and paternal periconceptional nutrition as an indicator of offspring metabolic syndrome risk in later life through epigenetic imprinting: A systematic review. Diabetes Metab Syndr. 2017; 11 Suppl 2:S655-S662.

27. Young MF, Ramakrishnan U. Maternal Undernutrition before and during Pregnancy and Offspring Health and Development. Ann Nutr Metab. 2021 Feb 1:1-13.

For peer review only - http://bmjopen.bmj.com/site/about/guidelines.xhtml

28. Kristensen P, Bjerkedal T. Explaining the relation between birth order and intelligence. *Science* 2007; 316(5832):1717.

For peer review only

For peer review only - http://bmjopen.bmj.com/site/about/guidelines.xhtml

Table 1. Distribution of population, number of CVD, CHD, and mortality events

	Population		CVD events		CHD events		Mortality events	
	No.	(%)	No. (% of population)	%	No. (% of population)	%	No. (% of population)	%
**Men**	1358647		592863 (43.6)		131533 (9.7)		240371 (17.7)	
Number of sibling								
Non sibling	214700	15.8	105516 (49.1)	17.8	23671 (11.0)	18.0	50709 (23.6)	21.1
One sibling	443877	32.7	189839 (42.8)	32.0	39729 (9.0)	30.2	73140 (16.5)	30.4
Two siblings	338812	24.9	140361 (41.4)	23.7	30184 (8.9)	22.9	52790 (15.6)	22.0
Three siblings	183067	13.5	77378 (42.3)	13.1	17663 (9.6)	13.4	30266 (16.5)	12.6
Four or more siblings	178191	13.1	79769 (44.8)	13.5	20286 (11.4)	15.4	33466 (18.8)	13.9
Birth order								
First	684765	50.4	318341 (46.5)	53.7	70238 (10.3)	53.4	140857 (20.6)	58.6
Second	402879	29.7	166757 (41.4)	28.1	36654 (9.1)	27.9	62267 (15.5)	25.9
Third	164540	12.1	65853 (40.0)	11.1	14736 (9.0)	11.2	23081 (14.0)	9.6
Fourth	62765	4.6	24729 (39.4)	4.2	5737 (9.1)	4.4	8425 (13.4)	3.5
Fifth+	43698	3.2	17183 (39.3)	2.9	4168 (9.5)	3.2	5741 (13.1)	2.4
**Women**	1315037		486147 (37.0)		55933 (4.3)		160269 (12.2)	
Number of sibling								
Non sibling	210121	16.0	87261 (41.5)	17.9	10289 (4.9)	18.4	34521 (16.4)	21.5
One sibling	430315	32.7	154154 (35.8)	31.7	16280 (3.8)	29.1	49132 (11.4)	30.7
Two siblings	324379	24.7	113739 (35.1)	23.4	12500 (3.9)	22.3	34843 (10.7)	21.7
Three siblings	176631	13.4	63871 (36.2)	13.1	7512 (4.3)	13.4	19766 (11.2)	12.3
Four or more siblings	173591	13.2	67122 (38.7)	13.8	9352 (5.4)	16.7	22007 (12.7)	13.7
Birth order								
First	664459	50.5	262015 (39.4)	53.9	30342 (4.6)	54.2	94779 (14.3)	59.1
Second	388391	29.5	136263 (35.1)	28.0	15191 (3.9)	27.2	40984 (10.6)	25.6
Third	159311	12.1	53711 (33.7)	11.0	6231 (3.9)	11.1	15205 (9.5)	9.5
Fourth	60676	4.6	20264 (33.4)	4.2	2379 (3.9)	4.3	5558 (9.2)	3.5
Fifth+	42200	3.2	13894 (32.9)	2.9	1790 (4.2)	3.2	3743 (8.9)	2.3

For peer review only - http://bmjopen.bmj.com/site/about/guidelines.xhtml

Table 2. Hazard ratio (HR) and 95% confidence interval of CVD, CHD, and mortality in *men*

	CVD			CHD			Mortality		
	HR[a,b]	95% CI		HR[a]	95% CI		HR[a]	95% CI	
**Number of siblings (ref. Non sibling)**									
One sibling	0.98	0.97	0.99	0.99	0.97	1.01	0.93	0.92	0.94
Two siblings	0.97	0.97	0.98	1.01	0.99	1.03	0.91	0.90	0.92
Three siblings	0.98	0.97	0.99	1.04	1.02	1.07	0.93	0.92	0.94
Four or more siblings	1.00	0.99	1.01	1.10	1.07	1.12	0.96	0.94	0.97
**Birth order (ref. First birth)**									
Second	1.00	1.00	1.01	1.08	1.06	1.09	0.96	0.95	0.97
Third	1.02	1.02	1.03	1.13	1.11	1.15	0.98	0.96	0.99
Fourth	1.04	1.02	1.05	1.17	1.14	1.21	0.98	0.95	1.00
Fifth+	1.07	1.05	1.09	1.23	1.19	1.28	1.01	0.98	1.05

a: Full adjusted model: Adjusted for age at start, individual characteristics of family income, marital status, educational attainment, immigrant status, socioeconomic status, region of residence, comorbidities, number of siblings, and birth order
b: Multivariable competing risk survival analysis

For peer review only

Table 3. Hazard ratio (HR) and 95% confidence interval of CVD, CHD, and mortality in *women*

	CVD			CHD			Mortality		
	HR[a,b]	95% CI		HR[a]	95% CI		HR[a]	95% CI	
**Number of siblings (ref. Non sibling)**									
One sibling	0.98	0.98	0.99	0.99	0.97	1.02	0.94	0.93	0.95
Two siblings	0.99	0.98	1.00	1.03	1.00	1.06	0.92	0.91	0.94
Three siblings	1.00	0.99	1.01	1.07	1.04	1.11	0.93	0.91	0.95
Four or more siblings	1.01	1.00	1.03	1.17	1.13	1.21	0.95	0.93	0.96
**Birth order (ref. First birth)**									
Second	1.01	1.00	1.02	1.07	1.05	1.09	0.96	0.95	0.98
Third	1.02	1.01	1.03	1.14	1.11	1.18	0.98	0.96	1.00
Fourth	1.04	1.02	1.05	1.14	1.09	1.20	1.00	0.98	1.03
Fifth+	1.05	1.03	1.07	1.22	1.15	1.29	1.03	0.99	1.07

a: Full adjusted model: Adjusted for age at start, individual characteristics of family income, marital status, educational attainment, immigrant status, socioeconomic status, region of residence, comorbidities, number of siblings, and birth order
b: Multivariable competing risk survival analysis

For peer review only

Figure legends:

Figure 1. Hazard ratios of CHD by number of siblings and birth order in *men* (a,b) and *women* (c,d)

Figure 2. Hazard ratios of CVD by number of siblings and birth order in *men* (a,b) and *women* (c,d)

Figure 3. Hazard ratios of mortality by number of siblings and birth order in *men* (a,b) and *women* (c,d)

Supplemental Figure S1. Flow-chart of the study sample identified in the Swedish Multi-Generation Register (MGR).

For peer review only

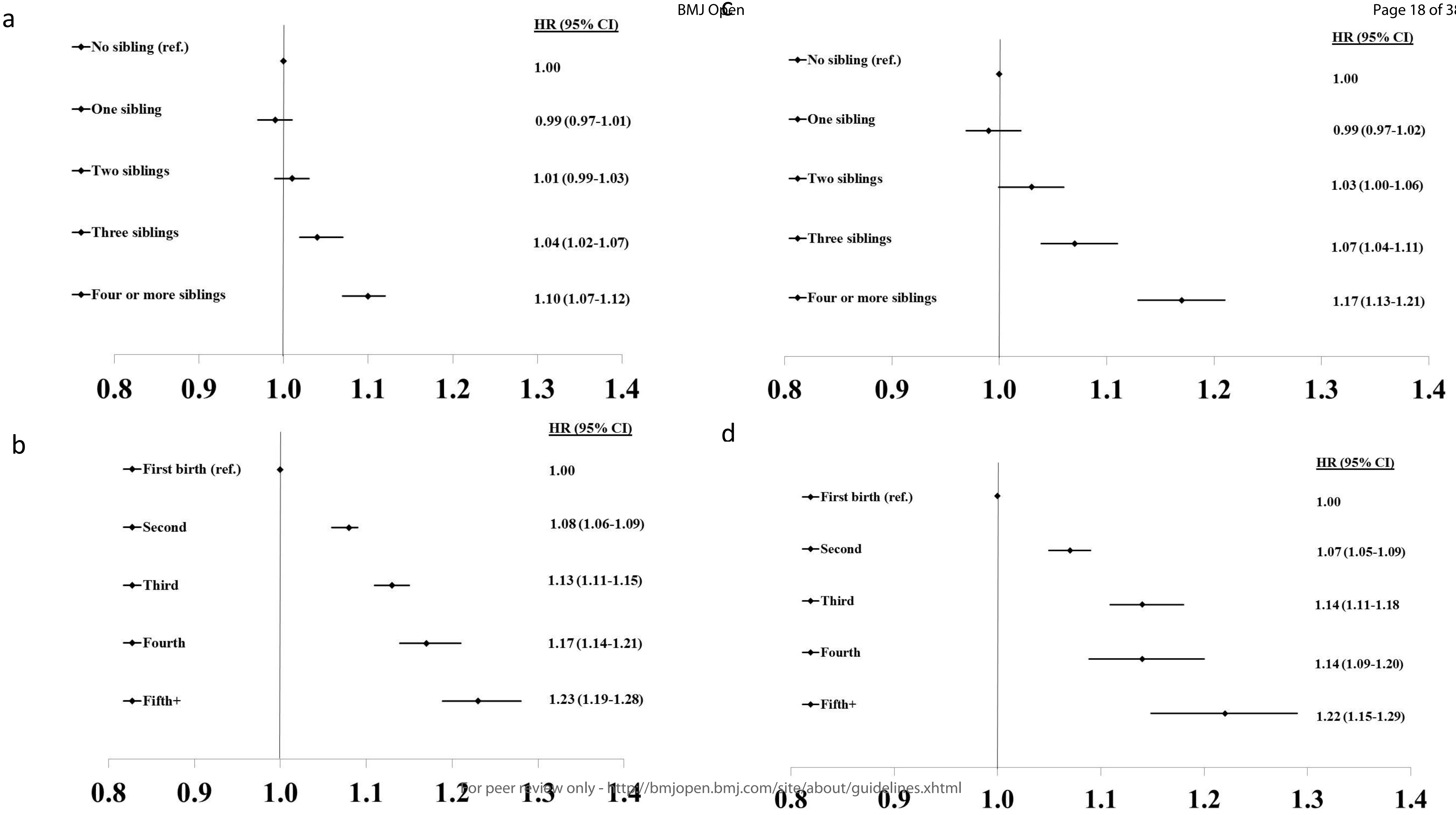
a
HR (95% CI)
No sibling (ref.)
1.00
One sibling
0.99 (0.97-1.01)
Two siblings
1.01 (0.99-1.03)
Three siblings
1.04 (1.02-1.07)
Four or more siblings
1.10 (1.07-1.12)
0.8 0.9 1.0 1.1 1.2 1.3 1.4
b
HR (95% CI)
First birth (ref.)
1.00
Second
1.08 (1.06-1.09)
Third
1.13 (1.11-1.15)
Fourth
1.17 (1.14-1.21)
Fifth+
1.23 (1.19-1.28)
0.8 0.9 1.0 1.1 1.2 1.3 1.4
c
HR (95% CI)
No sibling (ref.)
1.00
One sibling
0.99 (0.97-1.02)
Two siblings
1.03 (1.00-1.06)
Three siblings
1.07 (1.04-1.11)
Four or more siblings
1.17 (1.13-1.21)
0.8 0.9 1.0 1.1 1.2 1.3 1.4
d
HR (95% CI)
First birth (ref.)
1.00
Second
1.07 (1.05-1.09)
Third
1.14 (1.11-1.18)
Fourth
1.14 (1.09-1.20)
Fifth+
1.22 (1.15-1.29)
0.8 0.9 1.0 1.1 1.2 1.3 1.4

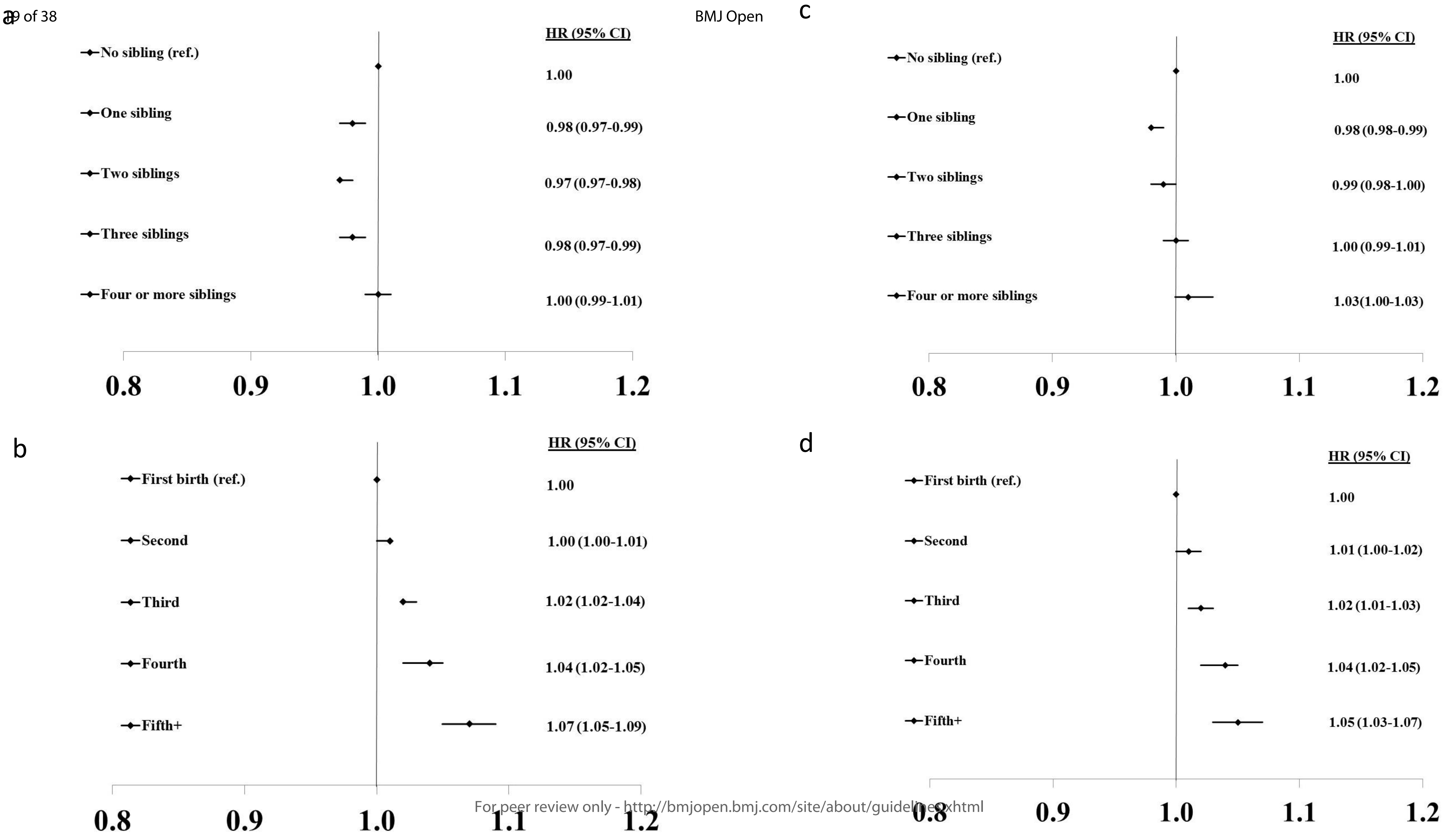
a
HR (95% CI)
No sibling (ref.)
1.00
One sibling
0.98 (0.97-0.99)
Two siblings
0.97 (0.97-0.98)
Three siblings
0.98 (0.97-0.99)
Four or more siblings
1.00 (0.99-1.01)
0.8
0.9
1.0
1.1
1.2
b
HR (95% CI)
First birth (ref.)
1.00
Second
1.00 (1.00-1.01)
Third
1.02 (1.02-1.04)
Fourth
1.04 (1.02-1.05)
Fifth+
1.07 (1.05-1.09)
0.8
0.9
1.0
1.1
1.2
c
HR (95% CI)
No sibling (ref.)
1.00
One sibling
0.98 (0.98-0.99)
Two siblings
0.99 (0.98-1.00)
Three siblings
1.00 (0.99-1.01)
Four or more siblings
1.03(1.00-1.03)
0.8
0.9
1.0
1.1
1.2
d
HR (95% CI)
First birth (ref.)
1.00
Second
1.01 (1.00-1.02)
Third
1.02 (1.01-1.03)
Fourth
1.04 (1.02-1.05)
Fifth+
1.05 (1.03-1.07)
0.8
0.9
1.0
1.1
1.2

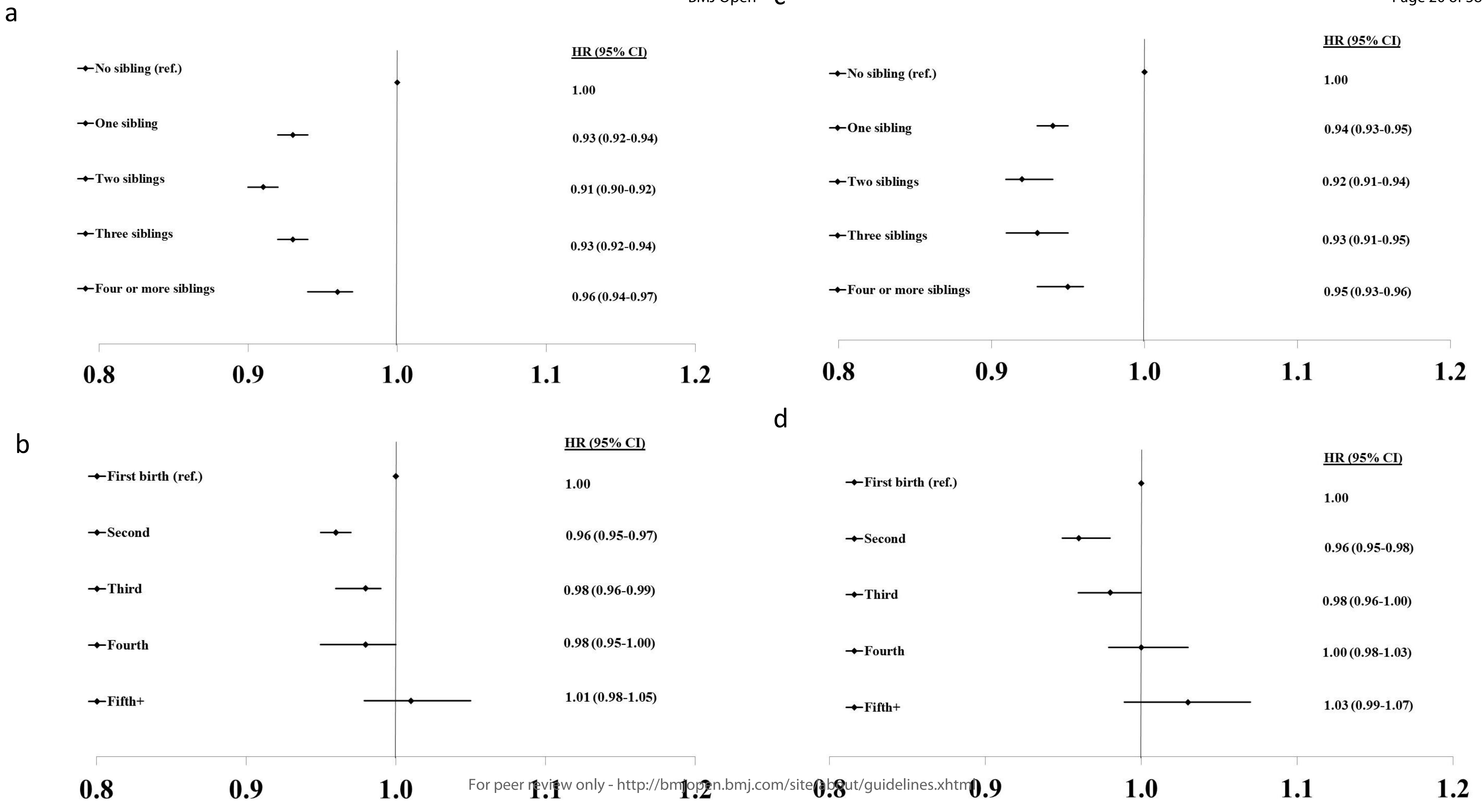
a
HR (95% CI)
No sibling (ref.)
1.00
One sibling
0.93 (0.92-0.94)
Two siblings
0.91 (0.90-0.92)
Three siblings
0.93 (0.92-0.94)
Four or more siblings
0.96 (0.94-0.97)
0.8
0.9
1.0
1.1
1.2
c
HR (95% CI)
No sibling (ref.)
1.00
One sibling
0.94 (0.93-0.95)
Two siblings
0.92 (0.91-0.94)
Three siblings
0.93 (0.91-0.95)
Four or more siblings
0.95 (0.93-0.96)
0.8
0.9
1.0
1.1
1.2
b
HR (95% CI)
First birth (ref.)
1.00
Second
0.96 (0.95-0.97)
Third
0.98 (0.96-0.99)
Fourth
0.98 (0.95-1.00)
Fifth+
1.01 (0.98-1.05)
0.8
0.9
1.0
1.1
1.2
d
HR (95% CI)
First birth (ref.)
1.00
Second
0.96 (0.95-0.98)
Third
0.98 (0.96-1.00)
Fourth
1.00 (0.98-1.03)
Fifth+
1.03 (0.99-1.07)
0.8
0.9
1.0
1.1
1.2

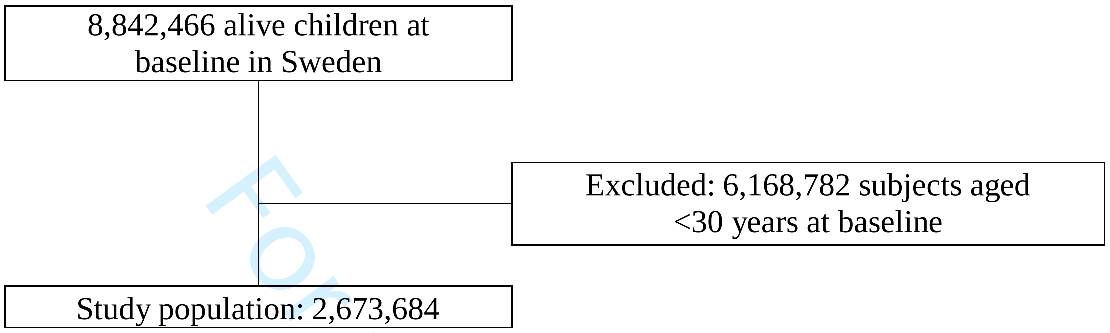

For peer review only

Supplementary Table 1a. Distribution of population, number of CVD, CHD, and mortality events in *men*.

	Population		CVD events		CHD events		Mortality events	
	No.	(%)	No.	%	No.	%	No.	%
Total population (%)	1358647		592863		131533		240371	
Age (years)								
30-39	531559	39.1	152615	25.7	25327	19.3	39094	16.3
40-49	554137	40.8	265251	44.7	59088	44.9	96549	40.2
50-58	272951	20.1	174997	29.5	47118	35.8	104728	43.6
Family income								
Low income	334083	24.6	123575	20.8	26649	20.3	47582	19.8
Middle–low income	320284	23.6	135148	22.8	29613	22.5	51243	21.3
Middle–high income	321653	23.7	152589	25.7	35041	26.6	66400	27.6
High income	382627	28.2	181551	30.6	40230	30.6	75146	31.3
Marital status								
Married/cohabiting	809314	59.6	370633	62.5	85212	64.8	124039	51.6
Never married, Widowed, or divorced	549333	40.4	222230	37.5	46321	35.2	116332	48.4
Educational attainment								
≤ 9 years	281207	20.7	146179	24.7	38890	29.6	81428	33.9
10–11 years	173208	12.7	67512	11.4	13955	10.6	26218	10.9
≥ 12 years	904232	66.6	379172	64.0	78688	59.8	132725	55.2
Immigrant status								
Sweden	1322658	97.4	580707	97.9	128732	97.9	235464	98.0
Other countries	35989	2.6	12156	2.1	2801	2.1	4907	2.0
Socioeconomic status								
Farmers/self-employed/others	308729	22.7	129408	21.8	29064	22.1	71960	29.9
Blue collar workers	494090	36.4	217215	36.6	50626	38.5	89989	37.4
White collar workers	351621	25.9	157170	26.5	34594	26.3	52582	21.9
Professionals	204207	15.0	89070	15.0	17249	13.1	25840	10.8
Urban/rural status								
Large cities	495681	36.5	207593	35.0	41153	31.3	85765	35.7
Southern Sweden	618686	45.5	274100	46.2	61581	46.8	108663	45.2
Northern Sweden	244280	18.0	111170	18.8	28799	21.9	45943	19.1
Hospitalization of alcoholism and related liver disease								
No	1275305	93.9	548796	92.6	124051	94.3	202724	84.3
Yes	83342	6.1	44067	7.4	7482	5.7	37647	15.7
Hospitalization of diabetes								
No	1226642	90.3	494000	83.3	104716	79.6	200226	83.3
Yes	132005	9.7	988863	16.7	26817	20.4	40145	16.7

For peer review only

Hospitalization of hypertension								
No	1059935	78.0	294151	49.6	76609	58.2	183958	76.5
Yes	298712	22.0	298712	50.4	54924	41.8	56413	23.5
Hospitalization of obesity								
No	1338892	98.5	577543	97.4	128595	97.8	236644	98.4
Yes	19755	1.5	15320	2.6	2938	2.2	3727	1.6
Hospitalization of chronic lower respiratory disease								
No	1288108	94.8	546393	92.2	120575	91.7	216522	90.1
Yes	70539	5.2	46470	7.8	10958	8.3	23849	9.9
Cancer								
No	1109571	81.7	452446	76.3	103202	78.5	139486	58.0
Yes	249076	18.3	140417	23.7	28331	21.5	100885	42.0
Number of sibling								
Non sibling	214700	15.8	105516	17.8	23671	18.0	50709	21.1
One sibling	443877	32.7	189839	32.0	39729	30.2	73140	30.4
Two siblings	338812	24.9	140361	23.7	30184	22.9	52790	22.0
Three siblings	183067	13.5	77378	13.1	17663	13.4	30266	12.6
Four or more siblings	178191	13.1	79769	13.5	20286	15.4	33466	13.9
Birth order								
First	684765	50.4	318341	53.7	70238	53.4	140857	58.6
Second	402879	29.7	166757	28.1	36654	27.9	62267	25.9
Third	164540	12.1	65853	11.1	14736	11.2	23081	9.6
Fourth	62765	4.6	24729	4.2	5737	4.4	8425	3.5
Fifth+	43698	3.2	17183	2.9	4168	3.2	5741	2.4

For peer review only

Supplementary Table 1b. Distribution of population, number of CVD, CHD, and mortality events in *women*.

	Population		CVD events		CHD events		Mortality events	
	No.	(%)	No.	%	No.	%	No.	%
Total population (%)	1315037		486147		55933		160269	
Age (years)								
30-39	507872	38.6	125326	25.8	9681	17.3	24571	15.3
40-49	537035	40.8	210113	43.2	23738	42.4	64055	40.0
50+	270130	20.5	150708	31.0	22514	40.3	71643	44.7
Family income								
Low income	343296	26.1	100542	20.7	10467	18.7	29723	18.5
Middle–low income	340837	25.9	121716	25.0	13432	24.0	37773	23.6
Middle–high income	347955	26.5	142924	29.4	17579	31.4	50537	31.5
High income	282949	21.5	120965	24.9	14455	25.8	42236	26.4
Marital status								
Married/cohabiting	887258	67.5	331886	68.3	38106	68.1	96533	60.2
Never married, Widowed, or divorced	427779	32.5	154261	31.7	17827	31.9	63736	39.8
Educational attainment								
≤ 9 years	232824	17.7	102037	21.0	15415	27.6	48455	30.2
10–11 years	165367	12.6	60209	12.4	6867	12.3	20489	12.8
≥ 12 years	916846	69.7	323901	66.6	33651	60.2	91325	57.0
Immigrant status								
Sweden	1281472	97.4	476075	97.9	54756	97.9	157515	98.3
Other countries	33565	2.6	10072	2.1	1177	2.1	2754	1.7
Socioeconomic status								
Farmers/self-employed/others	272189	20.7	96279	19.8	12450	22.3	45553	28.4
Blue collar workers	437324	33.3	174549	35.9	21821	39.0	55960	34.9
White collar workers	496782	37.8	178249	36.7	18324	32.8	49169	30.7
Professionals	108742	8.3	37070	7.6	3338	6.0	9587	6.0
Urban/rural status								
Large cities	495097	37.6	173872	35.8	17499	31.3	57794	36.1
Southern Sweden	591636	45.0	223375	45.9	25965	46.4	72872	45.5
Northern Sweden	228304	17.4	88900	18.3	12469	22.3	29603	18.5
Hospitalization of alcoholism and related liver disease								
No	1283561	97.6	471088	96.9	54416	97.3	149212	93.1
Yes	31476	2.4	15059	3.1	1517	2.7	11057	6.9
Hospitalization of diabetes								
No	1234361	93.9	429522	88.4	45537	81.4	139644	87.1

For peer review only

Yes	80676	6.1	56625	11.6	10396	18.6	20625	12.9
Hospitalization of hypertension								
No	1067928	81.2	239038	49.2	31364	56.1	124898	77.9
Yes	247109	18.8	247109	50.8	24569	43.9	35371	22.1
Hospitalization of obesity								
No	1286838	97.9	468228	96.3	54003	96.5	156771	97.8
Yes	28199	2.1	17919	3.7	1930	3.5	3498	2.2
Hospitalization of chronic lower respiratory disease								
No	1226846	93.3	433906	89.3	48084	86.0	137028	85.5
Yes	88191	6.7	52241	10.7	7849	14.0	23241	14.5
Cancer								
No	1051617	80.0	364226	74.9	43617	78.0	70300	43.9
Yes	263420	20.0	121921	25.1	12316	22.0	89969	56.1
Number of sibling								
Non sibling	210121	16.0	87261	17.9	10289	18.4	34521	21.5
One sibling	430315	32.7	154154	31.7	16280	29.1	49132	30.7
Two siblings	324379	24.7	113739	23.4	12500	22.3	34843	21.7
Three siblings	176631	13.4	63871	13.1	7512	13.4	19766	12.3
Four or more siblings	173591	13.2	67122	13.8	9352	16.7	22007	13.7
Birth order								
First	664459	50.5	262015	53.9	30342	54.2	94779	59.1
Second	388391	29.5	136263	28.0	15191	27.2	40984	25.6
Third	159311	12.1	53711	11.0	6231	11.1	15205	9.5
Fourth	60676	4.6	20264	4.2	2379	4.3	5558	3.5
Fifth+	42200	3.2	13894	2.9	1790	3.2	3743	2.3

For peer review only

Supplementary Table 2a. Hazard ratio (HR) and 95% confidence interval of CVD in *men*, using multivariable competing risk survival analysis

	Model 1			Model 2			Model 3		
	HR*	95% CI		HR*	95% CI		HR*	95% CI	
Number of siblings (ref. No sibling)									
One sibling	0.97	0.97	0.98	0.98	0.97	0.98	0.98	0.97	0.99
Two siblings	0.97	0.96	0.98	0.97	0.96	0.97	0.97	0.97	0.98
Three siblings	0.99	0.98	1.00	0.98	0.97	0.99	0.98	0.97	0.99
Four or more children	1.04	1.03	1.05	1.00	0.99	1.01	1.00	0.99	1.01
Age (years)	1.07	1.07	1.07	1.08	1.07	1.08	1.06	1.06	1.06
Family income (ref. High)									
Low income				0.92	0.92	0.93	0.99	0.98	1.00
Middle–low income				1.02	1.01	1.03	1.05	1.04	1.06
Middle–high income				1.04	1.03	1.05	1.05	1.04	1.06
Educational level (ref. > 12 years)									
10–11 years				1.11	1.10	1.12	1.06	1.05	1.07
≥ 12 years				1.00	1.00	1.01	1.00	0.99	1.00
Socioeconomic status (ref. professionals)									
Farmers/self-employed/others				1.22	1.21	1.23	1.17	1.16	1.18
Blue collar workers				1.15	1.14	1.16	1.09	1.08	1.10
White collar workers				1.08	1.07	1.08	1.04	1.04	1.05
Region of residence (ref. Large cities)									
Southern Sweden				1.00	1.00	1.01	1.03	1.02	1.03
Northern Sweden				1.04	1.03	1.05	1.05	1.04	1.06
Immigrant status (ref. Born in Sweden)				0.95	0.93	0.97	0.96	0.95	0.98
Marital status (ref. Not married)				1.18	1.17	1.19	1.17	1.16	1.18
Birth order				1.01	1.01	1.01	1.01	1.01	1.02
Hospitalization of chronic lower respiratory disease (ref. Non)							1.26	1.25	1.28
Hospitalization of alcoholisms and related liver disease (ref. Non)							1.74	1.73	1.76
Hospitalization of diabetes (ref. Non)							1.39	1.38	1.40
Hospitalization of obesity (ref. Non)							1.37	1.35	1.40
Hospitalization of hypertension (ref. Non)							3.72	3.70	3.74
Cancer (ref. Non)							1.36	1.35	1.36

Model 1. Adjusted for age at start; Model 2. Adjusted for age at start and individual characteristics; Model 3. Model 2 + comorbidities.
*: Multivariable competing risk survival analysis

For peer review only

Supplementary Table 2b. Hazard ratio (HR) and 95% confidence interval of CVD in *men*, using multivariable competing risk survival analysis

	Model 1			Model 2			Model 3		
	HR*	95% CI		HR*	95% CI		HR*	95% CI	
Birth order (ref. First birth)									
Second	0.99	0.99	1.00	0.99	0.98	1.00	1.00	1.00	1.01
Third	1.02	1.01	1.03	1.01	1.00	1.02	1.02	1.02	1.03
Fourth	1.05	1.03	1.06	1.03	1.01	1.04	1.04	1.02	1.05
Fifth+	1.10	1.08	1.12	1.07	1.05	1.08	1.07	1.05	1.09
Age (years)	1.07	1.07	1.07	1.08	1.07	1.08	1.06	1.06	1.06
Family income (ref. High)									
Low income				0.92	0.92	0.93	0.99	0.98	1.00
Middle–low income				1.02	1.01	1.03	1.05	1.04	1.06
Middle–high income				1.04	1.03	1.05	1.05	1.04	1.06
Educational level (ref. > 12 years)									
10–11 years				1.11	1.10	1.12	1.06	1.05	1.07
≥ 12 years				1.00	1.00	1.01	1.00	0.99	1.00
Socioeconomic status (ref. professionals)									
Farmers/self-employed/others				1.22	1.21	1.23	1.17	1.16	1.18
Blue collar workers				1.15	1.14	1.16	1.09	1.08	1.10
White collar workers				1.08	1.07	1.08	1.04	1.04	1.05
Region of residence (ref. Large cities)									
Southern Sweden				1.00	1.00	1.01	1.03	1.02	1.03
Northern Sweden				1.04	1.03	1.05	1.05	1.04	1.06
Immigrant status (ref. Born in Sweden)				0.95	0.93	0.97	0.97	0.95	0.98
Marital status (ref. Not married)				1.18	1.17	1.19	1.17	1.16	1.18
Number of siblings				1.00	1.00	1.00	1.00	1.00	1.00
Hospitalization of chronic lower respiratory disease (ref. Non)							1.26	1.25	1.28
Hospitalization of alcoholisms and related liver disease (ref. Non)							1.74	1.73	1.76
Hospitalization of diabetes (ref. Non)							1.39	1.38	1.40
Hospitalization of obesity (ref. Non)							1.37	1.35	1.40
Hospitalization of hypertension (ref. Non)							3.72	3.70	3.74
Cancer (ref. Non)							1.36	1.35	1.36

Model 1. Adjusted for age at start; Model 2. Adjusted for age at start and individual characteristics; Model 3. Model 2 + comorbidities.
*: Multivariable competing risk survival analysis

For peer review only

Supplementary Table 2c. Hazard ratio (HR) and 95% confidence interval of CVD in *women*, using multivariable competing risk survival analysis

	Model 1			Model 2			Model 3		
	HR*	95% CI		HR*	95% CI		HR*	95% CI	
Number of siblings (ref. No sibling)									
One sibling	0.98	0.97	0.98	0.98	0.97	0.98	0.98	0.98	0.99
Two siblings	0.99	0.98	0.99	0.98	0.97	0.99	0.99	0.98	1.00
Three siblings	1.01	1.00	1.02	1.00	0.99	1.01	1.00	0.99	1.01
Four or more children	1.06	1.05	1.07	1.03	1.02	1.04	1.01	1.00	1.03
Age (years)	1.06	1.06	1.07	1.06	1.06	1.06	1.04	1.04	1.04
Family income (ref. High)									
Low income				0.87	0.86	0.87	0.95	0.94	0.96
Middle–low income				1.01	1.00	1.02	1.04	1.03	1.05
Middle–high income				1.02	1.01	1.03	1.03	1.02	1.03
Educational level (ref. > 12 years)									
10–11 years				1.13	1.12	1.14	1.08	1.06	1.09
≥ 12 years				1.04	1.03	1.05	1.03	1.02	1.04
Socioeconomic status (ref. professionals)									
Farmers/self-employed/others				1.30	1.28	1.31	1.20	1.19	1.22
Blue collar workers				1.25	1.24	1.27	1.12	1.11	1.13
White collar workers				1.08	1.07	1.10	1.03	1.02	1.04
Region of residence (ref. Large cities)									
Southern Sweden				1.03	1.03	1.04	1.03	1.03	1.04
Northern Sweden				1.06	1.06	1.07	1.04	1.03	1.05
Immigrant status (ref. Born in Sweden)				0.97	0.96	0.99	0.98	0.96	1.00
Marital status (ref. Not married)				1.13	1.12	1.14	1.10	1.09	1.11
Birth order				1.00	1.00	1.01	1.01	1.01	1.01
Hospitalization of chronic lower respiratory disease (ref. Non)							1.38	1.36	1.39
Hospitalization of alcoholisms and related liver disease (ref. Non)							1.67	1.65	1.69
Hospitalization of diabetes (ref. Non)							1.38	1.37	1.39
Hospitalization of obesity (ref. Non)							1.32	1.30	1.34
Hospitalization of hypertension (ref. Non)							4.86	4.83	4.89
Cancer (ref. Non)							1.62	1.61	1.63

Model 1. Adjusted for age at start; Model 2. Adjusted for age at start and individual characteristics; Model 3. Model 2 + comorbidities.
*: Multivariable competing risk survival analysis

For peer review only

Supplementary Table 2d. Hazard ratio (HR) and 95% confidence interval of CVD in *women*, using multivariable competing risk survival analysis

	Model 1			Model 2			Model 3		
	HR*	95% CI		HR*	95% CI		HR*	95% CI	
Birth order (ref. First birth)									
Second	1.00	0.99	1.01	0.99	0.98	1.00	1.01	1.00	1.02
Third	1.03	1.02	1.04	1.00	0.99	1.01	1.02	1.01	1.03
Fourth	1.06	1.04	1.07	1.01	1.00	1.03	1.04	1.02	1.05
Fifth+	1.09	1.07	1.10	1.01	0.99	1.03	1.05	1.03	1.07
Age (years)	1.07	1.07	1.07	1.06	1.06	1.06	1.04	1.04	1.04
Family income (ref. High)									
Low income				0.87	0.86	0.87	0.95	0.94	0.96
Middle–low income				1.01	1.00	1.02	1.04	1.03	1.05
Middle–high income				1.02	1.01	1.03	1.03	1.02	1.03
Educational level (ref. > 12 years)									
10–11 years				1.13	1.12	1.14	1.08	1.06	1.09
≥ 12 years				1.04	1.03	1.05	1.03	1.02	1.04
Socioeconomic status (ref. professionals)									
Farmers/self-employed/others				1.30	1.28	1.31	1.21	1.19	1.22
Blue collar workers				1.26	1.24	1.27	1.12	1.11	1.13
White collar workers				1.09	1.07	1.10	1.03	1.02	1.05
Region of residence (ref. Large cities)									
Southern Sweden				1.03	1.03	1.04	1.03	1.03	1.04
Northern Sweden				1.06	1.06	1.07	1.04	1.03	1.05
Immigrant status (ref. Born in Sweden)				0.97	0.96	0.99	0.98	0.96	1.00
Marital status (ref. Not married)				1.13	1.13	1.14	1.10	1.09	1.11
Number of siblings				1.01	1.01	1.01	1.00	1.00	1.01
Hospitalization of chronic lower respiratory disease (ref. Non)							1.38	1.36	1.39
Hospitalization of alcoholisms and related liver disease (ref. Non)							1.67	1.65	1.69
Hospitalization of diabetes (ref. Non)							1.38	1.37	1.39
Hospitalization of obesity (ref. Non)							1.32	1.30	1.34
Hospitalization of hypertension (ref. Non)							4.86	4.83	4.89
Cancer (ref. Non)							1.62	1.61	1.63

Model 1. Adjusted for age at start; Model 2. Adjusted for age at start and individual characteristics; Model 3. Model 2 + comorbidities.
*: Multivariable competing risk survival analysis

For peer review only

Supplementary Table 3a. Hazard ratio (HR) and 95% confidence interval of CHD in *men*

	Model 1			Model 2			Model 3		
	HR	95% CI		HR	95% CI		HR	95% CI	
Number of siblings (ref. No sibling)									
One sibling	1.00	0.98	1.02	0.99	0.97	1.01	0.99	0.97	1.01
Two siblings	1.04	1.03	1.06	1.00	0.98	1.02	1.01	0.99	1.03
Three siblings	1.12	1.10	1.14	1.04	1.02	1.06	1.04	1.02	1.07
Four or more children	1.27	1.25	1.30	1.10	1.08	1.12	1.10	1.07	1.12
Age (years)	1.08	1.08	1.08	1.08	1.08	1.08	1.07	1.07	1.07
Family income (ref. High)									
Low income				0.93	0.91	0.94	0.96	0.95	0.98
Middle–low income				1.00	0.98	1.01	1.02	1.00	1.03
Middle–high income				1.01	1.00	1.03	1.02	1.00	1.03
Educational level (ref. > 12 years)									
10–11 years				1.05	1.03	1.07	1.04	1.02	1.07
≥ 12 years				0.94	0.93	0.95	0.95	0.94	0.96
Socioeconomic status (ref. professionals)									
Farmers/self-employed/others				1.23	1.20	1.25	1.18	1.16	1.21
Blue collar workers				1.26	1.24	1.28	1.21	1.19	1.23
White collar workers				1.18	1.16	1.20	1.15	1.13	1.17
Region of residence (ref. Large cities)									
Southern Sweden				1.11	1.10	1.12	1.12	1.11	1.14
Northern Sweden				1.32	1.30	1.34	1.31	1.29	1.33
Immigrant status (ref. Born in Sweden)				1.09	1.05	1.13	1.10	1.06	1.14
Marital status (ref. Not married)				1.00	0.99	1.01	0.99	0.98	1.01
Birth order				1.05	1.04	1.05	1.05	1.05	1.06
Hospitalization of chronic lower respiratory disease (ref. Non)							1.29	1.26	1.31
Hospitalization of alcoholisms and related liver disease (ref. Non)							1.01	0.99	1.04
Hospitalization of diabetes (ref. Non)							1.75	1.73	1.78
Hospitalization of obesity (ref. Non)							1.13	1.09	1.17
Hospitalization of hypertension (ref. Non)							1.86	1.84	1.88
Cancer (ref. Non)							0.91	0.89	0.92

Model 1. Adjusted for age at start; Model 2. Adjusted for age at start and individual characteristics; Model 3. Model 2 + comorbidities.

For peer review only

Supplementary Table 3b. Hazard ratio (HR) and 95% confidence interval of CHD in *men*

	Model 1			Model 2			Model 3		
	HR	95% CI		HR	95% CI		HR	95% CI	
Birth order (ref. First birth)									
Second	1.09	1.08	1.10	1.06	1.05	1.07	1.08	1.06	1.09
Third	1.19	1.17	1.21	1.11	1.09	1.13	1.13	1.11	1.15
Fourth	1.28	1.25	1.32	1.15	1.12	1.19	1.17	1.14	1.21
Fifth+	1.44	1.39	1.48	1.22	1.17	1.26	1.23	1.19	1.28
Age (years)	1.08	1.08	1.09	1.08	1.08	1.08	1.07	1.07	1.07
Family income (ref. High)									
Low income				0.93	0.91	0.94	0.96	0.95	0.98
Middle–low income				1.00	0.98	1.01	1.02	1.00	1.03
Middle–high income				1.01	1.00	1.02	1.02	1.00	1.03
Educational level (ref. > 12 years)									
10–11 years				1.05	1.03	1.08	1.05	1.03	1.07
≥ 12 years				0.94	0.93	0.95	0.95	0.94	0.97
Socioeconomic status (ref. professionals)									
Farmers/self-employed/others				1.23	1.20	1.25	1.18	1.16	1.21
Blue collar workers				1.26	1.24	1.28	1.21	1.19	1.23
White collar workers				1.18	1.16	1.20	1.15	1.13	1.17
Region of residence (ref. Large cities)									
Southern Sweden				1.11	1.10	1.13	1.12	1.11	1.14
Northern Sweden				1.32	1.30	1.34	1.31	1.29	1.33
Immigrant status (ref. Born in Sweden)				1.09	1.05	1.13	1.10	1.06	1.14
Marital status (ref. Not married)				1.00	0.99	1.01	0.99	0.98	1.01
Number of siblings				1.02	1.02	1.02	1.02	1.02	1.02
Hospitalization of chronic lower respiratory disease (ref. Non)							1.29	1.26	1.31
Hospitalization of alcoholisms and related liver disease (ref. Non)							1.01	0.99	1.04
Hospitalization of diabetes (ref. Non)							1.75	1.73	1.78
Hospitalization of obesity (ref. Non)							1.13	1.09	1.17
Hospitalization of hypertension (ref. Non)							1.86	1.84	1.88
Cancer (ref. Non)							0.91	0.89	0.92

Model 1. Adjusted for age at start; Model 2. Adjusted for age at start and individual characteristics; Model 3. Model 2 + comorbidities.

For peer review only

Supplementary Table 3c. Hazard ratio (HR) and 95% confidence interval of CHD in *women*

	Model 1			Model 2			Model 3		
	HR	95% CI		HR	95% CI		HR	95% CI	
Number of siblings (ref. No sibling)									
One sibling	0.99	0.97	1.02	0.98	0.96	1.01	0.99	0.97	1.02
Two siblings	1.07	1.04	1.10	1.03	1.00	1.05	1.03	1.00	1.06
Three siblings	1.17	1.13	1.20	1.08	1.04	1.11	1.07	1.04	1.11
Four or more children	1.40	1.36	1.44	1.20	1.16	1.24	1.17	1.13	1.21
Age (years)	1.09	1.09	1.09	1.09	1.08	1.09	1.07	1.07	1.07
Family income (ref. High)									
Low income				0.85	0.83	0.88	0.91	0.88	0.93
Middle–low income				1.00	0.98	1.03	1.00	0.98	1.03
Middle–high income				1.02	1.00	1.05	1.02	0.99	1.04
Educational level (ref. > 12 years)									
10–11 years				1.07	1.04	1.10	1.06	1.03	1.09
≥ 12 years				0.92	0.90	0.94	0.94	0.92	0.96
Socioeconomic status (ref. professionals)									
Farmers/self-employed/others				1.52	1.46	1.59	1.37	1.31	1.42
Blue collar workers				1.47	1.42	1.53	1.33	1.28	1.38
White collar workers				1.19	1.14	1.23	1.14	1.10	1.18
Region of residence (ref. Large cities)									
Southern Sweden				1.14	1.12	1.16	1.15	1.13	1.17
Northern Sweden				1.40	1.37	1.44	1.38	1.34	1.41
Immigrant status (ref. Born in Sweden)				1.13	1.07	1.20	1.13	1.07	1.20
Marital status (ref. Not married)				1.12	1.10	1.14	1.08	1.06	1.10
Birth order				1.04	1.03	1.05	1.05	1.04	1.06
Hospitalization of chronic lower respiratory disease (ref. Non)							1.69	1.65	1.73
Hospitalization of alcoholisms and related liver disease (ref. Non)							1.17	1.11	1.23
Hospitalization of diabetes (ref. Non)							2.20	2.15	2.25
Hospitalization of obesity (ref. Non)							1.11	1.06	1.17
Hospitalization of hypertension (ref. Non)							2.22	2.18	2.26
Cancer (ref. Non)							0.94	0.93	0.96

Model 1. Adjusted for age at start; Model 2. Adjusted for age at start and individual characteristics; Model 3. Model 2 + comorbidities.

For peer review only

Supplementary Table 3d. Hazard ratio (HR) and 95% confidence interval of CHD in *women*

	Model 1			Model 2			Model 3		
	HR	95% CI		HR	95% CI		HR	95% CI	
Birth order (ref. First birth)									
Second	1.10	1.07	1.12	1.05	1.02	1.07	1.07	1.05	1.09
Third	1.24	1.21	1.28	1.11	1.08	1.14	1.14	1.11	1.18
Fourth	1.33	1.27	1.38	1.11	1.06	1.16	1.14	1.09	1.20
Fifth+	1.56	1.48	1.63	1.17	1.11	1.24	1.22	1.15	1.29
Age (years)	1.09	1.09	1.09	1.09	1.08	1.09	1.07	1.07	1.07
Family income (ref. High)									
Low income				0.85	0.83	0.88	0.91	0.88	0.93
Middle–low income				1.00	0.98	1.03	1.01	0.98	1.03
Middle–high income				1.02	1.00	1.05	1.02	0.99	1.04
Educational level (ref. > 12 years)									
10–11 years				1.07	1.04	1.10	1.06	1.03	1.09
≥ 12 years				0.92	0.90	0.94	0.94	0.92	0.96
Socioeconomic status (ref. professionals)									
Farmers/self-employed/others				1.52	1.46	1.59	1.37	1.31	1.42
Blue collar workers				1.47	1.42	1.53	1.33	1.28	1.38
White collar workers				1.19	1.14	1.23	1.14	1.10	1.18
Region of residence (ref. Large cities)									
Southern Sweden				1.14	1.12	1.17	1.15	1.13	1.17
Northern Sweden				1.40	1.37	1.44	1.38	1.34	1.41
Immigrant status (ref. Born in Sweden)				1.13	1.07	1.20	1.13	1.07	1.20
Marital status (ref. Not married)				1.12	1.10	1.14	1.08	1.06	1.10
Number of siblings				1.04	1.03	1.04	1.03	1.03	1.04
Hospitalization of chronic lower respiratory disease (ref. Non)							1.69	1.65	1.73
Hospitalization of alcoholisms and related liver disease (ref. Non)							1.17	1.11	1.23
Hospitalization of diabetes (ref. Non)							2.20	2.15	2.25
Hospitalization of obesity (ref. Non)							1.11	1.06	1.17
Hospitalization of hypertension (ref. Non)							2.22	2.18	2.26
Cancer (ref. Non)							0.94	0.93	0.96

Model 1. Adjusted for age at start; Model 2. Adjusted for age at start and individual characteristics; Model 3. Model 2 + comorbidities.

For peer review only

Supplementary Table 4a. Hazard ratio (HR) and 95% confidence interval of mortality in *men*

	Model 1			Model 2			Model 3		
	HR	95% CI		HR	95% CI		HR	95% CI	
Number of siblings (ref. No sibling)									
One sibling	0.91	0.90	0.92	0.92	0.91	0.93	0.93	0.92	0.94
Two siblings	0.92	0.91	0.93	0.91	0.90	0.92	0.91	0.90	0.92
Three siblings	0.96	0.94	0.97	0.93	0.92	0.94	0.93	0.92	0.94
Four or more children	1.03	1.01	1.04	0.96	0.94	0.97	0.96	0.94	0.97
Age (years)	1.10	1.10	1.10	1.11	1.11	1.11	1.10	1.10	1.10
Family income (ref. High)									
Low income				1.05	1.04	1.07	1.08	1.07	1.10
Middle–low income				1.17	1.15	1.18	1.14	1.13	1.15
Middle–high income				1.18	1.17	1.19	1.15	1.14	1.16
Educational level (ref. > 12 years)									
10–11 years				1.12	1.10	1.13	1.08	1.06	1.09
≥ 12 years				0.91	0.90	0.91	0.89	0.88	0.90
Socioeconomic status (ref. professionals)									
Farmers/self-employed/others				1.73	1.71	1.76	1.63	1.60	1.65
Blue collar workers				1.38	1.36	1.40	1.34	1.32	1.36
White collar workers				1.15	1.13	1.17	1.14	1.12	1.16
Region of residence (ref. Large cities)									
Southern Sweden				0.94	0.93	0.95	0.99	0.98	1.00
Northern Sweden				0.98	0.97	0.99	1.08	1.07	1.09
Immigrant status (ref. Born in Sweden)				1.04	1.01	1.07	1.06	1.03	1.09
Marital status (ref. Not married)				1.88	1.87	1.90	1.71	1.70	1.73
Birth order				1.00	1.00	1.01	0.99	0.99	1.00
Hospitalization of chronic lower respiratory disease (ref. Non)							1.28	1.26	1.30
Hospitalization of alcoholisms and related liver disease (ref. Non)							3.02	2.98	3.05
Hospitalization of diabetes (ref. Non)							1.43	1.41	1.45
Hospitalization of obesity (ref. Non)							1.09	1.06	1.13
Hospitalization of hypertension (ref. Non)							0.66	0.65	0.67
Cancer (ref. Non)							2.62	2.60	2.65

Model 1. Adjusted for age at start; Model 2. Adjusted for age at start and individual characteristics; Model 3. Model 2 + comorbidities.

For peer review only

Supplementary Table 4b. Hazard ratio (HR) and 95% confidence interval of mortality in *men*

	Model 1			Model 2			Model 3		
	HR	95% CI		HR	95% CI		HR	95% CI	
Birth order (ref. First birth)									
Second	0.98	0.97	0.99	0.97	0.96	0.98	0.96	0.95	0.97
Third	1.01	0.99	1.02	0.99	0.98	1.01	0.98	0.96	0.99
Fourth	1.04	1.01	1.06	1.00	0.98	1.03	0.98	0.95	1.00
Fifth+	1.11	1.08	1.14	1.06	1.02	1.09	1.01	0.98	1.05
Age (years)	1.10	1.10	1.10	1.11	1.11	1.11	1.10	1.10	1.10
Family income (ref. High)									
Low income				1.06	1.04	1.07	1.08	1.07	1.10
Middle–low income				1.17	1.15	1.18	1.14	1.13	1.16
Middle–high income				1.18	1.17	1.19	1.15	1.14	1.16
Educational level (ref. > 12 years)									
10–11 years				1.12	1.10	1.13	1.07	1.06	1.09
≥ 12 years				0.90	0.90	0.91	0.89	0.88	0.90
Socioeconomic status (ref. professionals)									
Farmers/self-employed/others				1.73	1.71	1.76	1.63	1.60	1.66
Blue collar workers				1.38	1.36	1.40	1.34	1.32	1.36
White collar workers				1.15	1.13	1.17	1.14	1.13	1.16
Region of residence (ref. Large cities)									
Southern Sweden				0.94	0.93	0.95	0.99	0.98	1.00
Northern Sweden				0.98	0.97	0.99	1.08	1.07	1.09
Immigrant status (ref. Born in Sweden)				1.05	1.02	1.08	1.06	1.03	1.09
Marital status (ref. Not married)				1.88	1.87	1.90	1.71	1.70	1.73
Number of siblings				1.00	0.99	1.00	1.00	0.99	1.00
Hospitalization of chronic lower respiratory disease (ref. Non)							1.28	1.26	1.30
Hospitalization of alcoholisms and related liver disease (ref. Non)							3.02	2.98	3.05
Hospitalization of diabetes (ref. Non)							1.43	1.41	1.45
Hospitalization of obesity (ref. Non)							1.09	1.06	1.13
Hospitalization of hypertension (ref. Non)							0.66	0.65	0.67
Cancer (ref. Non)							2.62	2.60	2.64

Model 1. Adjusted for age at start; Model 2. Adjusted for age at start and individual characteristics; Model 3. Model 2 + comorbidities.

For peer review only

Supplementary Table 4c. Hazard ratio (HR) and 95% confidence interval of mortality in *women*

	Model 1			Model 2			Model 3		
	HR	95% CI		HR	95% CI		HR	95% CI	
Number of siblings (ref. No sibling)									
One sibling	0.92	0.91	0.94	0.93	0.92	0.95	0.94	0.93	0.95
Two siblings	0.92	0.91	0.94	0.91	0.90	0.93	0.92	0.91	0.94
Three siblings	0.95	0.93	0.96	0.92	0.90	0.93	0.93	0.91	0.95
Four or more children	1.01	0.99	1.03	0.94	0.92	0.95	0.95	0.93	0.96
Age (years)	1.10	1.10	1.10	1.10	1.10	1.10	1.09	1.08	1.09
Family income (ref. High)									
Low income				0.84	0.83	0.86	0.94	0.93	0.96
Middle–low income				1.03	1.01	1.05	1.06	1.04	1.07
Middle–high income				1.05	1.04	1.06	1.05	1.04	1.07
Educational level (ref. > 12 years)									
10–11 years				1.08	1.06	1.10	1.01	1.00	1.03
≥ 12 years				0.85	0.84	0.87	0.82	0.81	0.83
Socioeconomic status (ref. professionals)									
Farmers/self-employed/others				1.97	1.93	2.02	1.88	1.84	1.93
Blue collar workers				1.36	1.33	1.39	1.35	1.32	1.38
White collar workers				1.12	1.10	1.15	1.11	1.09	1.14
Region of residence (ref. Large cities)									
Southern Sweden				1.00	0.99	1.01	1.03	1.02	1.04
Northern Sweden				1.03	1.02	1.05	1.11	1.09	1.12
Immigrant status (ref. Born in Sweden)				0.89	0.86	0.92	0.95	0.92	0.99
Marital status (ref. Not married)				1.63	1.62	1.65	1.48	1.47	1.50
Birth order				1.01	1.00	1.01	1.00	0.99	1.01
Hospitalization of chronic lower respiratory disease (ref. Non)							1.61	1.58	1.63
Hospitalization of alcoholisms and related liver disease (ref. Non)							3.26	3.20	3.32
Hospitalization of diabetes (ref. Non)							1.58	1.56	1.61
Hospitalization of obesity (ref. Non)							0.96	0.93	0.99
Hospitalization of hypertension (ref. Non)							0.66	0.65	0.67
Cancer (ref. Non)							4.86	4.81	4.91

Model 1. Adjusted for age at start; Model 2. Adjusted for age at start and individual characteristics; Model 3. Model 2 + comorbidities.

For peer review only

Supplementary Table 4d. Hazard ratio (HR) and 95% confidence interval of mortality in *women*

	Model 1			Model 2			Model 3		
	HR	95% CI		HR	95% CI		HR	95% CI	
Birth order (ref. First birth)									
Second	0.98	0.96	0.99	0.97	0.96	0.98	0.96	0.95	0.98
Third	1.01	1.00	1.03	1.00	0.98	1.02	0.98	0.96	1.00
Fourth	1.05	1.02	1.08	1.02	0.99	1.05	1.00	0.98	1.03
Fifth+	1.11	1.08	1.15	1.08	1.04	1.12	1.03	0.99	1.07
Age (years)	1.10	1.10	1.10	1.10	1.10	1.10	1.09	1.08	1.09
Family income (ref. High)									
Low income				0.84	0.83	0.86	0.94	0.93	0.96
Middle–low income				1.03	1.02	1.05	1.06	1.04	1.07
Middle–high income				1.05	1.04	1.07	1.05	1.04	1.07
Educational level (ref. > 12 years)									
10–11 years				1.08	1.06	1.10	1.01	0.99	1.03
≥ 12 years				0.85	0.84	0.86	0.82	0.81	0.83
Socioeconomic status (ref. professionals)									
Farmers/self-employed/others				1.98	1.93	2.02	1.88	1.84	1.93
Blue collar workers				1.36	1.33	1.39	1.35	1.32	1.38
White collar workers				1.12	1.10	1.15	1.11	1.09	1.14
Region of residence (ref. Large cities)									
Southern Sweden				1.00	0.99	1.01	1.03	1.02	1.04
Northern Sweden				1.03	1.02	1.05	1.11	1.09	1.12
Immigrant status (ref. Born in Sweden)				0.89	0.86	0.92	0.95	0.92	0.99
Marital status (ref. Not married)				1.63	1.62	1.65	1.48	1.47	1.50
Number of siblings				0.99	0.99	0.99	0.99	0.99	1.00
Hospitalization of chronic lower respiratory disease (ref. Non)							1.61	1.58	1.63
Hospitalization of alcoholisms and related liver disease (ref. Non)							3.26	3.20	3.33
Hospitalization of diabetes (ref. Non)							1.58	1.56	1.61
Hospitalization of obesity (ref. Non)							0.96	0.93	0.99
Hospitalization of hypertension (ref. Non)							0.66	0.65	0.67
Cancer (ref. Non)							4.86	4.81	4.91

Model 1. Adjusted for age at start; Model 2. Adjusted for age at start and individual characteristics; Model 3. Model 2 + comorbidities.

For peer review only

STROBE 2007 (v4) Statement—Checklist of items that should be included in reports of *cohort studies*

Section/Topic	Item #	Recommendation	Reported on page #
**Title and abstract**	1	(*a*) Indicate the study's design with a commonly used term in the title or the abstract	1
		(*b*) Provide in the abstract an informative and balanced summary of what was done and what was found	2
**Introduction**			
Background/rationale	2	Explain the scientific background and rationale for the investigation being reported	3
Objectives	3	State specific objectives, including any prespecified hypotheses	3
**Methods**			
Study design	4	Present key elements of study design early in the paper	3-5
Setting	5	Describe the setting, locations, and relevant dates, including periods of recruitment, exposure, follow-up, and data collection	3-5
Participants	6	(*a*) Give the eligibility criteria, and the sources and methods of selection of participants. Describe methods of follow-up	3-5, and Figure S1
		(*b*) For matched studies, give matching criteria and number of exposed and unexposed	NA
Variables	7	Clearly define all outcomes, exposures, predictors, potential confounders, and effect modifiers. Give diagnostic criteria, if applicable	4
Data sources/ measurement	8*	For each variable of interest, give sources of data and details of methods of assessment (measurement). Describe comparability of assessment methods if there is more than one group	*4-5*
Bias	9	Describe any efforts to address potential sources of bias	4-5
Study size	10	Explain how the study size was arrived at	3
Quantitative variables	11	Explain how quantitative variables were handled in the analyses. If applicable, describe which groupings were chosen and why	4
Statistical methods	12	(*a*) Describe all statistical methods, including those used to control for confounding	5
		(*b*) Describe any methods used to examine subgroups and interactions	5
		(*c*) Explain how missing data were addressed	5
		(*d*) If applicable, explain how loss to follow-up was addressed	5
		(*e*) Describe any sensitivity analyses	5
**Results**			
Participants	13*	(a) Report numbers of individuals at each stage of study—eg numbers potentially eligible, examined for eligibility, confirmed eligible, included in the study, completing follow-up, and analysed	5

For peer review only

		(b) Give reasons for non-participation at each stage	NA
		(c) Consider use of a flow diagram	
Descriptive data	14*	(a) Give characteristics of study participants (eg demographic, clinical, social) and information on exposures and potential confounders	5, Table 1, and Supplementary Table 1a and 1b
		(b) Indicate number of participants with missing data for each variable of interest	NA
		(c) Summarise follow-up time (eg, average and total amount)	5
Outcome data	15*	Report numbers of outcome events or summary measures over time	5, Table 1, and Supplementary Table 1a, and 1b
Main results	16	(*a*) Give unadjusted estimates and, if applicable, confounder-adjusted estimates and their precision (eg, 95% confidence interval). Make clear which confounders were adjusted for and why they were included	5-6, Table 2 and 3, and Supplementary Tables 2a-2d, 3a-3d, and 4a-4d.
		(*b*) Report category boundaries when continuous variables were categorized	NA
		(*c*) If relevant, consider translating estimates of relative risk into absolute risk for a meaningful time period	NA
Other analyses	17	Report other analyses done—eg analyses of subgroups and interactions, and sensitivity analyses	5-6
**Discussion**			
Key results	18	Summarise key results with reference to study objectives	3, 7
**Limitations**			
Interpretation	20	Give a cautious overall interpretation of results considering objectives, limitations, multiplicity of analyses, results from similar studies, and other relevant evidence	3, 7, 8
Generalisability	21	Discuss the generalisability (external validity) of the study results	9
**Other information**			
Funding	22	Give the source of funding and the role of the funders for the present study and, if applicable, for the original study on which the present article is based	9

*Give information separately for cases and controls in case-control studies and, if applicable, for exposed and unexposed groups in cohort and cross-sectional studies.

Note: An Explanation and Elaboration article discusses each checklist item and gives methodological background and published examples of transparent reporting. The STROBE checklist is best used in conjunction with this article (freely available on the Web sites of PLoS Medicine at http://www.plosmedicine.org/, Annals of Internal Medicine at http://www.annals.org/, and Epidemiology at http://www.epidem.com/). Information on the STROBE Initiative is available at www.strobe-statement.org.

For peer review only
